# AOA-2 Derivatives as Outer Membrane Protein A Inhibitors for Treatment of Gram-Negative Bacilli Infections

**DOI:** 10.3389/fmicb.2021.634323

**Published:** 2021-02-12

**Authors:** Rafael Ayerbe-Algaba, Nuria Bayó, Ester Verdú, Raquel Parra-Millán, Jesús Seco, Meritxell Teixidó, Jerónimo Pachón, Ernest Giralt, Younes Smani

**Affiliations:** ^1^Clinical Unit of Infectious Diseases, Microbiology and Preventive Medicine, University Hospital Virgen del Rocío, Seville, Spain; ^2^Institute of Biomedicine of Seville (IBiS), University Hospital Virgen del Rocío/Spanish National Research Council (CSIC)/University of Seville, Seville, Spain; ^3^Institute for Research in Biomedicine (IRB Barcelona), Barcelona Institute for Science and Technology (BIST), Barcelona, Spain; ^4^Department of Medicine, University of Seville, Seville, Spain; ^5^Department of Inorganic and Organic Chemistry, University of Barcelona, Barcelona, Spain

**Keywords:** AOA-2 derivatives, outer membrane protein A, inhibitor, Gram-negative bacilli, peptides

## Abstract

Previously, we identified that a cyclic hexapeptide AOA-2 inhibited the interaction of Gram-negative bacilli (GNB) like *Acinetobacter baumannii*, *Pseudomonas aeruginosa*, and *Escherichia coli* to host cells thereby preventing the development of infection *in vitro* and in a murine sepsis peritoneal model. In this work, we aimed to evaluate *in vitro* a library of AOA-2 derivatives in order to improve the effect of AOA-2 against GNB infections. Ten AOA-2 derivatives were synthetized for the *in vitro* assays. Their toxicities to human lung epithelial cells (A549 cells) for 24 h were evaluated by determining the A549 cells viability using 3-(4,5-dimethylthiazol-2-yl)-2,5-diphenyltetrazolium bromide assay. The effect of these peptide derivatives and AOA-2 at 250, 125, 62.5, and 31.25 μg/mL on the attachment of *A. baumannii* ATCC 17978, *P. aeruginosa* PAO1 and *E. coli* ATCC 25922 strains to A549 cells was characterized by adherence and viability assays. None of the 10 derivatives showed toxicity to A549 cells. RW01 and RW06 have reduced more the adherence of ATCC 17978, PAO1 and ATCC 2599 strains to A549 cells when compared with the original compound AOA-2. Moreover, both peptides have increased slightly the viability of infected A549 cells by PAO1 and ATCC 25922 than those observed with AOA-2. Finally, RW01 and RW06 have potentiated the activity of colistin against ATCC 17978 strain in the same level with AOA-2. The optimization program of AOA-2 has generated two derivatives (RW01 and RW06) with best effect against interaction of GNB with host cells, specifically against *P. aeruginosa* and *E. coli*.

## Introduction

Multidrug resistance (MDR) in Gram-negative bacilli (GNB) is a current threat in global health that requires effective and urgent solutions, being a global priority for WHO (Tacconelli et al., 2018). The success of GNB as a community and nosocomial pathogen is attributed to its resistant to several antibiotic families. Whole-genome sequencing studies involving the GNB have demonstrated not only a vast array of antibiotic drug resistance determinants but also many pathogenicity islands (Kaper et al., 2004; Smith M. G. et al., 2007).

Studies of specific virulence mechanisms have demonstrated the contribution of outer membrane protein A (OmpA), as well as pili, fimbriae, outer membrane proteins and various secretion systems in the pathogenesis of GNB *in vitro* and in animal experimental models (Weiser and Gotschlich, 1991; Prasadarao et al., 1996; Wu et al., 2003; Teng et al., 2006; Mittal et al., 2011; Sánchez-Encinales et al., 2017; Nicholson et al., 2019).

OmpA is a β-barrel porin highly conserved among bacterial species, especially among Gram-negative organisms (Smith S. G. J. et al., 2007). This protein has a variety of interesting biological properties. It has been suggested to have involvement in the adherence to epithelia (Gaddy et al., 2009), translocation into the epithelial cell nucleus (Choi et al., 2008), induction of epithelial cell death (Choi et al., 2005), biofilm formation (Gaddy et al., 2009), and binding to factor H, which may allow *A. baumannii* to develop serum resistance (Kim et al., 2009).

Previous studies showed that *A. baumannii* and *E. coli* deficient in *ompA* reduced their pathogenecities *in vitro* and *in vivo* (Mittal et al., 2011; Sánchez-Encinales et al., 2017). Interestingly, we showed that the inhibition of OmpA by a cyclic hexapetide AOA-2 reduces bacterial dissemination between organs, development of pneumonia, and bacteremia and death in murine peritoneal sepsis model by *A. baumannii*, *P. aeruginosa*, and *E. coli* (Vila-Farrés et al., 2017). Moreover, AOA-2 has showed *in vitro* synergy with colistin which has presented a good therapeutic efficacy against colistin-susceptible and resistant *A. baumannii* through the overexpression of Omp25 (Parra-Millán et al., 2018). In the present study, we aimed to improve the efficacy of AOA-2 against GNB infections by optimizing the derivatives of AOA-2.

## Materials and Methods

### Materials for the Synthesis of Peptides

The protected amino acids and resins were supplied by Luxembourg Industries (Tel-Aviv, Israel), Neosystem (Strasbourg, France), Calbiochem-Novabiochem AG (Läufelfingen, Switzerland), PolyPeptides Labs (Torrance, CA, United States), Bachem AG (Bubendorf, Switzerland), and Iris Biotech (Marktredwitz, Germany). PyBOP was provided by Calbiochem-Novabiochem AG. Piperidine is obtained from SDS (Peypin, France); N, N-diisopropylethylamine (DIEA) was obtained from Merck (Darmstadt, Germany) and triisopropylsilane (TIS) and ninhydrin were from Fluka Chemika (Buchs, Switzerland). HOAt was acquired from GL Biochem Shanghai Ltd. (Shanghai, China). Solvents for the synthesis of peptides and RP-HPLC [dimethylformamide (DMF), dichloromethane (DCM) and acetonitrile (MeCN)] were from Scharlau or SDS (Barcelona, Spain). Trifluoroacetic acid (TFA) was purchased from Kali Chemie (Bad Wimpfen, Germany). The other chemicals used were from Aldrich (Milwaukee, WI, United States) and were of the highest commercially available purity.

### Synthesis of Peptides

The peptides were synthesized on a 2-Chlorotrythyl chloride resin by solid-phase peptide synthesis using the 9-fluorenylmethoxycarbonyl/tert-butyl (Fmoc/tBu) strategy. The amino acids protected with N^α^ -Fmoc (3 eq)/HOAt (3 eq), PyBOP (4 eq), and DIEA (6 eq) were used for the couplings. The Fmoc protecting group was removed by treatment with a 20% piperidine solution in DMF. The peptides were cleaved using 2% TFA in DCM. For the cyclization step, the solvent used was DCM/DMF (98:2), PyAOP (2 eq) was dissolved in DMF and the peptide (5 mM) in DCM and once mixed in the appropriate proportions, 6 eq of DIEA was added, the reaction was completed in 2 or 3 h. After the cyclization carried out, deprotection of the side chains was carried out by TFA/TIS/H_2_O (95:2.5:2.5). Peptides were analyzed at λ = 220 nm by HPLC [Waters Alliance 2695 separation module equipped with a 2998 photodiode array detector, Sunfire C18 column (100 mm × 4.6 mm × 3.5 mm, 100 A, Waters)], and Empower software; flow rate = 1 ml/min. Peptides were then purified by semi-preparative HPLC [Waters 2700 Sample Manager equipped with a double absorbance detector λ Waters 2487, a Waters 600 controller, a Waters fraction collection II, a Symmetry C_18_ column (100 mm × 30 mm, 5 mm, 100 Å, Waters) and the Millenium chromatography manager software]. Flow rate = 15 mL/min; solvents: A = 0.1% trifluoroacetic acid in water, and B = 0.05% trifluoroacetic acid in acetonitrile. Peptides are characterized by MALDI-TOF mass spectrometry (Voyager-DE RP MALDI-TOF, PE Biosystems with a N_2_-laser of 337 nm) and a high-resolution ESI-MS model (LTQ-FT Ultra, Thermo Fisher Scientific).

### Docking and Molecular Modeling

All molecular docking calculations, and preparation of protein and ligand systems were performed using Molecular Operating Environment (MOE; Chemical Computing Group, Quebec, Canada) software. Protein structure of the transmembrane domain of *A. baumannii* OmpA was obtained by homology modeling using Comparative Modeling with Rosetta (RosettaCM) (Song et al., 2013) using as template PDBs obtained by NMR (2GE4, 1G90) and crystallography (1BXW, 1QJP). Best-ranked homology models were minimized and further relaxed including short (2 ns) explicit solvent molecular dynamics (MD) simulations using Amber99 force field. To identify druggable pockets of interest and obtain a pharmacophoric description of the binding site, a modification of the original MDMix method (Seco et al., 2009) was applied. Briefly, the original isopropanol probe was replaced by alternative probe molecules compatible with the sidechain peptide chemistry, including as probes metylammonium, benzene and propane. The pharmacophore coordinates were profiled in terms of binding energetics and clustered to delimit the docking grid box extension and simultaneously, guide docking exploration toward compatible solutions. The process of pharmacophore identification and clustering was conducted with an in-house Python script. A library of cyclic hexapeptides was used for virtual screening. All initial cyclic peptide structures were subjected to conformational exploration (Chen and Foloppe, 2008) by using MOE, clustering the low-energy conformations with a backbone RMSD threshold of 0.25 units to yield a pool of conformations ranging between 10 and 25 per cyclic peptide structure. All generated conformations were docked using a hierarchical docking protocol using rDock (Ruiz-Carmona et al., 2014) and results were re-scored with AutoDock Vina.

### Bacterial Strains

Three reference bacterial strains acquired from American Type Culture Collection (LGC, United Kingdom) were used: (a) *A. baumannii* ATCC 17978 (Baumann et al., 1968), (b) *P. aeruginosa* PAO1 (Holloway, 1955), and (c) *E. coli* ATCC 25922 (Reading and Cole, 1977). An OmpA-deficient mutant *A. baumannii*, JPAB01, was used (Parra-Millán et al., 2018).

### Cell Culture and Infection

The human pneumocyte cell line type II A549, derived from a human lung carcinoma, obtained from the American Type Culture Collection was used and cultured in DMEM medium (Invitrogen, Spain) supplemented with 10% of heat-inactivated fetal bovine serum, 50 μg/mL vancomycin (Laboratorios Normon, Spain), 20 μg/mL gentamicin (Normon Laboratories, Spain), 0.25 μg/mL amphotericin B mL (Invitrogen, Spain) and 1% HEPES (Invitrogen, Spain) in a humidified incubator, 5% CO_2_ at 37°C. Routine passage of A549 cells was performed every 3–4 days. Cells were seeded 24 h in 96-well plates (Sarstedt, Germany) for the cell viability assay, and in 24-well plates (Sarstedt, Germany) for the bacterial adhesion assays.

### *In vitro* Toxicity of Peptides

A549 cells were incubated with the peptide derivatives RW01, RW02, RW03, RW06, RW07, RW08, RW09, RW10, RW11, and RW13 (0, 3.5, 7, 15, 35, 62, 125, 250, and 500 μg/mL) for 24 h with 5% CO_2_ at 37°C. Prior the evaluation of the peptides cytotoxicity, A549 cells were washed three times with prewarmed PBS 1X. Subsequently, quantitative cytotoxicity was evaluated by measuring the mitochondrial reduction activity using the 3-(4,5-dimethylthiazol-2-yl)-2,5-diphenyltetrazolium bromide (MTT) assay as described previously (Smani et al., 2013). The percentage of cytotoxicity was calculated from the absorbance at 570 nm as follow: [(Absorbance 570 nm of treated cells/Absorbance 570 nm mean of untreated cells) × 100].

### Bacterial Adhesion Assay

*A. baumannii* ATCC 17978, *P. aeruginosa* PAO1 and *E. coli* ATCC 25922 strains were incubated with AOA-2 (31.25, 62.5, 125, and 250 μg/mL, 30 min), AOA-2 derivatives RW01, RW02, RW03, RW06, RW07, RW08, RW09, RW10, RW11, and RW13 (250 μg/mL, 30 min), or AOA-2 derivatives RW01, RW02, RW03, RW06 (31.25, 62.5, and 125 μg/mL, 30 min), and added at 10^8^ CFU/mL, corresponding to a multiplicity of infection (MOI) of 500, to the A549 cells for 2 h at 5% CO_2_ and 37°C. Subsequently, infected A549 cells were washed five times with prewarmed PBS 1X and lysed with 0.5% Triton X-100. Serial dilutions of these lysates were made and plated on blood agar plates (Blood-Agar Columbia, Becton Dickinson Microbiology Systems, United States) and incubated at 37°C for 24 h for colony counting and then determination of CFU/mL that attached to A549 cells.

### Bacterial Cytotoxicity

A549 cells were infected with 10^8^ CFU/mL, corresponding to a MOI of 500, of *A. baumannii* ATCC 17978, *P. aeruginosa* PAO1 and *E. coli* ATCC 25922 strains pretreated with AOA-2 or AOA-2 derivatives RW01, RW02, RW03, and RW06 (31.25, 62.5, and 125 μg/mL, 30 min) for 24 h with 5% CO_2_ at 37°C. Prior to the evaluation of bacterial cytotoxicity, viable bacteria are first removed from A549 cell culture by washing these A549 cells five times with PBS 1X. Subsequently, cell viability was evaluated as described above.

### *In vitro* Susceptibility Testing

The minimal inhibitory concentration (MIC) of colistin in absence and presence of RW01 or RW06 at 125μμg/mL for *A. baumannii* ATCC 17978 strain was determined by microdilution assay in two independent experiments as previously reported (Parra-Millán et al., 2018).

### Statistical Analysis

Group data are presented as mean ± SEM. Student *t*-test was used to determine differences between means. Differences were considered significant at *P* < 0.05. The SPSS (version 23.0) statistical package was used (SPSS Inc.).

## Results

### AOA-2 Derivatives Synthesis

The optimization of the AOA-2 peptide was carried out through an iterative computational design process. This computational design was based on the use of docking techniques with flexible side chains and molecular dynamics, using the data on the interaction of AOA-2 and OmpA in detailed (Vila-Farrés et al., 2017). Previously, an exhaustive exploration of its accessible conformational space was made for each molecule. All the interaction calculations were carried out thanks to the access of the “Mare Nostrum” supercomputer at the Barcelona Supercomputer Center (BSC). Thus, a total of 10 peptides derived from the lead compound, AOA-2 were synthesized (Table 1).

**TABLE 1:** Peptide sequences of the AOA-2 and its derivatives, and their docking score.

Peptides	Sequence	Rational	Docking score
AOA-2	&Trp-D-Pro-Arg-Trp-D-Pro-Arg&	Hit compound	−9.34
RW01	&Trp-(NH_2_) D-Pro-Arg-Trp-D-Pro-Arg&	Solubility improved	−9.51
RW02	&3Pal-D-Pro-Arg-3Pal-D-Pro-Arg&	Analogs of trytophan	−8.59
RW03	&2Nal-D-Pro-Arg-2Nal-D-Pro-Arg&	Analogs of trytophan	−9.02
RW06	&Trp-D-Pro-Dab-Trp-D-Pro-Dab&	Analogs of arginine	−9.39
RW07	&Trp-D-Pro-Lys-Trp-D-Pro-Lys&	Analogs of arginine	−8.71
RW08	&Arg-D-Pro-Trp-Trp-D-Pro-Arg&	Repositioning	−9.04
RW09	&Trp-D-Pro-Trp-Arg-D-Pro-Arg&	Repositioning	−9.18
RW10	&Tyr-D-Pro-Ala-Tyr-D-Pro-Ala&	Higher score	−9.90
RW11	&2Nal-D-Pro-Arg-3Pal-D-Pro-Arg&	Higher score	−9.68
RW13	&3Pal-D-Pro-Arg-Trp-D-Pro-Tyr&	Higher score	−9.92

**FIGURE 1:**
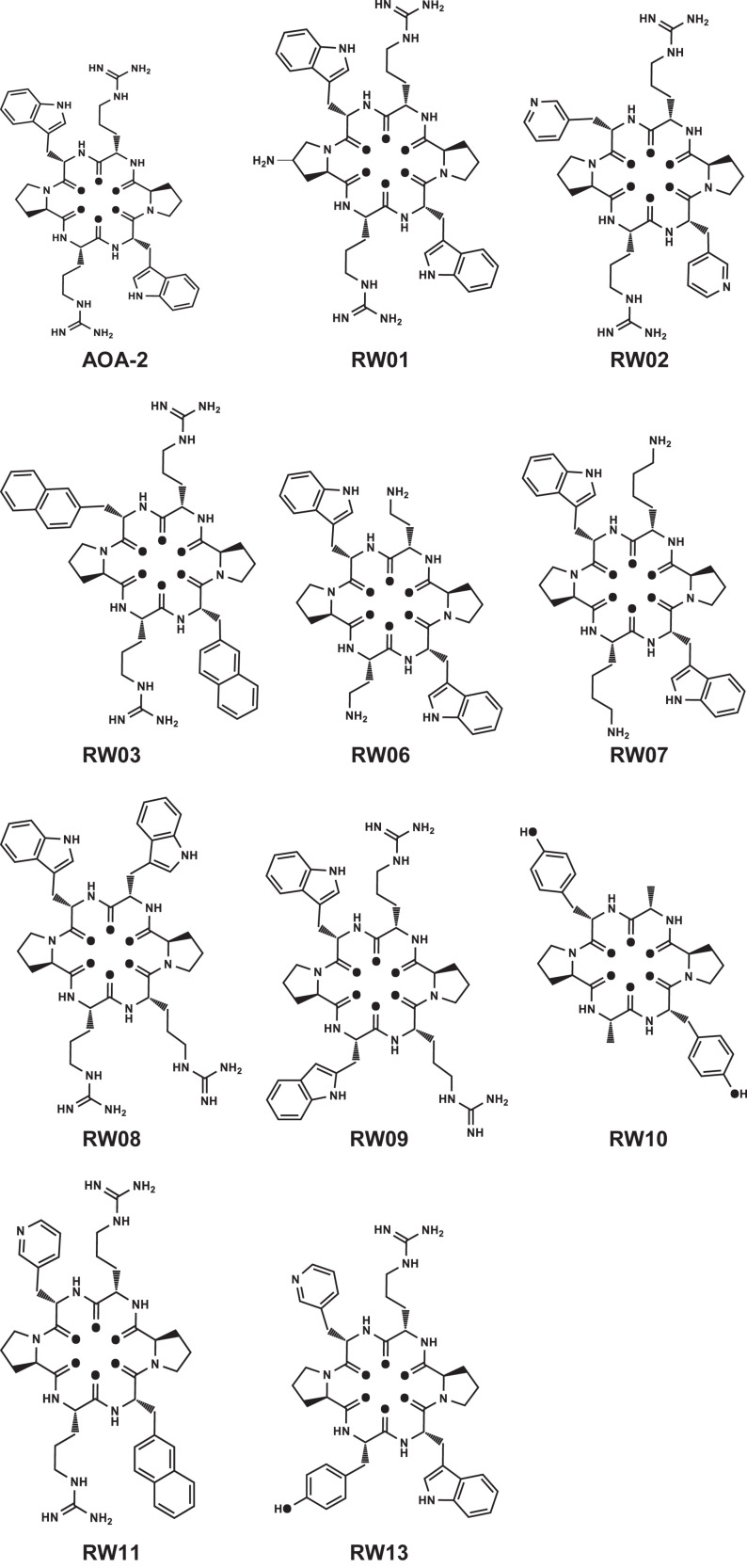
Structure of AOA-2 and its optimized derivatives.

Different changes were carried out to optimize the lead compound. In RW01 solubility was enhanced by the addition of NH_2_ group. In RW02 and RW03 tryptophan analogs [3Pal: beta-(3-pyridyl)-alanine, and 2Nal: beta-(2-naphtyl)-alanine] were added. In RW06 and RW07 arginine analogs (Dab: 2,4-Diaminobutanoic acid, and lysine) were added. In RW08 and RW09 a repositioning of the hit sequence were made. The remaining peptides RW10, RW11, and RW13 showed a higher docking score than the AOA-2 peptide (Table 1 and Figure 1).

### *In vitro* Toxicity of AOA-2 Derivatives

The study of the toxicity of the synthesized compounds was carried out. The percentage of cell viability on the A549 epithelial cells incubated for 24 h with the different peptides at decreasing concentrations was determined from 500 to 3.5 μg/mL. None of the studied compounds showed cytotoxicity at the different concentrations used, being the cellular viability is between 94 and 100% (Table 2).

**TABLE 2:** Percentages of A549 cellular toxicity assessed through mitochondrial reduction activity (MTT assay) in presence AOA-2 derivatives. Data expressed as a percentage of cell viability.

Concentration (μg/mL)								

	500	250	125	62	35	15	7	3.5
RW01	94.870.13	108.830.06	111.850.05	106.090.09	100.370.3	104.690.07	109.590.09	105.670.05
RW02	94.240.09	112.640.37	103.70.23	106.810.13	103.940.15	111.20.03	97.030.17	100.710.15
RW03	98.110.17	103.630.02	104.080.01	104.220.04	105.940.02	106.360.02	106.20.02	104.090.04
RW06	99.430.04	101.270.07	104.190.31	106.990.12	97.370.2	97.070.19	101.250.19	107.960.1
RW07	96.230.12	106.630.01	111.190.05	110.770.02	107.790.08	100.480.1	105.060.13	105.420.14
RW08	101.380.25	114.120.02	109.650.04	109.450.11	110.760.011	112.890.04	108.050.06	111.30.07
RW09	98.50.23	111.060.03	111.520.08	104.590.16	113.750.05	103.120.1	104.860.13	102.440.1
RW10	97.880.07	99.050.11	101.790.01	104.210.01	104.030.004	103.790.01	104.120.003	103.050.01
RW11	100.550.18	104.770.03	104.040.09	104.690.02	104.280.002	102.110.002	101.380.01	102.380.03
RW13	101.290.06	102.190.03	101.160.05	104.080.02	104.480.008	104.110.004	102.350.07	104.190.002

### *In vitro* Effect of AOA-2 Derivatives on *A. baumannii*, *P. aeruginosa*, *E. coli* Interaction With Host Cells

#### Bacterial Adhesion Assay

**FIGURE 2:**
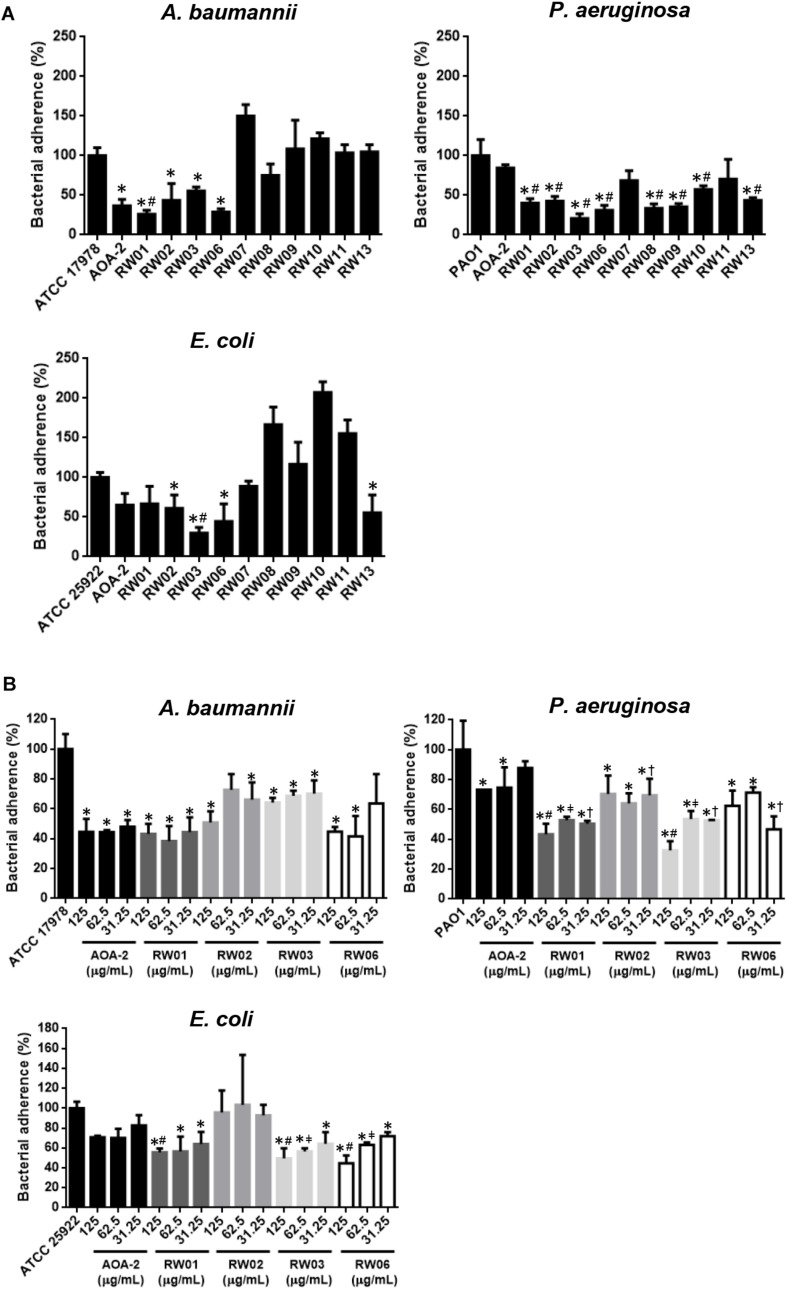
Bacterial adhesion assay of *A. baumannii* ATCC 17978, *P. aeruginosa* PAO1 and *E. coli* ATCC 25922 in presence of AOA-2 and its derivatives at concentration of 250 μg/mL **(A)**, and 31.25, 62.5, and 125 μg/mL **(B)**. ^∗^compared to the untreated bacteria, ^#^compared to AOA-2 at 125 or 250 μg/mL, ^μ^compared to AOA-2 at 62.5 μg/mL, ^†^compared to AOA-2 at 31.25 μg/mL, Student’s *t*-test with *P* < 0.05.

An initial stage of screening of the effect of all peptide derivatives and the original compound AOA-2 on adherence of *A. baumannii* ATCC 17978, *P. aeruginosa* PAO1 and *E. coli* ATCC 25922 strains to host cells was carried out. First we showed that AOA-2, RW01, RW02, RW03, and RW06 at 250 μg/mL reduced the adherence of *A. baumannii* to 36.62, 26.18, 43.58, 55.47, and 32.83%, respectively, the adherence of *P. aeruginosa* to 84.47, 40.08, 42.51, 20.71, and 31.14%, respectively and the adherence of *E. coli* to 65.05, 66.66, 61.11, 29.62, and 44.44%, respectively. In addition, we found that RW07 and RW13 reduced the adherence of PAO1 and ATCC 25922 strains to A549 cells; whereas, RW08 reduced the adherence of ATCC 17978 and PAO1 strains to A549 cells. In the case of RW09, RW10 and RW11, only the adherence of PAO1 strain to A549 cells was reduced (Figure 2A). From these data, we suggest that RW01, RW02, RW03 and RW06 present an inhibitory effect on the adherence of the three pathogens to A549 cells.

Next, RW01, RW02, RW03, and RW06 were selected for the following studies, in which the reduction of bacterial adherence was studied again but at decreasing concentrations of these peptides (125, 62.5, and 31.25 μg/mL) and compared with those of the original peptide AOA-2 (Figure 2B). We found that the four peptides reduced better the adherence of PAO1 strain with some statistical differences when compared with AOA-2. While, RW01, RW03, and RW06 have reduced with some statistical differences in comparison with AOA-2 the adherence of ATCC 25922 to host cells. Against ATCC 17978 strain, only RW01 and RW06 have reduced non-statistically the adherence of ATCC 17978 to A549 cells when compared with AOA-2 (except for treatment with RW06 at 31.25 μg/mL). From these data, we suggest that RW01 and RW06 improve the effect of AOA-2 on the adherence of *P. aeruginosa* and *E. coli* to host cells, and with slight level against *A. baumannii*.

#### Cell Viability Assay

**FIGURE 3:**
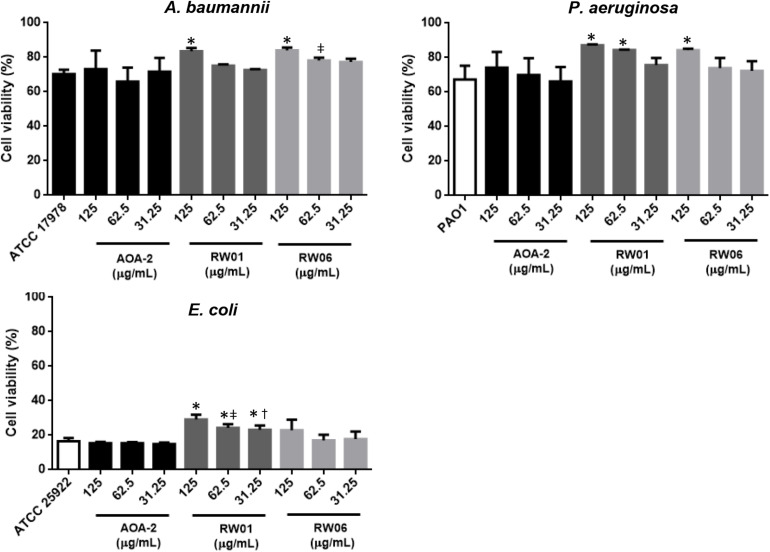
Cell viability assay of *A. baumannii* ATCC 17978, *P. aeruginosa* PAO1, and *E. coli* ATCC 25922 at different concentrations of AOA-2 and its derivatives RW01 and RW06. ^∗^compared to the untreated bacteria, ^μ^compared to AOA-2 at 62.5 μg/mL, ^†^compared to AOA-2 at 31.25 μg/mL, Student’s *t*-test with *P* < 0.05.

In order to confirm that AOA-2 derivatives have improved the effect of the original compound AOA-2 against *A. baumannii*, *P. aeruginosa*, and *E. coli*, only the effects of RW01 and RW06 vs. AOA-2 at 125, 62.5, and 31.25 μg/mL on cell viability in infected A549 cells were explored (Figure 3). Treatment of ATCC 17978, PAO1 and ATCC 25922 strains with AOA-2 at these concentrations did not increase significantly the cell viability with respect to untreated strains. Whereas, only the treatment of ATCC 17978 strain with RW01 or RW06 at 125 μg/mL increased significantly the cell viability with respect to untreated ATCC 17978 strain.

Similar results were observed with PAO1 and ATCC 25922 strains. Treatment of both strains with RW01 at the three concentrations increased significantly the cell viability with respect to untreated strains (except for PAO1 strain treatment with RW01 at 31.25 μg/mL). In contrast, treatment of both strains with RW06 (except for PAO1 treatment with RW06 at 125 at μg/mL) did not increase significantly the cell viability with respect to untreated strains.

Comparing the results of RW01 and RW06 with those of AOA-2, we found that only RW06 at 62.5 μg/mL has protected more significantly the cell death caused by ATCC 17978, whereas RW01 at 62.5 and 31.25 μg/mL has been more protective against ATCC 25922.

#### Synergistic Effect of AOA-2 Derivatives Against *A. baumannii*

We previously demonstrated that AOA-2 synergize with colistin against *A. baumannii* (Parra-Millán et al., 2018). Therefore, we thought it would be interesting to examine the capacity of RW01 and RW06 to potentiate the activity of colistin against *A. baumannii* ATCC 17978 strain. The addition of RW01 or RW06 at 125 μg/mL to ATCC 17978 strain increases the susceptibility of this strain to colistin from 0.5 to = 0.015 μg/mL. In contrast, the addition of RW01 or RW06 at 125 μg/mL to JPAB01 did not change the activity (MIC 0.0625 μg/mL) of colistin against this mutant strain. This last data suggest that RW01 and RW06 might act on OmpA of *A. baumannii*.

## Discussion

Nowadays the threat of antibiotic resistance requires a multiple approach that includes better administration of known antibiotics and the development of new alternative strategies to treat or prevent bacterial infections.

This study focuses on the paradigm of interference in pathogenesis, that is, disarming pathogenic bacteria by neutralizing their virulence factors (Dickey et al., 2017). It was already demonstrated that a cyclic hexapeptide, AOA-2, was able to inhibit the interaction of GNB with host cells and consequently increased the viability of these host cells (Vila-Farrés et al., 2017). At the same time, *in vivo* administration of this compound showed a large decrease in bacterial load in tissues as well as a reduction in bacteremia, together with a significant decrease in mice mortality, especially in *A. baumannii*. In this scenario, the drug discovery, such as AOA-2, should be considered as an initial stage for the development of a new class of agents with anti-virulence capacity, like other studies that are currently in preclinical stages of development, such as the compound M64 developed against the virulence factor MvfR of *P. aeruginosa* (Starkey et al., 2014) or the compound 22, a byarilmannoside, against the FimK of uropathogenic *E. coli* (Cusumano et al., 2011; Jarvis et al., 2016).

The therapeutic efficacy of these virulence factors should also be further evaluated in combination with antimicrobial agents used in the clinical setting. For example, AOA-2 has presented good therapeutic efficacy when combined with colistin in murine peritoneal sepsis model by colistin-resistant *A. baumannii* (Parra-Millán et al., 2018). Taking into account this approach as an adjuvant to antibiotic treatment, it is also necessary to optimize this peptide in order to develop a new library of compounds that improve the efficacy of the lead compound AOA-2.

A number of peptides with biological activity, particularly in the field of antibacterial research, have led to new classes of antibacterial drugs (Gordon et al., 2005). On the basis of the fact that AOA-2 has previously proved its utility as an effective source of anti-virulence compound with specific mechanism of action (Vila-Farrés et al., 2017), we have maintained this backbone as the fundamental core in our new derivatives. Three main points of variation have been considered: (1) increasing of peptide solubility by NH_2_ addition, (2) substituting tryptophan and arginine by their analogs, (3) and repositioning the general structure of AOA-2.

All these derivatives did not show toxicities in human lungs epithelial cells. Similarly, the lead compound AOA-2 did not show toxicity in these host cells (Vila-Farrés et al., 2017). This data confirm that the variation made in the AOA-2 structure did not affect the viability of host cells.

Bacterial adherence assays were employed to screen the efficacity of these derivatives. Through a various screening steps, two derivatives RW01 (solubility enhancer: NH_2_) and RW06 (analog of arginine: 2,4-Diaminobutanoic acid) have showed extended spectrum effect on the bacterial adherence to human lung epithelial cells and have been selected for the rest of the experiments. Whereas other tryptophan (3Pal: beta-(3-pyridyl)-alanine, and 2Nal: beta-(2-naphtyl)-alanine) and arginine (lysine) analogs or amino acids repositioning in the lead compound have showed short spectrum effect. This could be explained by the fact that these derivatives did not bind equally to OmpA and its homologous of the all studied GNB, due to difference in the homology of OmpA sequence. Of note, OmpA of *A. baumannii* present a 58% of homology with OmpA of *E. coli* and its homologous in *P. aeruginosa* (Gribun et al., 2003). Further structural analyses are required to decipher why RW01 and RW06 showed greater activity than AOA-2.

Interestingly, RW01 and RW06 have prevented significantly the death of human lung epithelial cells caused by *A. baumannii* and *P. aeruginosa*, and with lesser effect against *E. coli*. These results are consistent with previous observations that the lead compound AOA-2 prevents more the cell death caused by A. *baumannii* and *P. aeruginosa* than by *E. coli* (Vila-Farrés et al., 2017). The failure of both derivatives against *E. coli* may be attributable to the presence of other highly virulent factors circumventing the loss of OmpA (Nielubowicz and Mobley, 2010).

## Conclusion

The optimization program of AOA-2 has generated two derivatives with *in vitro* promising results against *P. aeruginosa* and *E. coli*. *In vivo* studies are necessary to support these *in vitro* results and to evaluate the therapeutic efficacy of both derivatives in monotherapy and in combination with clinically relevant antibiotics in severe infections by these GNB.

## Data Availability Statement

The datasets generated for this study can be found in the online repositories. The names of the repository/repositories and accession number(s) can be found in the article/Supplementary Material.

## Author Contributions

MT, JP, EG, and YS conceived the study and designed the experiments. RA-A, NB, EV, RP-M, and JS carried out the experiments. RA-A and YS analyzed the data and wrote the manuscript. MT, EG, and JP have reviewed the manuscript and the experiments. All authors read and approved the final manuscript.

## Conflict of Interest

The authors declare that the research was conducted in the absence of any commercial or financial relationships that could be construed as a potential conflict of interest.
