# Supplementary material for: AOA-2 Derivatives as Outer Membrane Protein A Inhibitors for Treatment of Gram-Negative Bacilli Infections
Source: Front Microbiol. 2021 Feb 12;12:634323. doi: 10.3389/fmicb.2021.634323 (PMC7907166; doi:10.3389/fmicb.2021.634323)
Supplement: Supplementary file 1 [file Data_Sheet_1.PDF]

ATOM	1	N	GLY	A	1	49.405	47.029	72.308	1.00	1.00	N1+
ATOM	2	CA	GLY	A	1	50.728	46.674	71.785	1.00	1.00	C
ATOM	3	C	GLY	A	1	51.204	47.670	70.729	1.00	1.00	C
ATOM	4	O	GLY	A	1	51.672	47.244	69.676	1.00	1.00	O
ATOM	5	HA2	GLY	A	1	51.450	46.671	72.601	1.00	1.00	H
ATOM	6	HA3	GLY	A	1	50.696	45.684	71.331	1.00	1.00	H
ATOM	7	H1	GLY	A	1	49.064	46.416	73.023	1.00	1.00	H
ATOM	8	H2	GLY	A	1	49.480	47.964	72.767	1.00	1.00	H
ATOM	9	H3	GLY	A	1	48.712	47.180	71.596	1.00	1.00	H
ATOM	10	N	VAL	A	2	51.053	48.974	70.977	1.00	1.00	N
ATOM	11	CA	VAL	A	2	51.508	50.045	70.105	1.00	1.00	C
ATOM	12	C	VAL	A	2	51.974	51.216	70.982	1.00	1.00	C
ATOM	13	O	VAL	A	2	51.252	51.656	71.874	1.00	1.00	O
ATOM	14	CB	VAL	A	2	50.437	50.365	69.023	1.00	1.00	C
ATOM	15	CG1	VAL	A	2	49.119	50.905	69.601	1.00	1.00	C
ATOM	16	CG2	VAL	A	2	51.001	51.372	68.018	1.00	1.00	C
ATOM	17	H	VAL	A	2	50.669	49.268	71.884	1.00	1.00	H
ATOM	18	HA	VAL	A	2	52.386	49.676	69.573	1.00	1.00	H
ATOM	19	HB	VAL	A	2	50.210	49.447	68.480	1.00	1.00	H
ATOM	20	HG11	VAL	A	2	48.400	51.058	68.797	1.00	1.00	H
ATOM	21	HG12	VAL	A	2	48.704	50.190	70.312	1.00	1.00	H
ATOM	22	HG13	VAL	A	2	49.290	51.852	70.110	1.00	1.00	H
ATOM	23	HG21	VAL	A	2	51.342	52.264	68.533	1.00	1.00	H
ATOM	24	HG22	VAL	A	2	51.850	50.938	67.500	1.00	1.00	H
ATOM	25	HG23	VAL	A	2	50.240	51.639	67.286	1.00	1.00	H
ATOM	26	N	THR	A	3	53.182	51.712	70.730	1.00	1.00	N
ATOM	27	CA	THR	A	3	53.776	52.872	71.366	1.00	1.00	C
ATOM	28	C	THR	A	3	53.655	54.023	70.347	1.00	1.00	C
ATOM	29	O	THR	A	3	54.473	54.124	69.427	1.00	1.00	O
ATOM	30	CB	THR	A	3	55.244	52.484	71.720	1.00	1.00	C
ATOM	31	CG2	THR	A	3	55.400	51.273	72.641	1.00	1.00	C
ATOM	32	OG1	THR	A	3	56.011	52.266	70.554	1.00	1.00	O
ATOM	33	H	THR	A	3	53.707	51.299	69.958	1.00	1.00	H
ATOM	34	HA	THR	A	3	53.245	53.129	72.283	1.00	1.00	H
ATOM	35	HB	THR	A	3	55.697	53.337	72.201	1.00	1.00	H
ATOM	36	HG1	THR	A	3	55.675	52.901	69.898	1.00	1.00	H
ATOM	37	HG21	THR	A	3	56.453	51.143	72.891	1.00	1.00	H
ATOM	38	HG22	THR	A	3	54.833	51.436	73.554	1.00	1.00	H
ATOM	39	HG23	THR	A	3	55.043	50.370	72.146	1.00	1.00	H
ATOM	40	N	VAL	A	4	52.602	54.848	70.465	1.00	1.00	N
ATOM	41	CA	VAL	A	4	52.330	55.973	69.571	1.00	1.00	C
ATOM	42	C	VAL	A	4	52.833	57.262	70.239	1.00	1.00	C
ATOM	43	O	VAL	A	4	52.641	57.457	71.440	1.00	1.00	O
ATOM	44	CB	VAL	A	4	50.807	56.062	69.249	1.00	1.00	C
ATOM	45	CG1	VAL	A	4	50.287	54.764	68.626	1.00	1.00	C
ATOM	46	CG2	VAL	A	4	49.881	56.398	70.425	1.00	1.00	C
ATOM	47	H	VAL	A	4	52.012	54.751	71.298	1.00	1.00	H
ATOM	48	HA	VAL	A	4	52.866	55.832	68.633	1.00	1.00	H
ATOM	49	HB	VAL	A	4	50.686	56.853	68.508	1.00	1.00	H
ATOM	50	HG11	VAL	A	4	49.299	54.930	68.196	1.00	1.00	H

ATOM 51 HG12 VAL A 4 50.972 54.433 67.848 1.00 1.00 H
ATOM 52 HG13 VAL A 4 50.209 53.998 69.396 1.00 1.00 H
ATOM 53 HG21 VAL A 4 48.848 56.448 70.079 1.00 1.00 H
ATOM 54 HG22 VAL A 4 49.951 55.627 71.191 1.00 1.00 H
ATOM 55 HG23 VAL A 4 50.141 57.365 70.852 1.00 1.00 H
ATOM 56 N THR A 5 53.473 58.152 69.478 1.00 1.00 N
ATOM 57 CA THR A 5 54.060 59.382 69.989 1.00 1.00 C
ATOM 58 C THR A 5 53.898 60.534 68.979 1.00 1.00 C
ATOM 59 O THR A 5 54.360 60.386 67.845 1.00 1.00 O
ATOM 60 CB THR A 5 55.504 59.071 70.473 1.00 1.00 C
ATOM 61 CG2 THR A 5 56.539 58.753 69.395 1.00 1.00 C
ATOM 62 OG1 THR A 5 55.962 60.177 71.216 1.00 1.00 O
ATOM 63 H THR A 5 53.664 57.913 68.503 1.00 1.00 H
ATOM 64 HA THR A 5 53.500 59.649 70.881 1.00 1.00 H
ATOM 65 HB THR A 5 55.464 58.225 71.155 1.00 1.00 H
ATOM 66 HG1 THR A 5 55.282 60.385 71.903 1.00 1.00 H
ATOM 67 HG21 THR A 5 57.490 58.528 69.872 1.00 1.00 H
ATOM 68 HG22 THR A 5 56.218 57.887 68.816 1.00 1.00 H
ATOM 69 HG23 THR A 5 56.670 59.609 68.736 1.00 1.00 H
ATOM 70 N PRO A 6 53.288 61.687 69.346 1.00 1.00 N
ATOM 71 CA PRO A 6 53.521 62.949 68.637 1.00 1.00 C
ATOM 72 C PRO A 6 54.915 63.473 69.028 1.00 1.00 C
ATOM 73 O PRO A 6 55.288 63.450 70.205 1.00 1.00 O
ATOM 74 CB PRO A 6 52.398 63.885 69.099 1.00 1.00 C
ATOM 75 CG PRO A 6 52.082 63.393 70.513 1.00 1.00 C
ATOM 76 CD PRO A 6 52.358 61.890 70.451 1.00 1.00 C
ATOM 77 HA PRO A 6 53.463 62.812 67.557 1.00 1.00 H
ATOM 78 HB2 PRO A 6 52.704 64.932 69.091 1.00 1.00 H
ATOM 79 HB3 PRO A 6 51.524 63.740 68.462 1.00 1.00 H
ATOM 80 HG2 PRO A 6 52.764 63.860 71.225 1.00 1.00 H
ATOM 81 HG3 PRO A 6 51.048 63.600 70.789 1.00 1.00 H
ATOM 82 HD2 PRO A 6 52.773 61.561 71.398 1.00 1.00 H
ATOM 83 HD3 PRO A 6 51.432 61.354 70.243 1.00 1.00 H
ATOM 84 N LEU A 7 55.685 63.902 68.031 1.00 1.00 N
ATOM 85 CA LEU A 7 57.073 64.302 68.172 1.00 1.00 C
ATOM 86 C LEU A 7 57.115 65.833 68.174 1.00 1.00 C
ATOM 87 O LEU A 7 56.606 66.478 67.256 1.00 1.00 O
ATOM 88 CB LEU A 7 57.850 63.783 66.940 1.00 1.00 C
ATOM 89 CG LEU A 7 57.778 62.257 66.733 1.00 1.00 C
ATOM 90 CD1 LEU A 7 58.101 61.944 65.273 1.00 1.00 C
ATOM 91 CD2 LEU A 7 58.747 61.502 67.652 1.00 1.00 C
ATOM 92 H LEU A 7 55.271 63.986 67.104 1.00 1.00 H
ATOM 93 HA LEU A 7 57.510 63.901 69.087 1.00 1.00 H
ATOM 94 HB2 LEU A 7 57.439 64.271 66.055 1.00 1.00 H
ATOM 95 HB3 LEU A 7 58.892 64.086 67.033 1.00 1.00 H
ATOM 96 HG LEU A 7 56.770 61.888 66.917 1.00 1.00 H
ATOM 97 HD11 LEU A 7 57.970 60.882 65.099 1.00 1.00 H
ATOM 98 HD12 LEU A 7 57.435 62.488 64.606 1.00 1.00 H
ATOM 99 HD13 LEU A 7 59.127 62.216 65.036 1.00 1.00 H
ATOM 100 HD21 LEU A 7 58.727 60.440 67.408 1.00 1.00 H

```
ATOM    101 HD22 LEU A   7      59.759  61.881  67.510  1.00  1.00           H
ATOM    102 HD23 LEU A   7      58.447  61.634  68.692  1.00  1.00           H
ATOM    103  N   LEU A   8      57.744  66.417  69.193  1.00  1.00           N
ATOM    104  CA  LEU A   8      58.061  67.831  69.276  1.00  1.00           C
ATOM    105  C   LEU A   8      59.406  68.004  68.558  1.00  1.00           C
ATOM    106  O   LEU A   8      60.465  67.674  69.098  1.00  1.00           O
ATOM    107  CB  LEU A   8      58.216  68.240  70.762  1.00  1.00           C
ATOM    108  CG  LEU A   8      56.991  67.999  71.669  1.00  1.00           C
ATOM    109  CD1 LEU A   8      57.338  68.435  73.095  1.00  1.00           C
ATOM    110  CD2 LEU A   8      55.745  68.765  71.215  1.00  1.00           C
ATOM    111  H   LEU A   8      58.121  65.818  69.927  1.00  1.00           H
ATOM    112  HA  LEU A   8      57.289  68.436  68.798  1.00  1.00           H
ATOM    113  HB2 LEU A   8      59.054  67.696  71.191  1.00  1.00           H
ATOM    114  HB3 LEU A   8      58.467  69.301  70.793  1.00  1.00           H
ATOM    115  HG  LEU A   8      56.755  66.935  71.689  1.00  1.00           H
ATOM    116 HD11 LEU A   8      56.483  68.263  73.749  1.00  1.00           H
ATOM    117 HD12 LEU A   8      58.179  67.847  73.462  1.00  1.00           H
ATOM    118 HD13 LEU A   8      57.601  69.493  73.109  1.00  1.00           H
ATOM    119 HD21 LEU A   8      55.954  69.835  71.177  1.00  1.00           H
ATOM    120 HD22 LEU A   8      55.443  68.419  70.227  1.00  1.00           H
ATOM    121 HD23 LEU A   8      54.927  68.582  71.912  1.00  1.00           H
ATOM    122  N   LEU A   9      59.332  68.481  67.316  1.00  1.00           N
ATOM    123  CA  LEU A   9      60.436  69.032  66.546  1.00  1.00           C
ATOM    124  C   LEU A   9      60.347  70.541  66.778  1.00  1.00           C
ATOM    125  O   LEU A   9      59.297  71.112  66.500  1.00  1.00           O
ATOM    126  CB  LEU A   9      60.180  68.817  65.031  1.00  1.00           C
ATOM    127  CG  LEU A   9      60.313  67.373  64.513  1.00  1.00           C
ATOM    128  CD1 LEU A   9      59.074  66.536  64.828  1.00  1.00           C
ATOM    129  CD2 LEU A   9      60.491  67.379  62.993  1.00  1.00           C
ATOM    130  H   LEU A   9      58.410  68.590  66.913  1.00  1.00           H
ATOM    131  HA  LEU A   9      61.403  68.630  66.855  1.00  1.00           H
ATOM    132  HB2 LEU A   9      59.191  69.194  64.763  1.00  1.00           H
ATOM    133  HB3 LEU A   9      60.902  69.431  64.494  1.00  1.00           H
ATOM    134  HG  LEU A   9      61.191  66.910  64.957  1.00  1.00           H
ATOM    135 HD11 LEU A   9      59.109  65.594  64.283  1.00  1.00           H
ATOM    136 HD12 LEU A   9      59.038  66.320  65.892  1.00  1.00           H
ATOM    137 HD13 LEU A   9      58.168  67.067  64.540  1.00  1.00           H
ATOM    138 HD21 LEU A   9      60.617  66.357  62.636  1.00  1.00           H
ATOM    139 HD22 LEU A   9      59.619  67.821  62.511  1.00  1.00           H
ATOM    140 HD23 LEU A   9      61.381  67.951  62.729  1.00  1.00           H
ATOM    141  N   GLY A  10      61.411  71.164  67.281  1.00  1.00           N
ATOM    142  CA  GLY A  10      61.623  72.607  67.303  1.00  1.00           C
ATOM    143  C   GLY A  10      63.005  72.811  66.695  1.00  1.00           C
ATOM    144  O   GLY A  10      63.935  72.109  67.100  1.00  1.00           O
ATOM    145  H   GLY A  10      62.213  70.600  67.535  1.00  1.00           H
ATOM    146  HA2 GLY A  10      60.871  73.124  66.706  1.00  1.00           H
ATOM    147  HA3 GLY A  10      61.610  72.993  68.322  1.00  1.00           H
ATOM    148  N   TYR A  11      63.133  73.674  65.687  1.00  1.00           N
ATOM    149  CA  TYR A  11      64.352  73.853  64.902  1.00  1.00           C
ATOM    150  C   TYR A  11      64.615  75.345  64.710  1.00  1.00           C
```

ATOM 151 O TYR A 11 63.838 76.018 64.038 1.00 1.00 O
ATOM 152 CB TYR A 11 64.267 73.067 63.569 1.00 1.00 C
ATOM 153 CG TYR A 11 64.537 71.583 63.748 1.00 1.00 C
ATOM 154 CD1 TYR A 11 63.536 70.739 64.267 1.00 1.00 C
ATOM 155 CD2 TYR A 11 65.812 71.055 63.466 1.00 1.00 C
ATOM 156 CE1 TYR A 11 63.833 69.399 64.576 1.00 1.00 C
ATOM 157 CE2 TYR A 11 66.093 69.703 63.736 1.00 1.00 C
ATOM 158 CZ TYR A 11 65.112 68.886 64.317 1.00 1.00 C
ATOM 159 OH TYR A 11 65.414 67.604 64.651 1.00 1.00 O
ATOM 160 H TYR A 11 62.317 74.212 65.415 1.00 1.00 H
ATOM 161 HA TYR A 11 65.199 73.446 65.459 1.00 1.00 H
ATOM 162 HB2 TYR A 11 63.287 73.214 63.113 1.00 1.00 H
ATOM 163 HB3 TYR A 11 65.011 73.468 62.882 1.00 1.00 H
ATOM 164 HD1 TYR A 11 62.552 71.135 64.466 1.00 1.00 H
ATOM 165 HD2 TYR A 11 66.585 71.689 63.056 1.00 1.00 H
ATOM 166 HE1 TYR A 11 63.088 68.766 65.026 1.00 1.00 H
ATOM 167 HE2 TYR A 11 67.072 69.298 63.529 1.00 1.00 H
ATOM 168 HH TYR A 11 64.979 67.331 65.487 1.00 1.00 H
ATOM 169 N THR A 12 65.716 75.849 65.279 1.00 1.00 N
ATOM 170 CA THR A 12 66.127 77.255 65.356 1.00 1.00 C
ATOM 171 C THR A 12 66.468 77.855 63.978 1.00 1.00 C
ATOM 172 O THR A 12 66.465 79.072 63.803 1.00 1.00 O
ATOM 173 CB THR A 12 67.376 77.305 66.282 1.00 1.00 C
ATOM 174 CG2 THR A 12 67.761 78.709 66.750 1.00 1.00 C
ATOM 175 OG1 THR A 12 67.153 76.488 67.418 1.00 1.00 O
ATOM 176 H THR A 12 66.243 75.229 65.881 1.00 1.00 H
ATOM 177 HA THR A 12 65.326 77.840 65.803 1.00 1.00 H
ATOM 178 HB THR A 12 68.223 76.882 65.741 1.00 1.00 H
ATOM 179 HG1 THR A 12 66.242 76.725 67.798 1.00 1.00 H
ATOM 180 HG21 THR A 12 68.574 78.642 67.472 1.00 1.00 H
ATOM 181 HG22 THR A 12 68.093 79.306 65.901 1.00 1.00 H
ATOM 182 HG23 THR A 12 66.904 79.195 67.217 1.00 1.00 H
ATOM 183 N PHE A 13 66.734 76.987 63.000 1.00 1.00 N
ATOM 184 CA PHE A 13 66.972 77.280 61.603 1.00 1.00 C
ATOM 185 C PHE A 13 66.208 76.214 60.815 1.00 1.00 C
ATOM 186 O PHE A 13 66.267 75.036 61.177 1.00 1.00 O
ATOM 187 CB PHE A 13 68.486 77.215 61.293 1.00 1.00 C
ATOM 188 CG PHE A 13 69.327 78.216 62.065 1.00 1.00 C
ATOM 189 CD1 PHE A 13 69.445 79.539 61.601 1.00 1.00 C
ATOM 190 CD2 PHE A 13 69.980 77.834 63.253 1.00 1.00 C
ATOM 191 CE1 PHE A 13 70.212 80.475 62.316 1.00 1.00 C
ATOM 192 CE2 PHE A 13 70.743 78.772 63.972 1.00 1.00 C
ATOM 193 CZ PHE A 13 70.861 80.092 63.502 1.00 1.00 C
ATOM 194 H PHE A 13 66.712 76.006 63.238 1.00 1.00 H
ATOM 195 HA PHE A 13 66.588 78.271 61.354 1.00 1.00 H
ATOM 196 HB2 PHE A 13 68.850 76.209 61.507 1.00 1.00 H
ATOM 197 HB3 PHE A 13 68.628 77.393 60.226 1.00 1.00 H
ATOM 198 HD1 PHE A 13 68.933 79.847 60.702 1.00 1.00 H
ATOM 199 HD2 PHE A 13 69.891 76.823 63.622 1.00 1.00 H
ATOM 200 HE1 PHE A 13 70.282 81.494 61.961 1.00 1.00 H

```
ATOM    201  HE2 PHE A  13      71.232  78.479  64.889  1.00  1.00           H
ATOM    202  HZ  PHE A  13      71.440  80.816  64.056  1.00  1.00           H
ATOM    203  N   GLN A  14      65.560  76.600  59.717  1.00  1.00           N
ATOM    204  CA  GLN A  14      65.097  75.705  58.665  1.00  1.00           C
ATOM    205  C   GLN A  14      65.224  76.440  57.323  1.00  1.00           C
ATOM    206  O   GLN A  14      65.733  77.564  57.285  1.00  1.00           O
ATOM    207  CB  GLN A  14      63.708  75.089  58.973  1.00  1.00           C
ATOM    208  CG  GLN A  14      63.881  73.564  59.076  1.00  1.00           C
ATOM    209  CD  GLN A  14      62.586  72.794  58.958  1.00  1.00           C
ATOM    210  NE2 GLN A  14      62.055  72.660  57.760  1.00  1.00           N
ATOM    211  OE1 GLN A  14      62.066  72.267  59.925  1.00  1.00           O
ATOM    212  H   GLN A  14      65.582  77.582  59.472  1.00  1.00           H
ATOM    213  HA  GLN A  14      65.827  74.896  58.610  1.00  1.00           H
ATOM    214  HB2 GLN A  14      63.306  75.474  59.912  1.00  1.00           H
ATOM    215  HB3 GLN A  14      63.008  75.329  58.173  1.00  1.00           H
ATOM    216  HG2 GLN A  14      64.528  73.207  58.276  1.00  1.00           H
ATOM    217  HG3 GLN A  14      64.346  73.323  60.032  1.00  1.00           H
ATOM    218 HE21 GLN A  14      62.549  73.023  56.934  1.00  1.00           H
ATOM    219 HE22 GLN A  14      61.203  72.137  57.672  1.00  1.00           H
ATOM    220  N   ASP A  15      64.822  75.774  56.238  1.00  1.00           N
ATOM    221  CA  ASP A  15      64.892  76.127  54.817  1.00  1.00           C
ATOM    222  C   ASP A  15      64.649  77.618  54.521  1.00  1.00           C
ATOM    223  O   ASP A  15      63.502  78.069  54.510  1.00  1.00           O
ATOM    224  CB  ASP A  15      63.981  75.213  53.941  1.00  1.00           C
ATOM    225  CG  ASP A  15      63.659  73.766  54.405  1.00  1.00           C
ATOM    226  OD1 ASP A  15      63.492  72.894  53.518  1.00  1.00           O
ATOM    227  OD2 ASP A  15      63.537  73.526  55.633  1.00  1.00           O1-
ATOM    228  H   ASP A  15      64.396  74.860  56.398  1.00  1.00           H
ATOM    229  HA  ASP A  15      65.915  75.906  54.511  1.00  1.00           H
ATOM    230  HB2 ASP A  15      63.012  75.700  53.831  1.00  1.00           H
ATOM    231  HB3 ASP A  15      64.420  75.174  52.942  1.00  1.00           H
ATOM    232  N   SER A  16      65.720  78.388  54.273  1.00  1.00           N
ATOM    233  CA  SER A  16      65.643  79.814  53.967  1.00  1.00           C
ATOM    234  C   SER A  16      64.917  80.007  52.628  1.00  1.00           C
ATOM    235  O   SER A  16      65.296  79.426  51.610  1.00  1.00           O
ATOM    236  CB  SER A  16      67.057  80.436  53.922  1.00  1.00           C
ATOM    237  OG  SER A  16      66.905  81.827  53.687  1.00  1.00           O
ATOM    238  H   SER A  16      66.626  77.957  54.359  1.00  1.00           H
ATOM    239  HA  SER A  16      65.071  80.284  54.765  1.00  1.00           H
ATOM    240  HB2 SER A  16      67.559  80.281  54.878  1.00  1.00           H
ATOM    241  HB3 SER A  16      67.642  79.986  53.119  1.00  1.00           H
ATOM    242  HG  SER A  16      66.285  82.182  54.373  1.00  1.00           H
ATOM    243  N   GLN A  17      63.839  80.796  52.645  1.00  1.00           N
ATOM    244  CA  GLN A  17      62.907  80.914  51.527  1.00  1.00           C
ATOM    245  C   GLN A  17      63.379  81.981  50.511  1.00  1.00           C
ATOM    246  O   GLN A  17      62.838  82.078  49.405  1.00  1.00           O
ATOM    247  CB  GLN A  17      61.530  81.300  52.126  1.00  1.00           C
ATOM    248  CG  GLN A  17      60.334  81.143  51.176  1.00  1.00           C
ATOM    249  CD  GLN A  17      60.001  79.685  50.929  1.00  1.00           C
ATOM    250  NE2 GLN A  17      60.513  79.093  49.864  1.00  1.00           N
```

```
ATOM    251  OE1 GLN A  17      59.279  79.070  51.695  1.00  1.00           O
ATOM    252  H   GLN A  17      63.610  81.246  53.524  1.00  1.00           H
ATOM    253  HA  GLN A  17      62.831  79.949  51.025  1.00  1.00           H
ATOM    254  HB2 GLN A  17      61.339  80.684  53.007  1.00  1.00           H
ATOM    255  HB3 GLN A  17      61.576  82.339  52.458  1.00  1.00           H
ATOM    256  HG2 GLN A  17      59.463  81.596  51.650  1.00  1.00           H
ATOM    257  HG3 GLN A  17      60.508  81.655  50.233  1.00  1.00           H
ATOM    258 HE21 GLN A  17      60.294  78.117  49.743  1.00  1.00           H
ATOM    259 HE22 GLN A  17      61.117  79.590  49.232  1.00  1.00           H
ATOM    260  N   HIE A  18      64.403  82.775  50.861  1.00  1.00           N
ATOM    261  CA  HIE A  18      64.977  83.822  50.019  1.00  1.00           C
ATOM    262  C   HIE A  18      65.880  83.179  48.953  1.00  1.00           C
ATOM    263  O   HIE A  18      67.096  83.067  49.111  1.00  1.00           O
ATOM    264  CB  HIE A  18      65.784  84.802  50.894  1.00  1.00           C
ATOM    265  CG  HIE A  18      66.332  86.008  50.176  1.00  1.00           C
ATOM    266  CD2 HIE A  18      67.516  86.631  50.448  1.00  1.00           C
ATOM    267  ND1 HIE A  18      65.672  86.712  49.172  1.00  1.00           N
ATOM    268  CE1 HIE A  18      66.462  87.755  48.874  1.00  1.00           C
ATOM    269  NE2 HIE A  18      67.578  87.729  49.624  1.00  1.00           N
ATOM    270  H   HIE A  18      64.822  82.613  51.764  1.00  1.00           H
ATOM    271  HA  HIE A  18      64.170  84.371  49.530  1.00  1.00           H
ATOM    272  HB2 HIE A  18      65.135  85.163  51.693  1.00  1.00           H
ATOM    273  HB3 HIE A  18      66.609  84.253  51.347  1.00  1.00           H
ATOM    274  HD2 HIE A  18      68.251  86.327  51.174  1.00  1.00           H
ATOM    275  HE1 HIE A  18      66.234  88.506  48.132  1.00  1.00           H
ATOM    276  HE2 HIE A  18      68.331  88.401  49.589  1.00  1.00           H
ATOM    277  N   ASN A  19      65.249  82.757  47.853  1.00  1.00           N
ATOM    278  CA  ASN A  19      65.766  82.213  46.583  1.00  1.00           C
ATOM    279  C   ASN A  19      66.750  83.147  45.835  1.00  1.00           C
ATOM    280  O   ASN A  19      66.602  83.466  44.653  1.00  1.00           O
ATOM    281  CB  ASN A  19      64.597  81.690  45.701  1.00  1.00           C
ATOM    282  CG  ASN A  19      63.358  82.580  45.620  1.00  1.00           C
ATOM    283  ND2 ASN A  19      62.467  82.549  46.597  1.00  1.00           N
ATOM    284  OD1 ASN A  19      63.148  83.291  44.652  1.00  1.00           O
ATOM    285  H   ASN A  19      64.244  82.852  47.913  1.00  1.00           H
ATOM    286  HA  ASN A  19      66.357  81.335  46.849  1.00  1.00           H
ATOM    287  HB2 ASN A  19      64.955  81.489  44.691  1.00  1.00           H
ATOM    288  HB3 ASN A  19      64.279  80.735  46.118  1.00  1.00           H
ATOM    289 HD21 ASN A  19      62.588  81.977  47.426  1.00  1.00           H
ATOM    290 HD22 ASN A  19      61.638  83.107  46.482  1.00  1.00           H
ATOM    291  N   ASN A  20      67.750  83.600  46.582  1.00  1.00           N
ATOM    292  CA  ASN A  20      68.880  84.452  46.243  1.00  1.00           C
ATOM    293  C   ASN A  20      70.131  83.588  46.020  1.00  1.00           C
ATOM    294  O   ASN A  20      70.973  83.946  45.207  1.00  1.00           O
ATOM    295  CB  ASN A  20      69.103  85.317  47.500  1.00  1.00           C
ATOM    296  CG  ASN A  20      70.263  86.288  47.405  1.00  1.00           C
ATOM    297  ND2 ASN A  20      71.131  86.280  48.402  1.00  1.00           N
ATOM    298  OD1 ASN A  20      70.364  87.071  46.472  1.00  1.00           O
ATOM    299  H   ASN A  20      67.690  83.343  47.561  1.00  1.00           H
ATOM    300  HA  ASN A  20      68.671  85.070  45.369  1.00  1.00           H
```

ATOM 301 HB2 ASN A 20 68.205 85.905 47.667 1.00 1.00 H
ATOM 302 HB3 ASN A 20 69.245 84.677 48.372 1.00 1.00 H
ATOM 303 HD21 ASN A 20 71.037 85.629 49.185 1.00 1.00 H
ATOM 304 HD22 ASN A 20 71.910 86.913 48.342 1.00 1.00 H
ATOM 305 N GLY A 21 70.241 82.455 46.728 1.00 1.00 N
ATOM 306 CA GLY A 21 71.395 81.556 46.691 1.00 1.00 C
ATOM 307 C GLY A 21 72.071 81.395 48.055 1.00 1.00 C
ATOM 308 O GLY A 21 72.998 80.602 48.171 1.00 1.00 O
ATOM 309 H GLY A 21 69.469 82.201 47.324 1.00 1.00 H
ATOM 310 HA2 GLY A 21 71.069 80.573 46.351 1.00 1.00 H
ATOM 311 HA3 GLY A 21 72.143 81.929 45.991 1.00 1.00 H
ATOM 312 N GLY A 22 71.598 82.099 49.090 1.00 1.00 N
ATOM 313 CA GLY A 22 72.264 82.245 50.377 1.00 1.00 C
ATOM 314 C GLY A 22 71.967 83.630 50.957 1.00 1.00 C
ATOM 315 O GLY A 22 71.098 84.349 50.443 1.00 1.00 O
ATOM 316 H GLY A 22 70.815 82.714 48.922 1.00 1.00 H
ATOM 317 HA2 GLY A 22 71.912 81.481 51.070 1.00 1.00 H
ATOM 318 HA3 GLY A 22 73.344 82.139 50.256 1.00 1.00 H
ATOM 319 N LYS A 23 72.644 83.968 52.054 1.00 1.00 N
ATOM 320 CA LYS A 23 72.444 85.120 52.940 1.00 1.00 C
ATOM 321 C LYS A 23 73.670 85.280 53.840 1.00 1.00 C
ATOM 322 O LYS A 23 74.109 86.391 54.124 1.00 1.00 O
ATOM 323 CB LYS A 23 71.218 84.955 53.867 1.00 1.00 C
ATOM 324 CG LYS A 23 69.829 85.123 53.244 1.00 1.00 C
ATOM 325 CD LYS A 23 68.793 85.138 54.378 1.00 1.00 C
ATOM 326 CE LYS A 23 67.358 85.235 53.863 1.00 1.00 C
ATOM 327 NZ LYS A 23 66.399 85.269 54.977 1.00 1.00 N1+
ATOM 328 H LYS A 23 73.508 83.427 52.199 1.00 1.00 H
ATOM 329 HA LYS A 23 72.330 86.026 52.344 1.00 1.00 H
ATOM 330 HB2 LYS A 23 71.270 83.976 54.347 1.00 1.00 H
ATOM 331 HB3 LYS A 23 71.310 85.707 54.654 1.00 1.00 H
ATOM 332 HG2 LYS A 23 69.797 86.067 52.702 1.00 1.00 H
ATOM 333 HG3 LYS A 23 69.618 84.293 52.568 1.00 1.00 H
ATOM 334 HD2 LYS A 23 68.892 84.219 54.959 1.00 1.00 H
ATOM 335 HD3 LYS A 23 68.993 85.991 55.027 1.00 1.00 H
ATOM 336 HE2 LYS A 23 67.249 86.135 53.257 1.00 1.00 H
ATOM 337 HE3 LYS A 23 67.155 84.356 53.252 1.00 1.00 H
ATOM 338 HZ1 LYS A 23 65.446 85.250 54.648 1.00 1.00 H
ATOM 339 HZ2 LYS A 23 66.541 84.430 55.544 1.00 1.00 H
ATOM 340 HZ3 LYS A 23 66.562 86.080 55.572 1.00 1.00 H
ATOM 341 N ASP A 24 74.163 84.140 54.338 1.00 1.00 N
ATOM 342 CA ASP A 24 75.554 83.885 54.723 1.00 1.00 C
ATOM 343 C ASP A 24 75.898 84.353 56.144 1.00 1.00 C
ATOM 344 O ASP A 24 76.821 83.836 56.768 1.00 1.00 O
ATOM 345 CB ASP A 24 76.580 84.238 53.613 1.00 1.00 C
ATOM 346 CG ASP A 24 76.314 83.555 52.248 1.00 1.00 C
ATOM 347 OD1 ASP A 24 75.232 82.939 52.066 1.00 1.00 O
ATOM 348 OD2 ASP A 24 77.211 83.668 51.386 1.00 1.00 O1-
ATOM 349 H ASP A 24 73.611 83.310 54.182 1.00 1.00 H
ATOM 350 HA ASP A 24 75.617 82.798 54.798 1.00 1.00 H

```
ATOM    351  HB2 ASP A  24      76.590  85.319  53.471  1.00  1.00           H
ATOM    352  HB3 ASP A  24      77.571  83.941  53.959  1.00  1.00           H
ATOM    353  N   GLY A  25      75.097  85.268  56.698  1.00  1.00           N
ATOM    354  CA  GLY A  25      75.136  85.718  58.079  1.00  1.00           C
ATOM    355  C   GLY A  25      75.448  87.208  58.116  1.00  1.00           C
ATOM    356  O   GLY A  25      74.831  87.988  57.390  1.00  1.00           O
ATOM    357  H   GLY A  25      74.450  85.723  56.063  1.00  1.00           H
ATOM    358  HA2 GLY A  25      74.167  85.553  58.548  1.00  1.00           H
ATOM    359  HA3 GLY A  25      75.890  85.165  58.640  1.00  1.00           H
ATOM    360  N   ASN A  26      76.397  87.604  58.976  1.00  1.00           N
ATOM    361  CA  ASN A  26      77.043  88.921  59.053  1.00  1.00           C
ATOM    362  C   ASN A  26      76.137  89.995  59.682  1.00  1.00           C
ATOM    363  O   ASN A  26      76.494  90.597  60.692  1.00  1.00           O
ATOM    364  CB  ASN A  26      77.655  89.332  57.689  1.00  1.00           C
ATOM    365  CG  ASN A  26      78.342  90.691  57.719  1.00  1.00           C
ATOM    366  ND2 ASN A  26      77.588  91.778  57.653  1.00  1.00           N
ATOM    367  OD1 ASN A  26      79.557  90.780  57.792  1.00  1.00           O
ATOM    368  H   ASN A  26      76.823  86.879  59.534  1.00  1.00           H
ATOM    369  HA  ASN A  26      77.885  88.808  59.738  1.00  1.00           H
ATOM    370  HB2 ASN A  26      78.391  88.584  57.394  1.00  1.00           H
ATOM    371  HB3 ASN A  26      76.890  89.361  56.917  1.00  1.00           H
ATOM    372 HD21 ASN A  26      76.584  91.715  57.611  1.00  1.00           H
ATOM    373 HD22 ASN A  26      78.069  92.662  57.652  1.00  1.00           H
ATOM    374  N   LEU A  27      74.965  90.235  59.089  1.00  1.00           N
ATOM    375  CA  LEU A  27      73.995  91.224  59.536  1.00  1.00           C
ATOM    376  C   LEU A  27      73.100  90.587  60.610  1.00  1.00           C
ATOM    377  O   LEU A  27      72.554  89.499  60.416  1.00  1.00           O
ATOM    378  CB  LEU A  27      73.098  91.644  58.348  1.00  1.00           C
ATOM    379  CG  LEU A  27      73.813  92.225  57.110  1.00  1.00           C
ATOM    380  CD1 LEU A  27      74.151  91.160  56.058  1.00  1.00           C
ATOM    381  CD2 LEU A  27      72.915  93.257  56.422  1.00  1.00           C
ATOM    382  H   LEU A  27      74.699  89.614  58.326  1.00  1.00           H
ATOM    383  HA  LEU A  27      74.506  92.101  59.938  1.00  1.00           H
ATOM    384  HB2 LEU A  27      72.495  90.792  58.031  1.00  1.00           H
ATOM    385  HB3 LEU A  27      72.407  92.395  58.735  1.00  1.00           H
ATOM    386  HG  LEU A  27      74.731  92.724  57.421  1.00  1.00           H
ATOM    387 HD11 LEU A  27      74.843  90.426  56.457  1.00  1.00           H
ATOM    388 HD12 LEU A  27      73.244  90.646  55.736  1.00  1.00           H
ATOM    389 HD13 LEU A  27      74.618  91.630  55.193  1.00  1.00           H
ATOM    390 HD21 LEU A  27      73.422  93.673  55.552  1.00  1.00           H
ATOM    391 HD22 LEU A  27      71.981  92.790  56.109  1.00  1.00           H
ATOM    392 HD23 LEU A  27      72.690  94.069  57.114  1.00  1.00           H
ATOM    393  N   THR A  28      72.904  91.283  61.734  1.00  1.00           N
ATOM    394  CA  THR A  28      72.095  90.868  62.878  1.00  1.00           C
ATOM    395  C   THR A  28      70.592  91.157  62.626  1.00  1.00           C
ATOM    396  O   THR A  28      69.912  91.773  63.449  1.00  1.00           O
ATOM    397  CB  THR A  28      72.655  91.638  64.103  1.00  1.00           C
ATOM    398  CG2 THR A  28      74.006  91.083  64.562  1.00  1.00           C
ATOM    399  OG1 THR A  28      72.836  93.007  63.798  1.00  1.00           O
ATOM    400  H   THR A  28      73.365  92.177  61.836  1.00  1.00           H
```

ATOM 401 HA THR A 28 72.216 89.796 63.041 1.00 1.00 H
ATOM 402 HB THR A 28 71.965 91.554 64.940 1.00 1.00 H
ATOM 403 HG1 THR A 28 72.956 93.467 64.632 1.00 1.00 H
ATOM 404 HG21 THR A 28 74.340 91.618 65.451 1.00 1.00 H
ATOM 405 HG22 THR A 28 73.900 90.026 64.808 1.00 1.00 H
ATOM 406 HG23 THR A 28 74.752 91.193 63.774 1.00 1.00 H
ATOM 407 N ASN A 29 70.107 90.762 61.446 1.00 1.00 N
ATOM 408 CA ASN A 29 68.759 90.820 60.878 1.00 1.00 C
ATOM 409 C ASN A 29 68.945 90.537 59.383 1.00 1.00 C
ATOM 410 O ASN A 29 69.947 90.944 58.798 1.00 1.00 O
ATOM 411 CB ASN A 29 67.990 92.156 61.092 1.00 1.00 C
ATOM 412 CG ASN A 29 68.759 93.394 60.662 1.00 1.00 C
ATOM 413 ND2 ASN A 29 69.599 93.923 61.534 1.00 1.00 N
ATOM 414 OD1 ASN A 29 68.590 93.888 59.558 1.00 1.00 O
ATOM 415 H ASN A 29 70.796 90.320 60.841 1.00 1.00 H
ATOM 416 HA ASN A 29 68.169 90.013 61.315 1.00 1.00 H
ATOM 417 HB2 ASN A 29 67.056 92.126 60.532 1.00 1.00 H
ATOM 418 HB3 ASN A 29 67.733 92.254 62.146 1.00 1.00 H
ATOM 419 HD21 ASN A 29 69.735 93.454 62.425 1.00 1.00 H
ATOM 420 HD22 ASN A 29 70.130 94.727 61.251 1.00 1.00 H
ATOM 421 N GLY A 30 68.042 89.766 58.774 1.00 1.00 N
ATOM 422 CA GLY A 30 68.227 89.241 57.422 1.00 1.00 C
ATOM 423 C GLY A 30 68.098 87.716 57.400 1.00 1.00 C
ATOM 424 O GLY A 30 67.144 87.230 56.783 1.00 1.00 O
ATOM 425 H GLY A 30 67.230 89.462 59.294 1.00 1.00 H
ATOM 426 HA2 GLY A 30 67.465 89.664 56.767 1.00 1.00 H
ATOM 427 HA3 GLY A 30 69.206 89.509 57.025 1.00 1.00 H
ATOM 428 N PRO A 31 69.010 86.943 58.035 1.00 1.00 N
ATOM 429 CA PRO A 31 68.874 85.487 58.142 1.00 1.00 C
ATOM 430 C PRO A 31 67.785 85.085 59.153 1.00 1.00 C
ATOM 431 O PRO A 31 67.508 85.807 60.111 1.00 1.00 O
ATOM 432 CB PRO A 31 70.264 84.987 58.563 1.00 1.00 C
ATOM 433 CG PRO A 31 70.826 86.155 59.372 1.00 1.00 C
ATOM 434 CD PRO A 31 70.245 87.384 58.673 1.00 1.00 C
ATOM 435 HA PRO A 31 68.623 85.056 57.175 1.00 1.00 H
ATOM 436 HB2 PRO A 31 70.215 84.071 59.154 1.00 1.00 H
ATOM 437 HB3 PRO A 31 70.878 84.833 57.674 1.00 1.00 H
ATOM 438 HG2 PRO A 31 70.450 86.105 60.395 1.00 1.00 H
ATOM 439 HG3 PRO A 31 71.917 86.166 59.366 1.00 1.00 H
ATOM 440 HD2 PRO A 31 70.065 88.160 59.413 1.00 1.00 H
ATOM 441 HD3 PRO A 31 70.942 87.741 57.913 1.00 1.00 H
ATOM 442 N GLU A 32 67.165 83.935 58.886 1.00 1.00 N
ATOM 443 CA GLU A 32 66.100 83.282 59.629 1.00 1.00 C
ATOM 444 C GLU A 32 66.626 82.811 60.995 1.00 1.00 C
ATOM 445 O GLU A 32 67.648 82.128 61.065 1.00 1.00 O
ATOM 446 CB GLU A 32 65.646 82.035 58.822 1.00 1.00 C
ATOM 447 CG GLU A 32 64.759 82.289 57.590 1.00 1.00 C
ATOM 448 CD GLU A 32 65.530 82.651 56.326 1.00 1.00 C
ATOM 449 OE1 GLU A 32 66.729 82.990 56.398 1.00 1.00 O
ATOM 450 OE2 GLU A 32 64.960 82.651 55.213 1.00 1.00 O1-

```
ATOM    451  H   GLU A  32      67.397  83.485  57.998  1.00  1.00           H
ATOM    452  HA  GLU A  32      65.257  83.959  59.767  1.00  1.00           H
ATOM    453  HB2 GLU A  32      66.518  81.450  58.523  1.00  1.00           H
ATOM    454  HB3 GLU A  32      65.085  81.395  59.499  1.00  1.00           H
ATOM    455  HG2 GLU A  32      64.204  81.374  57.381  1.00  1.00           H
ATOM    456  HG3 GLU A  32      64.040  83.075  57.814  1.00  1.00           H
ATOM    457  N   LEU A  33      65.899  83.158  62.058  1.00  1.00           N
ATOM    458  CA  LEU A  33      65.884  82.470  63.341  1.00  1.00           C
ATOM    459  C   LEU A  33      64.428  82.015  63.484  1.00  1.00           C
ATOM    460  O   LEU A  33      63.529  82.791  63.148  1.00  1.00           O
ATOM    461  CB  LEU A  33      66.245  83.426  64.506  1.00  1.00           C
ATOM    462  CG  LEU A  33      67.724  83.797  64.737  1.00  1.00           C
ATOM    463  CD1 LEU A  33      68.569  82.567  65.070  1.00  1.00           C
ATOM    464  CD2 LEU A  33      68.382  84.564  63.586  1.00  1.00           C
ATOM    465  H   LEU A  33      65.105  83.761  61.893  1.00  1.00           H
ATOM    466  HA  LEU A  33      66.544  81.603  63.332  1.00  1.00           H
ATOM    467  HB2 LEU A  33      65.678  84.350  64.384  1.00  1.00           H
ATOM    468  HB3 LEU A  33      65.890  82.964  65.429  1.00  1.00           H
ATOM    469  HG  LEU A  33      67.752  84.452  65.608  1.00  1.00           H
ATOM    470 HD11 LEU A  33      68.619  81.902  64.209  1.00  1.00           H
ATOM    471 HD12 LEU A  33      69.577  82.877  65.342  1.00  1.00           H
ATOM    472 HD13 LEU A  33      68.123  82.029  65.907  1.00  1.00           H
ATOM    473 HD21 LEU A  33      68.529  83.903  62.734  1.00  1.00           H
ATOM    474 HD22 LEU A  33      67.752  85.403  63.291  1.00  1.00           H
ATOM    475 HD23 LEU A  33      69.351  84.941  63.912  1.00  1.00           H
ATOM    476  N   GLN A  34      64.213  80.776  63.928  1.00  1.00           N
ATOM    477  CA  GLN A  34      62.920  80.098  63.959  1.00  1.00           C
ATOM    478  C   GLN A  34      62.640  79.571  65.381  1.00  1.00           C
ATOM    479  O   GLN A  34      62.321  80.368  66.258  1.00  1.00           O
ATOM    480  CB  GLN A  34      62.846  79.041  62.821  1.00  1.00           C
ATOM    481  CG  GLN A  34      63.054  79.585  61.397  1.00  1.00           C
ATOM    482  CD  GLN A  34      62.232  80.808  61.012  1.00  1.00           C
ATOM    483  NE2 GLN A  34      60.980  80.935  61.410  1.00  1.00           N
ATOM    484  OE1 GLN A  34      62.720  81.676  60.301  1.00  1.00           O
ATOM    485  H   GLN A  34      65.041  80.211  64.108  1.00  1.00           H
ATOM    486  HA  GLN A  34      62.138  80.832  63.768  1.00  1.00           H
ATOM    487  HB2 GLN A  34      63.619  78.294  62.989  1.00  1.00           H
ATOM    488  HB3 GLN A  34      61.882  78.537  62.864  1.00  1.00           H
ATOM    489  HG2 GLN A  34      64.103  79.853  61.279  1.00  1.00           H
ATOM    490  HG3 GLN A  34      62.826  78.793  60.687  1.00  1.00           H
ATOM    491 HE21 GLN A  34      60.541  80.285  62.071  1.00  1.00           H
ATOM    492 HE22 GLN A  34      60.496  81.770  61.143  1.00  1.00           H
ATOM    493  N   ASP A  35      62.731  78.248  65.594  1.00  1.00           N
ATOM    494  CA  ASP A  35      62.300  77.464  66.773  1.00  1.00           C
ATOM    495  C   ASP A  35      60.851  76.965  66.644  1.00  1.00           C
ATOM    496  O   ASP A  35      60.346  76.274  67.533  1.00  1.00           O
ATOM    497  CB  ASP A  35      62.703  77.972  68.195  1.00  1.00           C
ATOM    498  CG  ASP A  35      64.233  78.042  68.398  1.00  1.00           C
ATOM    499  OD1 ASP A  35      64.897  76.985  68.239  1.00  1.00           O
ATOM    500  OD2 ASP A  35      64.754  79.132  68.724  1.00  1.00           O1-
```

```
ATOM    501  H   ASP A  35      63.083  77.685  64.822  1.00  1.00           H
ATOM    502  HA  ASP A  35      62.866  76.538  66.677  1.00  1.00           H
ATOM    503  HB2 ASP A  35      62.249  78.945  68.385  1.00  1.00           H
ATOM    504  HB3 ASP A  35      62.307  77.280  68.940  1.00  1.00           H
ATOM    505  N   ASP A  36      60.207  77.266  65.505  1.00  1.00           N
ATOM    506  CA  ASP A  36      58.871  76.838  65.081  1.00  1.00           C
ATOM    507  C   ASP A  36      58.703  75.312  65.081  1.00  1.00           C
ATOM    508  O   ASP A  36      59.676  74.564  64.950  1.00  1.00           O
ATOM    509  CB  ASP A  36      58.518  77.400  63.686  1.00  1.00           C
ATOM    510  CG  ASP A  36      58.465  78.931  63.584  1.00  1.00           C
ATOM    511  OD1 ASP A  36      59.538  79.521  63.303  1.00  1.00           O
ATOM    512  OD2 ASP A  36      57.357  79.485  63.763  1.00  1.00           O1-
ATOM    513  H   ASP A  36      60.701  77.872  64.868  1.00  1.00           H
ATOM    514  HA  ASP A  36      58.155  77.249  65.793  1.00  1.00           H
ATOM    515  HB2 ASP A  36      59.247  77.022  62.966  1.00  1.00           H
ATOM    516  HB3 ASP A  36      57.544  77.005  63.391  1.00  1.00           H
ATOM    517  N   LEU A  37      57.451  74.865  65.242  1.00  1.00           N
ATOM    518  CA  LEU A  37      57.070  73.468  65.438  1.00  1.00           C
ATOM    519  C   LEU A  37      56.377  72.903  64.188  1.00  1.00           C
ATOM    520  O   LEU A  37      55.688  73.630  63.472  1.00  1.00           O
ATOM    521  CB  LEU A  37      56.099  73.358  66.640  1.00  1.00           C
ATOM    522  CG  LEU A  37      56.559  74.028  67.951  1.00  1.00           C
ATOM    523  CD1 LEU A  37      55.531  73.753  69.054  1.00  1.00           C
ATOM    524  CD2 LEU A  37      57.925  73.534  68.427  1.00  1.00           C
ATOM    525  H   LEU A  37      56.714  75.551  65.270  1.00  1.00           H
ATOM    526  HA  LEU A  37      57.960  72.879  65.641  1.00  1.00           H
ATOM    527  HB2 LEU A  37      55.145  73.802  66.352  1.00  1.00           H
ATOM    528  HB3 LEU A  37      55.926  72.298  66.833  1.00  1.00           H
ATOM    529  HG  LEU A  37      56.610  75.107  67.803  1.00  1.00           H
ATOM    530 HD11 LEU A  37      55.828  74.276  69.963  1.00  1.00           H
ATOM    531 HD12 LEU A  37      54.554  74.122  68.742  1.00  1.00           H
ATOM    532 HD13 LEU A  37      55.472  72.683  69.252  1.00  1.00           H
ATOM    533 HD21 LEU A  37      58.180  74.003  69.377  1.00  1.00           H
ATOM    534 HD22 LEU A  37      57.912  72.451  68.542  1.00  1.00           H
ATOM    535 HD23 LEU A  37      58.692  73.806  67.702  1.00  1.00           H
ATOM    536  N   PHE A  38      56.524  71.590  63.961  1.00  1.00           N
ATOM    537  CA  PHE A  38      56.112  70.872  62.749  1.00  1.00           C
ATOM    538  C   PHE A  38      55.247  69.650  63.096  1.00  1.00           C
ATOM    539  O   PHE A  38      55.231  69.202  64.244  1.00  1.00           O
ATOM    540  CB  PHE A  38      57.363  70.508  61.923  1.00  1.00           C
ATOM    541  CG  PHE A  38      58.167  71.744  61.557  1.00  1.00           C
ATOM    542  CD1 PHE A  38      59.305  72.110  62.305  1.00  1.00           C
ATOM    543  CD2 PHE A  38      57.716  72.586  60.524  1.00  1.00           C
ATOM    544  CE1 PHE A  38      59.977  73.311  62.021  1.00  1.00           C
ATOM    545  CE2 PHE A  38      58.394  73.783  60.232  1.00  1.00           C
ATOM    546  CZ  PHE A  38      59.525  74.147  60.985  1.00  1.00           C
ATOM    547  H   PHE A  38      57.058  71.073  64.646  1.00  1.00           H
ATOM    548  HA  PHE A  38      55.498  71.535  62.138  1.00  1.00           H
ATOM    549  HB2 PHE A  38      57.987  69.820  62.495  1.00  1.00           H
ATOM    550  HB3 PHE A  38      57.057  70.002  61.006  1.00  1.00           H
```

```
ATOM    551  HD1 PHE A  38      59.651  71.486  63.116  1.00  1.00           H
ATOM    552  HD2 PHE A  38      56.828  72.322  59.971  1.00  1.00           H
ATOM    553  HE1 PHE A  38      60.841  73.596  62.604  1.00  1.00           H
ATOM    554  HE2 PHE A  38      58.043  74.430  59.441  1.00  1.00           H
ATOM    555  HZ  PHE A  38      60.044  75.069  60.770  1.00  1.00           H
ATOM    556  N   VAL A  39      54.511  69.109  62.115  1.00  1.00           N
ATOM    557  CA  VAL A  39      53.519  68.054  62.316  1.00  1.00           C
ATOM    558  C   VAL A  39      54.244  66.698  62.189  1.00  1.00           C
ATOM    559  O   VAL A  39      54.278  66.093  61.114  1.00  1.00           O
ATOM    560  CB  VAL A  39      52.386  68.204  61.255  1.00  1.00           C
ATOM    561  CG1 VAL A  39      51.262  67.186  61.508  1.00  1.00           C
ATOM    562  CG2 VAL A  39      51.751  69.599  61.306  1.00  1.00           C
ATOM    563  H   VAL A  39      54.647  69.437  61.161  1.00  1.00           H
ATOM    564  HA  VAL A  39      53.082  68.141  63.312  1.00  1.00           H
ATOM    565  HB  VAL A  39      52.785  68.048  60.253  1.00  1.00           H
ATOM    566 HG11 VAL A  39      50.464  67.328  60.779  1.00  1.00           H
ATOM    567 HG12 VAL A  39      51.638  66.169  61.402  1.00  1.00           H
ATOM    568 HG13 VAL A  39      50.853  67.318  62.510  1.00  1.00           H
ATOM    569 HG21 VAL A  39      50.899  69.646  60.628  1.00  1.00           H
ATOM    570 HG22 VAL A  39      51.416  69.817  62.320  1.00  1.00           H
ATOM    571 HG23 VAL A  39      52.470  70.356  60.995  1.00  1.00           H
ATOM    572  N   GLY A  40      54.824  66.246  63.303  1.00  1.00           N
ATOM    573  CA  GLY A  40      55.585  65.011  63.432  1.00  1.00           C
ATOM    574  C   GLY A  40      54.888  64.031  64.370  1.00  1.00           C
ATOM    575  O   GLY A  40      54.483  64.403  65.473  1.00  1.00           O
ATOM    576  H   GLY A  40      54.776  66.846  64.122  1.00  1.00           H
ATOM    577  HA2 GLY A  40      55.712  64.545  62.459  1.00  1.00           H
ATOM    578  HA3 GLY A  40      56.570  65.233  63.841  1.00  1.00           H
ATOM    579  N   ALA A  41      54.762  62.767  63.958  1.00  1.00           N
ATOM    580  CA  ALA A  41      54.243  61.673  64.767  1.00  1.00           C
ATOM    581  C   ALA A  41      54.875  60.346  64.348  1.00  1.00           C
ATOM    582  O   ALA A  41      55.139  60.135  63.165  1.00  1.00           O
ATOM    583  CB  ALA A  41      52.708  61.642  64.739  1.00  1.00           C
ATOM    584  H   ALA A  41      55.048  62.546  62.999  1.00  1.00           H
ATOM    585  HA  ALA A  41      54.545  61.861  65.796  1.00  1.00           H
ATOM    586  HB1 ALA A  41      52.347  60.851  65.396  1.00  1.00           H
ATOM    587  HB2 ALA A  41      52.311  62.598  65.083  1.00  1.00           H
ATOM    588  HB3 ALA A  41      52.362  61.450  63.723  1.00  1.00           H
ATOM    589  N   ALA A  42      55.081  59.447  65.313  1.00  1.00           N
ATOM    590  CA  ALA A  42      55.664  58.128  65.140  1.00  1.00           C
ATOM    591  C   ALA A  42      54.798  57.073  65.828  1.00  1.00           C
ATOM    592  O   ALA A  42      54.385  57.248  66.977  1.00  1.00           O
ATOM    593  CB  ALA A  42      57.120  58.123  65.607  1.00  1.00           C
ATOM    594  H   ALA A  42      54.806  59.697  66.265  1.00  1.00           H
ATOM    595  HA  ALA A  42      55.670  57.894  64.079  1.00  1.00           H
ATOM    596  HB1 ALA A  42      57.737  58.685  64.909  1.00  1.00           H
ATOM    597  HB2 ALA A  42      57.204  58.548  66.606  1.00  1.00           H
ATOM    598  HB3 ALA A  42      57.468  57.098  65.628  1.00  1.00           H
ATOM    599  N   LEU A  43      54.486  56.006  65.090  1.00  1.00           N
ATOM    600  CA  LEU A  43      53.653  54.888  65.502  1.00  1.00           C
```

```
ATOM    601  C   LEU A  43      54.564  53.652  65.508  1.00  1.00           C
ATOM    602  O   LEU A  43      54.947  53.185  64.434  1.00  1.00           O
ATOM    603  CB  LEU A  43      52.498  54.688  64.478  1.00  1.00           C
ATOM    604  CG  LEU A  43      51.515  55.854  64.231  1.00  1.00           C
ATOM    605  CD1 LEU A  43      50.881  56.406  65.506  1.00  1.00           C
ATOM    606  CD2 LEU A  43      52.106  57.024  63.438  1.00  1.00           C
ATOM    607  H   LEU A  43      54.839  55.987  64.130  1.00  1.00           H
ATOM    608  HA  LEU A  43      53.242  55.050  66.499  1.00  1.00           H
ATOM    609  HB2 LEU A  43      52.921  54.410  63.511  1.00  1.00           H
ATOM    610  HB3 LEU A  43      51.910  53.835  64.820  1.00  1.00           H
ATOM    611  HG  LEU A  43      50.706  55.451  63.620  1.00  1.00           H
ATOM    612 HD11 LEU A  43      50.178  57.202  65.258  1.00  1.00           H
ATOM    613 HD12 LEU A  43      50.330  55.608  65.996  1.00  1.00           H
ATOM    614 HD13 LEU A  43      51.650  56.798  66.171  1.00  1.00           H
ATOM    615 HD21 LEU A  43      51.301  57.612  62.998  1.00  1.00           H
ATOM    616 HD22 LEU A  43      52.702  57.671  64.079  1.00  1.00           H
ATOM    617 HD23 LEU A  43      52.733  56.631  62.642  1.00  1.00           H
ATOM    618  N   GLY A  44      54.910  53.131  66.690  1.00  1.00           N
ATOM    619  CA  GLY A  44      55.826  52.007  66.874  1.00  1.00           C
ATOM    620  C   GLY A  44      55.073  50.780  67.380  1.00  1.00           C
ATOM    621  O   GLY A  44      54.318  50.883  68.344  1.00  1.00           O
ATOM    622  H   GLY A  44      54.573  53.589  67.535  1.00  1.00           H
ATOM    623  HA2 GLY A  44      56.322  51.758  65.940  1.00  1.00           H
ATOM    624  HA3 GLY A  44      56.594  52.268  67.603  1.00  1.00           H
ATOM    625  N   ILE A  45      55.251  49.630  66.720  1.00  1.00           N
ATOM    626  CA  ILE A  45      54.643  48.341  67.054  1.00  1.00           C
ATOM    627  C   ILE A  45      55.789  47.339  67.255  1.00  1.00           C
ATOM    628  O   ILE A  45      56.635  47.195  66.372  1.00  1.00           O
ATOM    629  CB  ILE A  45      53.675  47.878  65.918  1.00  1.00           C
ATOM    630  CG1 ILE A  45      52.511  48.876  65.725  1.00  1.00           C
ATOM    631  CG2 ILE A  45      53.123  46.472  66.234  1.00  1.00           C
ATOM    632  CD1 ILE A  45      51.627  48.596  64.503  1.00  1.00           C
ATOM    633  H   ILE A  45      55.914  49.627  65.938  1.00  1.00           H
ATOM    634  HA  ILE A  45      54.077  48.423  67.984  1.00  1.00           H
ATOM    635  HB  ILE A  45      54.242  47.822  64.987  1.00  1.00           H
ATOM    636 HG12 ILE A  45      51.886  48.862  66.617  1.00  1.00           H
ATOM    637 HG13 ILE A  45      52.915  49.880  65.599  1.00  1.00           H
ATOM    638 HG21 ILE A  45      52.474  46.125  65.432  1.00  1.00           H
ATOM    639 HG22 ILE A  45      53.930  45.746  66.331  1.00  1.00           H
ATOM    640 HG23 ILE A  45      52.554  46.496  67.166  1.00  1.00           H
ATOM    641 HD11 ILE A  45      50.926  49.421  64.373  1.00  1.00           H
ATOM    642 HD12 ILE A  45      52.245  48.512  63.609  1.00  1.00           H
ATOM    643 HD13 ILE A  45      51.054  47.680  64.644  1.00  1.00           H
ATOM    644  N   GLU A  46      55.783  46.609  68.374  1.00  1.00           N
ATOM    645  CA  GLU A  46      56.671  45.480  68.624  1.00  1.00           C
ATOM    646  C   GLU A  46      55.887  44.197  68.332  1.00  1.00           C
ATOM    647  O   GLU A  46      54.806  43.980  68.881  1.00  1.00           O
ATOM    648  CB  GLU A  46      57.118  45.510  70.099  1.00  1.00           C
ATOM    649  CG  GLU A  46      58.305  46.476  70.183  1.00  1.00           C
ATOM    650  CD  GLU A  46      58.418  47.121  71.546  1.00  1.00           C
```

```
ATOM    651  OE1 GLU A  46      57.677  48.089  71.820  1.00  1.00           O
ATOM    652  OE2 GLU A  46      59.286  46.698  72.338  1.00  1.00           O1-
ATOM    653  H   GLU A  46      55.062  46.789  69.060  1.00  1.00           H
ATOM    654  HA  GLU A  46      57.549  45.509  67.983  1.00  1.00           H
ATOM    655  HB2 GLU A  46      56.292  45.859  70.722  1.00  1.00           H
ATOM    656  HB3 GLU A  46      57.411  44.523  70.438  1.00  1.00           H
ATOM    657  HG2 GLU A  46      59.226  45.939  69.948  1.00  1.00           H
ATOM    658  HG3 GLU A  46      58.192  47.284  69.459  1.00  1.00           H
ATOM    659  N   LEU A  47      56.447  43.349  67.463  1.00  1.00           N
ATOM    660  CA  LEU A  47      55.920  42.028  67.116  1.00  1.00           C
ATOM    661  C   LEU A  47      56.571  40.955  68.009  1.00  1.00           C
ATOM    662  O   LEU A  47      56.108  39.819  68.068  1.00  1.00           O
ATOM    663  CB  LEU A  47      56.262  41.708  65.640  1.00  1.00           C
ATOM    664  CG  LEU A  47      55.627  42.586  64.539  1.00  1.00           C
ATOM    665  CD1 LEU A  47      54.108  42.686  64.681  1.00  1.00           C
ATOM    666  CD2 LEU A  47      56.194  44.005  64.440  1.00  1.00           C
ATOM    667  H   LEU A  47      57.324  43.630  67.045  1.00  1.00           H
ATOM    668  HA  LEU A  47      54.840  41.995  67.265  1.00  1.00           H
ATOM    669  HB2 LEU A  47      57.346  41.733  65.517  1.00  1.00           H
ATOM    670  HB3 LEU A  47      55.945  40.682  65.446  1.00  1.00           H
ATOM    671  HG  LEU A  47      55.835  42.098  63.586  1.00  1.00           H
ATOM    672 HD11 LEU A  47      53.691  43.202  63.816  1.00  1.00           H
ATOM    673 HD12 LEU A  47      53.683  41.684  64.732  1.00  1.00           H
ATOM    674 HD13 LEU A  47      53.849  43.237  65.585  1.00  1.00           H
ATOM    675 HD21 LEU A  47      55.798  44.476  63.543  1.00  1.00           H
ATOM    676 HD22 LEU A  47      55.889  44.608  65.293  1.00  1.00           H
ATOM    677 HD23 LEU A  47      57.282  43.973  64.381  1.00  1.00           H
ATOM    678  N   THR A  48      57.624  41.322  68.738  1.00  1.00           N
ATOM    679  CA  THR A  48      58.254  40.588  69.819  1.00  1.00           C
ATOM    680  C   THR A  48      58.718  41.663  70.826  1.00  1.00           C
ATOM    681  O   THR A  48      59.099  42.748  70.375  1.00  1.00           O
ATOM    682  CB  THR A  48      59.325  39.624  69.232  1.00  1.00           C
ATOM    683  CG2 THR A  48      60.459  40.314  68.478  1.00  1.00           C
ATOM    684  OG1 THR A  48      59.914  38.880  70.269  1.00  1.00           O
ATOM    685  H   THR A  48      58.006  42.249  68.578  1.00  1.00           H
ATOM    686  HA  THR A  48      57.488  39.977  70.296  1.00  1.00           H
ATOM    687  HB  THR A  48      58.841  38.918  68.560  1.00  1.00           H
ATOM    688  HG1 THR A  48      60.140  38.023  69.900  1.00  1.00           H
ATOM    689 HG21 THR A  48      61.143  39.565  68.080  1.00  1.00           H
ATOM    690 HG22 THR A  48      60.056  40.891  67.646  1.00  1.00           H
ATOM    691 HG23 THR A  48      61.004  40.971  69.153  1.00  1.00           H
ATOM    692  N   PRO A  49      58.686  41.439  72.160  1.00  1.00           N
ATOM    693  CA  PRO A  49      59.086  42.446  73.165  1.00  1.00           C
ATOM    694  C   PRO A  49      60.622  42.614  73.229  1.00  1.00           C
ATOM    695  O   PRO A  49      61.257  42.372  74.255  1.00  1.00           O
ATOM    696  CB  PRO A  49      58.465  41.928  74.475  1.00  1.00           C
ATOM    697  CG  PRO A  49      58.508  40.411  74.305  1.00  1.00           C
ATOM    698  CD  PRO A  49      58.210  40.229  72.817  1.00  1.00           C
ATOM    699  HA  PRO A  49      58.647  43.415  72.920  1.00  1.00           H
ATOM    700  HB2 PRO A  49      58.999  42.258  75.366  1.00  1.00           H
```

ATOM	701	HB3	PRO	A	49	57.425	42.252	74.530	1.00	1.00	H
ATOM	702	HG2	PRO	A	49	59.510	40.042	74.528	1.00	1.00	H
ATOM	703	HG3	PRO	A	49	57.766	39.912	74.930	1.00	1.00	H
ATOM	704	HD2	PRO	A	49	58.717	39.341	72.446	1.00	1.00	H
ATOM	705	HD3	PRO	A	49	57.133	40.138	72.668	1.00	1.00	H
ATOM	706	N	TRP	A	50	61.206	42.995	72.093	1.00	1.00	N
ATOM	707	CA	TRP	A	50	62.622	43.115	71.794	1.00	1.00	C
ATOM	708	C	TRP	A	50	62.824	43.831	70.442	1.00	1.00	C
ATOM	709	O	TRP	A	50	63.790	44.585	70.306	1.00	1.00	O
ATOM	710	CB	TRP	A	50	63.297	41.720	71.802	1.00	1.00	C
ATOM	711	CG	TRP	A	50	64.692	41.741	71.239	1.00	1.00	C
ATOM	712	CD1	TRP	A	50	65.711	42.501	71.704	1.00	1.00	C
ATOM	713	CD2	TRP	A	50	65.203	41.114	70.020	1.00	1.00	C
ATOM	714	CE2	TRP	A	50	66.521	41.608	69.774	1.00	1.00	C
ATOM	715	CE3	TRP	A	50	64.674	40.202	69.079	1.00	1.00	C
ATOM	716	NE1	TRP	A	50	66.772	42.463	70.825	1.00	1.00	N
ATOM	717	CZ2	TRP	A	50	67.266	41.230	68.647	1.00	1.00	C
ATOM	718	CZ3	TRP	A	50	65.421	39.802	67.955	1.00	1.00	C
ATOM	719	CH2	TRP	A	50	66.712	40.316	67.736	1.00	1.00	C
ATOM	720	H	TRP	A	50	60.547	43.223	71.355	1.00	1.00	H
ATOM	721	HA	TRP	A	50	63.080	43.720	72.574	1.00	1.00	H
ATOM	722	HB2	TRP	A	50	63.331	41.340	72.824	1.00	1.00	H
ATOM	723	HB3	TRP	A	50	62.696	41.032	71.206	1.00	1.00	H
ATOM	724	HD1	TRP	A	50	65.656	43.125	72.585	1.00	1.00	H
ATOM	725	HE1	TRP	A	50	67.571	43.094	70.904	1.00	1.00	H
ATOM	726	HE3	TRP	A	50	63.680	39.808	69.226	1.00	1.00	H
ATOM	727	HZ2	TRP	A	50	68.253	41.638	68.487	1.00	1.00	H
ATOM	728	HZ3	TRP	A	50	64.998	39.098	67.252	1.00	1.00	H
ATOM	729	HH2	TRP	A	50	67.274	40.010	66.866	1.00	1.00	H
ATOM	730	N	LEU	A	51	61.961	43.565	69.448	1.00	1.00	N
ATOM	731	CA	LEU	A	51	62.128	43.952	68.047	1.00	1.00	C
ATOM	732	C	LEU	A	51	60.762	44.352	67.479	1.00	1.00	C
ATOM	733	O	LEU	A	51	59.776	43.615	67.608	1.00	1.00	O
ATOM	734	CB	LEU	A	51	62.856	42.823	67.275	1.00	1.00	C
ATOM	735	CG	LEU	A	51	63.261	43.139	65.821	1.00	1.00	C
ATOM	736	CD1	LEU	A	51	64.285	42.096	65.360	1.00	1.00	C
ATOM	737	CD2	LEU	A	51	62.106	43.121	64.811	1.00	1.00	C
ATOM	738	H	LEU	A	51	61.147	42.995	69.647	1.00	1.00	H
ATOM	739	HA	LEU	A	51	62.772	44.830	68.019	1.00	1.00	H
ATOM	740	HB2	LEU	A	51	63.768	42.606	67.832	1.00	1.00	H
ATOM	741	HB3	LEU	A	51	62.247	41.923	67.279	1.00	1.00	H
ATOM	742	HG	LEU	A	51	63.739	44.114	65.787	1.00	1.00	H
ATOM	743	HD11	LEU	A	51	64.611	42.321	64.345	1.00	1.00	H
ATOM	744	HD12	LEU	A	51	65.157	42.133	66.013	1.00	1.00	H
ATOM	745	HD13	LEU	A	51	63.848	41.098	65.389	1.00	1.00	H
ATOM	746	HD21	LEU	A	51	62.503	43.183	63.798	1.00	1.00	H
ATOM	747	HD22	LEU	A	51	61.524	42.207	64.919	1.00	1.00	H
ATOM	748	HD23	LEU	A	51	61.463	43.986	64.947	1.00	1.00	H
ATOM	749	N	GLY	A	52	60.695	45.544	66.892	1.00	1.00	N
ATOM	750	CA	GLY	A	52	59.510	46.126	66.294	1.00	1.00	C

ATOM	751	C	GLY	A	52	59.805	46.856	64.992	1.00	1.00	C
ATOM	752	O	GLY	A	52	60.928	46.853	64.490	1.00	1.00	O
ATOM	753	H	GLY	A	52	61.570	46.060	66.769	1.00	1.00	H
ATOM	754	HA2	GLY	A	52	58.774	45.352	66.083	1.00	1.00	H
ATOM	755	HA3	GLY	A	52	59.077	46.836	66.997	1.00	1.00	H
ATOM	756	N	PHE	A	53	58.782	47.551	64.499	1.00	1.00	N
ATOM	757	CA	PHE	A	53	58.857	48.498	63.398	1.00	1.00	C
ATOM	758	C	PHE	A	53	58.122	49.766	63.836	1.00	1.00	C
ATOM	759	O	PHE	A	53	57.123	49.694	64.560	1.00	1.00	O
ATOM	760	CB	PHE	A	53	58.268	47.903	62.104	1.00	1.00	C
ATOM	761	CG	PHE	A	53	58.846	46.574	61.645	1.00	1.00	C
ATOM	762	CD1	PHE	A	53	58.054	45.411	61.675	1.00	1.00	C
ATOM	763	CD2	PHE	A	53	60.162	46.497	61.154	1.00	1.00	C
ATOM	764	CE1	PHE	A	53	58.578	44.179	61.243	1.00	1.00	C
ATOM	765	CE2	PHE	A	53	60.693	45.267	60.727	1.00	1.00	C
ATOM	766	CZ	PHE	A	53	59.901	44.106	60.773	1.00	1.00	C
ATOM	767	H	PHE	A	53	57.903	47.517	65.017	1.00	1.00	H
ATOM	768	HA	PHE	A	53	59.902	48.748	63.215	1.00	1.00	H
ATOM	769	HB2	PHE	A	53	57.192	47.790	62.241	1.00	1.00	H
ATOM	770	HB3	PHE	A	53	58.413	48.628	61.302	1.00	1.00	H
ATOM	771	HD1	PHE	A	53	57.036	45.466	62.025	1.00	1.00	H
ATOM	772	HD2	PHE	A	53	60.767	47.385	61.090	1.00	1.00	H
ATOM	773	HE1	PHE	A	53	57.963	43.290	61.268	1.00	1.00	H
ATOM	774	HE2	PHE	A	53	61.707	45.218	60.353	1.00	1.00	H
ATOM	775	HZ	PHE	A	53	60.311	43.164	60.440	1.00	1.00	H
ATOM	776	N	GLU	A	54	58.603	50.918	63.370	1.00	1.00	N
ATOM	777	CA	GLU	A	54	58.044	52.237	63.579	1.00	1.00	C
ATOM	778	C	GLU	A	54	57.841	52.925	62.228	1.00	1.00	C
ATOM	779	O	GLU	A	54	58.753	52.972	61.402	1.00	1.00	O
ATOM	780	CB	GLU	A	54	58.888	53.022	64.614	1.00	1.00	C
ATOM	781	CG	GLU	A	54	58.476	54.494	64.646	1.00	1.00	C
ATOM	782	CD	GLU	A	54	59.161	55.189	65.795	1.00	1.00	C
ATOM	783	OE1	GLU	A	54	59.927	56.148	65.575	1.00	1.00	O
ATOM	784	OE2	GLU	A	54	58.918	54.773	66.945	1.00	1.00	O1-
ATOM	785	H	GLU	A	54	59.441	50.868	62.789	1.00	1.00	H
ATOM	786	HA	GLU	A	54	57.056	52.114	64.011	1.00	1.00	H
ATOM	787	HB2	GLU	A	54	58.738	52.563	65.592	1.00	1.00	H
ATOM	788	HB3	GLU	A	54	59.945	52.956	64.358	1.00	1.00	H
ATOM	789	HG2	GLU	A	54	58.758	54.985	63.713	1.00	1.00	H
ATOM	790	HG3	GLU	A	54	57.398	54.574	64.784	1.00	1.00	H
ATOM	791	N	ALA	A	55	56.647	53.480	62.013	1.00	1.00	N
ATOM	792	CA	ALA	A	55	56.348	54.383	60.916	1.00	1.00	C
ATOM	793	C	ALA	A	55	56.328	55.789	61.526	1.00	1.00	C
ATOM	794	O	ALA	A	55	55.508	56.074	62.404	1.00	1.00	O
ATOM	795	CB	ALA	A	55	54.965	54.031	60.352	1.00	1.00	C
ATOM	796	H	ALA	A	55	55.933	53.361	62.731	1.00	1.00	H
ATOM	797	HA	ALA	A	55	57.097	54.314	60.126	1.00	1.00	H
ATOM	798	HB1	ALA	A	55	54.735	54.700	59.523	1.00	1.00	H
ATOM	799	HB2	ALA	A	55	54.976	53.007	59.977	1.00	1.00	H
ATOM	800	HB3	ALA	A	55	54.200	54.119	61.125	1.00	1.00	H

```
ATOM    801  N   GLU A  56      57.264  56.633  61.089  1.00  1.00           N
ATOM    802  CA  GLU A  56      57.416  58.030  61.457  1.00  1.00           C
ATOM    803  C   GLU A  56      56.922  58.864  60.268  1.00  1.00           C
ATOM    804  O   GLU A  56      57.349  58.669  59.127  1.00  1.00           O
ATOM    805  CB  GLU A  56      58.901  58.306  61.777  1.00  1.00           C
ATOM    806  CG  GLU A  56      59.091  59.796  62.091  1.00  1.00           C
ATOM    807  CD  GLU A  56      60.527  60.191  62.405  1.00  1.00           C
ATOM    808  OE1 GLU A  56      61.469  59.498  61.965  1.00  1.00           O
ATOM    809  OE2 GLU A  56      60.691  61.224  63.085  1.00  1.00           O1-
ATOM    810  H   GLU A  56      57.884  56.298  60.346  1.00  1.00           H
ATOM    811  HA  GLU A  56      56.819  58.258  62.336  1.00  1.00           H
ATOM    812  HB2 GLU A  56      59.207  57.710  62.638  1.00  1.00           H
ATOM    813  HB3 GLU A  56      59.507  58.030  60.918  1.00  1.00           H
ATOM    814  HG2 GLU A  56      58.783  60.384  61.229  1.00  1.00           H
ATOM    815  HG3 GLU A  56      58.457  60.064  62.935  1.00  1.00           H
ATOM    816  N   TYR A  57      55.999  59.778  60.552  1.00  1.00           N
ATOM    817  CA  TYR A  57      55.329  60.663  59.620  1.00  1.00           C
ATOM    818  C   TYR A  57      55.644  62.087  60.076  1.00  1.00           C
ATOM    819  O   TYR A  57      55.193  62.490  61.148  1.00  1.00           O
ATOM    820  CB  TYR A  57      53.816  60.375  59.711  1.00  1.00           C
ATOM    821  CG  TYR A  57      53.388  59.007  59.222  1.00  1.00           C
ATOM    822  CD1 TYR A  57      53.053  58.813  57.870  1.00  1.00           C
ATOM    823  CD2 TYR A  57      53.291  57.936  60.128  1.00  1.00           C
ATOM    824  CE1 TYR A  57      52.618  57.553  57.424  1.00  1.00           C
ATOM    825  CE2 TYR A  57      52.828  56.683  59.687  1.00  1.00           C
ATOM    826  CZ  TYR A  57      52.500  56.490  58.335  1.00  1.00           C
ATOM    827  OH  TYR A  57      52.073  55.273  57.902  1.00  1.00           O
ATOM    828  H   TYR A  57      55.699  59.857  61.525  1.00  1.00           H
ATOM    829  HA  TYR A  57      55.678  60.506  58.598  1.00  1.00           H
ATOM    830  HB2 TYR A  57      53.489  60.491  60.746  1.00  1.00           H
ATOM    831  HB3 TYR A  57      53.281  61.122  59.128  1.00  1.00           H
ATOM    832  HD1 TYR A  57      53.121  59.635  57.172  1.00  1.00           H
ATOM    833  HD2 TYR A  57      53.567  58.082  61.163  1.00  1.00           H
ATOM    834  HE1 TYR A  57      52.365  57.411  56.384  1.00  1.00           H
ATOM    835  HE2 TYR A  57      52.723  55.867  60.384  1.00  1.00           H
ATOM    836  HH  TYR A  57      51.914  55.272  56.955  1.00  1.00           H
ATOM    837  N   ASN A  58      56.399  62.842  59.275  1.00  1.00           N
ATOM    838  CA  ASN A  58      56.772  64.238  59.503  1.00  1.00           C
ATOM    839  C   ASN A  58      56.288  65.083  58.321  1.00  1.00           C
ATOM    840  O   ASN A  58      56.494  64.709  57.169  1.00  1.00           O
ATOM    841  CB  ASN A  58      58.295  64.348  59.754  1.00  1.00           C
ATOM    842  CG  ASN A  58      58.715  63.822  61.123  1.00  1.00           C
ATOM    843  ND2 ASN A  58      59.666  62.905  61.164  1.00  1.00           N
ATOM    844  OD1 ASN A  58      58.201  64.238  62.147  1.00  1.00           O
ATOM    845  H   ASN A  58      56.719  62.445  58.389  1.00  1.00           H
ATOM    846  HA  ASN A  58      56.261  64.606  60.390  1.00  1.00           H
ATOM    847  HB2 ASN A  58      58.823  63.801  58.977  1.00  1.00           H
ATOM    848  HB3 ASN A  58      58.595  65.393  59.701  1.00  1.00           H
ATOM    849 HD21 ASN A  58      60.104  62.603  60.313  1.00  1.00           H
ATOM    850 HD22 ASN A  58      59.935  62.464  62.055  1.00  1.00           H
```

ATOM	851	N	GLN	A	59	55.574	66.185	58.578	1.00	1.00	N
ATOM	852	CA	GLN	A	59	54.880	66.980	57.565	1.00	1.00	C
ATOM	853	C	GLN	A	59	54.975	68.480	57.880	1.00	1.00	C
ATOM	854	O	GLN	A	59	55.028	68.879	59.048	1.00	1.00	O
ATOM	855	CB	GLN	A	59	53.409	66.521	57.371	1.00	1.00	C
ATOM	856	CG	GLN	A	59	53.182	65.084	56.855	1.00	1.00	C
ATOM	857	CD	GLN	A	59	53.488	63.937	57.813	1.00	1.00	C
ATOM	858	NE2	GLN	A	59	53.271	64.088	59.109	1.00	1.00	N
ATOM	859	OE1	GLN	A	59	53.918	62.877	57.386	1.00	1.00	O
ATOM	860	H	GLN	A	59	55.385	66.416	59.555	1.00	1.00	H
ATOM	861	HA	GLN	A	59	55.388	66.832	56.612	1.00	1.00	H
ATOM	862	HB2	GLN	A	59	52.867	66.655	58.307	1.00	1.00	H
ATOM	863	HB3	GLN	A	59	52.954	67.193	56.642	1.00	1.00	H
ATOM	864	HG2	GLN	A	59	52.133	64.989	56.579	1.00	1.00	H
ATOM	865	HG3	GLN	A	59	53.777	64.933	55.955	1.00	1.00	H
ATOM	866	HE21	GLN	A	59	53.009	64.982	59.499	1.00	1.00	H
ATOM	867	HE22	GLN	A	59	53.544	63.335	59.722	1.00	1.00	H
ATOM	868	N	VAL	A	60	54.928	69.298	56.827	1.00	1.00	N
ATOM	869	CA	VAL	A	60	54.862	70.754	56.839	1.00	1.00	C
ATOM	870	C	VAL	A	60	53.989	71.168	55.643	1.00	1.00	C
ATOM	871	O	VAL	A	60	54.094	70.563	54.579	1.00	1.00	O
ATOM	872	CB	VAL	A	60	56.295	71.357	56.899	1.00	1.00	C
ATOM	873	CG1	VAL	A	60	57.106	71.109	55.622	1.00	1.00	C
ATOM	874	CG2	VAL	A	60	56.220	72.864	57.161	1.00	1.00	C
ATOM	875	H	VAL	A	60	54.910	68.862	55.901	1.00	1.00	H
ATOM	876	HA	VAL	A	60	54.339	71.054	57.748	1.00	1.00	H
ATOM	877	HB	VAL	A	60	56.825	70.900	57.736	1.00	1.00	H
ATOM	878	HG11	VAL	A	60	58.121	71.485	55.751	1.00	1.00	H
ATOM	879	HG12	VAL	A	60	57.149	70.039	55.421	1.00	1.00	H
ATOM	880	HG13	VAL	A	60	56.648	71.616	54.772	1.00	1.00	H
ATOM	881	HG21	VAL	A	60	57.225	73.253	57.327	1.00	1.00	H
ATOM	882	HG22	VAL	A	60	55.780	73.376	56.305	1.00	1.00	H
ATOM	883	HG23	VAL	A	60	55.617	73.064	58.048	1.00	1.00	H
ATOM	884	N	LYS	A	61	53.107	72.163	55.779	1.00	1.00	N
ATOM	885	CA	LYS	A	61	52.224	72.609	54.704	1.00	1.00	C
ATOM	886	C	LYS	A	61	51.903	74.095	54.892	1.00	1.00	C
ATOM	887	O	LYS	A	61	51.772	74.556	56.025	1.00	1.00	O
ATOM	888	CB	LYS	A	61	50.990	71.676	54.593	1.00	1.00	C
ATOM	889	CG	LYS	A	61	50.256	71.782	53.250	1.00	1.00	C
ATOM	890	CD	LYS	A	61	49.198	70.677	53.098	1.00	1.00	C
ATOM	891	CE	LYS	A	61	48.442	70.831	51.773	1.00	1.00	C
ATOM	892	NZ	LYS	A	61	47.377	71.842	51.875	1.00	1.00	N1+
ATOM	893	H	LYS	A	61	53.030	72.647	56.665	1.00	1.00	H
ATOM	894	HA	LYS	A	61	52.781	72.524	53.771	1.00	1.00	H
ATOM	895	HB2	LYS	A	61	51.326	70.644	54.704	1.00	1.00	H
ATOM	896	HB3	LYS	A	61	50.297	71.895	55.406	1.00	1.00	H
ATOM	897	HG2	LYS	A	61	49.793	72.766	53.168	1.00	1.00	H
ATOM	898	HG3	LYS	A	61	50.976	71.657	52.445	1.00	1.00	H
ATOM	899	HD2	LYS	A	61	49.704	69.709	53.099	1.00	1.00	H
ATOM	900	HD3	LYS	A	61	48.499	70.707	53.935	1.00	1.00	H

```
ATOM    901  HE2 LYS A  61      49.153  71.124  50.999  1.00  1.00           H
ATOM    902  HE3 LYS A  61      47.998  69.875  51.493  1.00  1.00           H
ATOM    903  HZ1 LYS A  61      46.512  71.455  52.217  1.00  1.00           H
ATOM    904  HZ2 LYS A  61      47.633  72.561  52.555  1.00  1.00           H
ATOM    905  HZ3 LYS A  61      47.204  72.316  50.989  1.00  1.00           H
ATOM    906  N   GLY A  62      51.783  74.833  53.788  1.00  1.00           N
ATOM    907  CA  GLY A  62      51.414  76.236  53.733  1.00  1.00           C
ATOM    908  C   GLY A  62      50.143  76.354  52.904  1.00  1.00           C
ATOM    909  O   GLY A  62      50.212  76.468  51.679  1.00  1.00           O
ATOM    910  H   GLY A  62      51.932  74.360  52.895  1.00  1.00           H
ATOM    911  HA2 GLY A  62      51.244  76.639  54.732  1.00  1.00           H
ATOM    912  HA3 GLY A  62      52.206  76.808  53.251  1.00  1.00           H
ATOM    913  N   ASP A  63      48.993  76.289  53.581  1.00  1.00           N
ATOM    914  CA  ASP A  63      47.661  76.436  52.997  1.00  1.00           C
ATOM    915  C   ASP A  63      47.295  77.911  52.850  1.00  1.00           C
ATOM    916  O   ASP A  63      47.513  78.719  53.750  1.00  1.00           O
ATOM    917  CB  ASP A  63      46.575  75.779  53.884  1.00  1.00           C
ATOM    918  CG  ASP A  63      46.452  74.291  53.553  1.00  1.00           C
ATOM    919  OD1 ASP A  63      45.490  73.912  52.845  1.00  1.00           O
ATOM    920  OD2 ASP A  63      47.355  73.520  53.951  1.00  1.00           O1-
ATOM    921  H   ASP A  63      49.035  76.168  54.583  1.00  1.00           H
ATOM    922  HA  ASP A  63      47.648  75.967  52.013  1.00  1.00           H
ATOM    923  HB2 ASP A  63      46.815  75.917  54.940  1.00  1.00           H
ATOM    924  HB3 ASP A  63      45.611  76.254  53.690  1.00  1.00           H
ATOM    925  N   VAL A  64      46.707  78.212  51.695  1.00  1.00           N
ATOM    926  CA  VAL A  64      46.143  79.467  51.226  1.00  1.00           C
ATOM    927  C   VAL A  64      44.998  79.074  50.270  1.00  1.00           C
ATOM    928  O   VAL A  64      45.081  78.013  49.641  1.00  1.00           O
ATOM    929  CB  VAL A  64      47.245  80.378  50.618  1.00  1.00           C
ATOM    930  CG1 VAL A  64      48.141  81.023  51.681  1.00  1.00           C
ATOM    931  CG2 VAL A  64      48.153  79.628  49.628  1.00  1.00           C
ATOM    932  H   VAL A  64      46.602  77.438  51.055  1.00  1.00           H
ATOM    933  HA  VAL A  64      45.700  79.987  52.076  1.00  1.00           H
ATOM    934  HB  VAL A  64      46.745  81.182  50.079  1.00  1.00           H
ATOM    935 HG11 VAL A  64      48.764  81.788  51.220  1.00  1.00           H
ATOM    936 HG12 VAL A  64      47.531  81.480  52.460  1.00  1.00           H
ATOM    937 HG13 VAL A  64      48.789  80.275  52.136  1.00  1.00           H
ATOM    938 HG21 VAL A  64      48.888  80.314  49.207  1.00  1.00           H
ATOM    939 HG22 VAL A  64      48.701  78.833  50.133  1.00  1.00           H
ATOM    940 HG23 VAL A  64      47.562  79.200  48.818  1.00  1.00           H
ATOM    941  N   ASP A  65      43.913  79.856  50.204  1.00  1.00           N
ATOM    942  CA  ASP A  65      42.679  79.507  49.485  1.00  1.00           C
ATOM    943  C   ASP A  65      42.665  80.158  48.086  1.00  1.00           C
ATOM    944  O   ASP A  65      42.342  79.493  47.102  1.00  1.00           O
ATOM    945  CB  ASP A  65      41.441  79.992  50.306  1.00  1.00           C
ATOM    946  CG  ASP A  65      41.268  81.514  50.471  1.00  1.00           C
ATOM    947  OD1 ASP A  65      40.110  81.964  50.623  1.00  1.00           O
ATOM    948  OD2 ASP A  65      42.275  82.265  50.440  1.00  1.00           O1-
ATOM    949  H   ASP A  65      43.887  80.717  50.738  1.00  1.00           H
ATOM    950  HA  ASP A  65      42.610  78.425  49.365  1.00  1.00           H
```

```
ATOM    951  HB2 ASP A  65      40.548  79.598  49.818  1.00  1.00           H
ATOM    952  HB3 ASP A  65      41.490  79.543  51.299  1.00  1.00           H
ATOM    953  N   GLY A  66      42.994  81.450  48.006  1.00  1.00           N
ATOM    954  CA  GLY A  66      42.862  82.281  46.819  1.00  1.00           C
ATOM    955  C   GLY A  66      41.835  83.409  46.965  1.00  1.00           C
ATOM    956  O   GLY A  66      41.330  83.870  45.942  1.00  1.00           O
ATOM    957  H   GLY A  66      43.199  81.892  48.899  1.00  1.00           H
ATOM    958  HA2 GLY A  66      43.820  82.741  46.603  1.00  1.00           H
ATOM    959  HA3 GLY A  66      42.571  81.671  45.964  1.00  1.00           H
ATOM    960  N   ALA A  67      41.484  83.812  48.196  1.00  1.00           N
ATOM    961  CA  ALA A  67      40.643  84.969  48.495  1.00  1.00           C
ATOM    962  C   ALA A  67      40.915  85.481  49.914  1.00  1.00           C
ATOM    963  O   ALA A  67      41.389  86.599  50.117  1.00  1.00           O
ATOM    964  CB  ALA A  67      39.156  84.654  48.249  1.00  1.00           C
ATOM    965  H   ALA A  67      41.872  83.305  48.988  1.00  1.00           H
ATOM    966  HA  ALA A  67      40.916  85.770  47.807  1.00  1.00           H
ATOM    967  HB1 ALA A  67      38.555  85.533  48.483  1.00  1.00           H
ATOM    968  HB2 ALA A  67      39.002  84.392  47.202  1.00  1.00           H
ATOM    969  HB3 ALA A  67      38.836  83.821  48.877  1.00  1.00           H
ATOM    970  N   SER A  68      40.626  84.639  50.910  1.00  1.00           N
ATOM    971  CA  SER A  68      40.684  84.935  52.342  1.00  1.00           C
ATOM    972  C   SER A  68      42.141  84.969  52.841  1.00  1.00           C
ATOM    973  O   SER A  68      42.435  85.616  53.845  1.00  1.00           O
ATOM    974  CB  SER A  68      39.949  83.813  53.112  1.00  1.00           C
ATOM    975  OG  SER A  68      38.852  83.261  52.404  1.00  1.00           O
ATOM    976  H   SER A  68      40.319  83.700  50.654  1.00  1.00           H
ATOM    977  HA  SER A  68      40.200  85.892  52.538  1.00  1.00           H
ATOM    978  HB2 SER A  68      40.649  83.001  53.317  1.00  1.00           H
ATOM    979  HB3 SER A  68      39.601  84.210  54.066  1.00  1.00           H
ATOM    980  HG  SER A  68      39.229  82.678  51.692  1.00  1.00           H
ATOM    981  N   ALA A  69      43.056  84.304  52.121  1.00  1.00           N
ATOM    982  CA  ALA A  69      44.494  84.249  52.363  1.00  1.00           C
ATOM    983  C   ALA A  69      45.230  85.583  52.149  1.00  1.00           C
ATOM    984  O   ALA A  69      46.421  85.659  52.448  1.00  1.00           O
ATOM    985  CB  ALA A  69      45.094  83.207  51.410  1.00  1.00           C
ATOM    986  H   ALA A  69      42.700  83.692  51.381  1.00  1.00           H
ATOM    987  HA  ALA A  69      44.665  83.926  53.391  1.00  1.00           H
ATOM    988  HB1 ALA A  69      46.180  83.202  51.500  1.00  1.00           H
ATOM    989  HB2 ALA A  69      44.713  82.221  51.670  1.00  1.00           H
ATOM    990  HB3 ALA A  69      44.817  83.448  50.384  1.00  1.00           H
ATOM    991  N   GLY A  70      44.549  86.616  51.640  1.00  1.00           N
ATOM    992  CA  GLY A  70      45.049  87.987  51.605  1.00  1.00           C
ATOM    993  C   GLY A  70      45.289  88.526  50.197  1.00  1.00           C
ATOM    994  O   GLY A  70      45.923  89.569  50.064  1.00  1.00           O
ATOM    995  H   GLY A  70      43.574  86.461  51.413  1.00  1.00           H
ATOM    996  HA2 GLY A  70      44.313  88.632  52.085  1.00  1.00           H
ATOM    997  HA3 GLY A  70      45.982  88.067  52.164  1.00  1.00           H
ATOM    998  N   ALA A  71      44.802  87.837  49.158  1.00  1.00           N
ATOM    999  CA  ALA A  71      44.813  88.289  47.774  1.00  1.00           C
ATOM   1000  C   ALA A  71      43.647  87.631  47.042  1.00  1.00           C
```

```
ATOM 1001 O ALA A 71 43.334 86.469 47.305 1.00 1.00 O
ATOM 1002 CB ALA A 71 46.163 88.006 47.097 1.00 1.00 C
ATOM 1003 H ALA A 71 44.305 86.975 49.337 1.00 1.00 H
ATOM 1004 HA ALA A 71 44.655 89.369 47.765 1.00 1.00 H
ATOM 1005 HB1 ALA A 71 46.969 88.478 47.660 1.00 1.00 H
ATOM 1006 HB2 ALA A 71 46.334 86.930 47.045 1.00 1.00 H
ATOM 1007 HB3 ALA A 71 46.156 88.414 46.085 1.00 1.00 H
ATOM 1008 N GLU A 72 43.054 88.375 46.110 1.00 1.00 N
ATOM 1009 CA GLU A 72 42.061 87.982 45.113 1.00 1.00 C
ATOM 1010 C GLU A 72 42.627 86.988 44.075 1.00 1.00 C
ATOM 1011 O GLU A 72 41.879 86.201 43.486 1.00 1.00 O
ATOM 1012 CB GLU A 72 41.566 89.294 44.434 1.00 1.00 C
ATOM 1013 CG GLU A 72 42.576 90.044 43.545 1.00 1.00 C
ATOM 1014 CD GLU A 72 43.860 90.343 44.300 1.00 1.00 C
ATOM 1015 OE1 GLU A 72 44.889 89.731 43.946 1.00 1.00 O
ATOM 1016 OE2 GLU A 72 43.774 91.092 45.295 1.00 1.00 O1-
ATOM 1017 H GLU A 72 43.377 89.344 46.060 1.00 1.00 H
ATOM 1018 HA GLU A 72 41.223 87.501 45.618 1.00 1.00 H
ATOM 1019 HB2 GLU A 72 40.706 89.043 43.812 1.00 1.00 H
ATOM 1020 HB3 GLU A 72 41.207 89.971 45.211 1.00 1.00 H
ATOM 1021 HG2 GLU A 72 42.802 89.446 42.662 1.00 1.00 H
ATOM 1022 HG3 GLU A 72 42.132 90.984 43.217 1.00 1.00 H
ATOM 1023 N TYR A 73 43.945 87.032 43.831 1.00 1.00 N
ATOM 1024 CA TYR A 73 44.687 86.094 43.009 1.00 1.00 C
ATOM 1025 C TYR A 73 44.615 84.695 43.636 1.00 1.00 C
ATOM 1026 O TYR A 73 44.732 84.526 44.850 1.00 1.00 O
ATOM 1027 CB TYR A 73 46.155 86.544 42.863 1.00 1.00 C
ATOM 1028 CG TYR A 73 46.946 85.620 41.951 1.00 1.00 C
ATOM 1029 CD1 TYR A 73 46.906 85.812 40.557 1.00 1.00 C
ATOM 1030 CD2 TYR A 73 47.666 84.531 42.485 1.00 1.00 C
ATOM 1031 CE1 TYR A 73 47.571 84.917 39.698 1.00 1.00 C
ATOM 1032 CE2 TYR A 73 48.325 83.634 41.623 1.00 1.00 C
ATOM 1033 CZ TYR A 73 48.276 83.825 40.231 1.00 1.00 C
ATOM 1034 OH TYR A 73 48.907 82.952 39.397 1.00 1.00 O
ATOM 1035 H TYR A 73 44.451 87.833 44.208 1.00 1.00 H
ATOM 1036 HA TYR A 73 44.230 86.068 42.019 1.00 1.00 H
ATOM 1037 HB2 TYR A 73 46.179 87.554 42.452 1.00 1.00 H
ATOM 1038 HB3 TYR A 73 46.625 86.572 43.848 1.00 1.00 H
ATOM 1039 HD1 TYR A 73 46.355 86.645 40.142 1.00 1.00 H
ATOM 1040 HD2 TYR A 73 47.701 84.373 43.556 1.00 1.00 H
ATOM 1041 HE1 TYR A 73 47.530 85.072 38.630 1.00 1.00 H
ATOM 1042 HE2 TYR A 73 48.864 82.789 42.026 1.00 1.00 H
ATOM 1043 HH TYR A 73 48.843 83.222 38.478 1.00 1.00 H
ATOM 1044 N LYS A 74 44.432 83.674 42.796 1.00 1.00 N
ATOM 1045 CA LYS A 74 44.214 82.303 43.226 1.00 1.00 C
ATOM 1046 C LYS A 74 45.551 81.650 43.593 1.00 1.00 C
ATOM 1047 O LYS A 74 46.202 81.005 42.770 1.00 1.00 O
ATOM 1048 CB LYS A 74 43.444 81.555 42.124 1.00 1.00 C
ATOM 1049 CG LYS A 74 42.014 82.119 42.066 1.00 1.00 C
ATOM 1050 CD LYS A 74 41.092 81.262 41.201 1.00 1.00 C
```

ATOM 1051 CE LYS A 74 39.689 81.870 41.250 1.00 1.00 C
ATOM 1052 NZ LYS A 74 38.710 81.024 40.549 1.00 1.00 N1+
ATOM 1053 H LYS A 74 44.392 83.873 41.809 1.00 1.00 H
ATOM 1054 HA LYS A 74 43.592 82.310 44.120 1.00 1.00 H
ATOM 1055 HB2 LYS A 74 43.940 81.667 41.158 1.00 1.00 H
ATOM 1056 HB3 LYS A 74 43.401 80.496 42.385 1.00 1.00 H
ATOM 1057 HG2 LYS A 74 41.603 82.154 43.078 1.00 1.00 H
ATOM 1058 HG3 LYS A 74 42.037 83.137 41.672 1.00 1.00 H
ATOM 1059 HD2 LYS A 74 41.459 81.244 40.173 1.00 1.00 H
ATOM 1060 HD3 LYS A 74 41.068 80.246 41.601 1.00 1.00 H
ATOM 1061 HE2 LYS A 74 39.386 81.978 42.293 1.00 1.00 H
ATOM 1062 HE3 LYS A 74 39.713 82.860 40.790 1.00 1.00 H
ATOM 1063 HZ1 LYS A 74 37.798 81.452 40.607 1.00 1.00 H
ATOM 1064 HZ2 LYS A 74 38.979 80.929 39.580 1.00 1.00 H
ATOM 1065 HZ3 LYS A 74 38.683 80.112 40.983 1.00 1.00 H
ATOM 1066 N GLN A 75 45.918 81.898 44.855 1.00 1.00 N
ATOM 1067 CA GLN A 75 47.077 81.467 45.621 1.00 1.00 C
ATOM 1068 C GLN A 75 47.497 80.006 45.396 1.00 1.00 C
ATOM 1069 O GLN A 75 46.664 79.112 45.248 1.00 1.00 O
ATOM 1070 CB GLN A 75 46.818 81.744 47.117 1.00 1.00 C
ATOM 1071 CG GLN A 75 46.504 83.184 47.556 1.00 1.00 C
ATOM 1072 CD GLN A 75 47.717 84.090 47.587 1.00 1.00 C
ATOM 1073 NE2 GLN A 75 47.800 84.980 48.558 1.00 1.00 N
ATOM 1074 OE1 GLN A 75 48.603 83.997 46.753 1.00 1.00 O
ATOM 1075 H GLN A 75 45.351 82.614 45.297 1.00 1.00 H
ATOM 1076 HA GLN A 75 47.911 82.093 45.305 1.00 1.00 H
ATOM 1077 HB2 GLN A 75 45.989 81.110 47.436 1.00 1.00 H
ATOM 1078 HB3 GLN A 75 47.701 81.428 47.664 1.00 1.00 H
ATOM 1079 HG2 GLN A 75 45.757 83.648 46.923 1.00 1.00 H
ATOM 1080 HG3 GLN A 75 46.095 83.141 48.560 1.00 1.00 H
ATOM 1081 HE21 GLN A 75 47.084 85.059 49.260 1.00 1.00 H
ATOM 1082 HE22 GLN A 75 48.610 85.575 48.557 1.00 1.00 H
ATOM 1083 N LYS A 76 48.812 79.777 45.412 1.00 1.00 N
ATOM 1084 CA LYS A 76 49.441 78.470 45.316 1.00 1.00 C
ATOM 1085 C LYS A 76 49.875 78.054 46.723 1.00 1.00 C
ATOM 1086 O LYS A 76 50.508 78.839 47.429 1.00 1.00 O
ATOM 1087 CB LYS A 76 50.687 78.605 44.416 1.00 1.00 C
ATOM 1088 CG LYS A 76 50.270 78.710 42.939 1.00 1.00 C
ATOM 1089 CD LYS A 76 49.912 77.322 42.396 1.00 1.00 C
ATOM 1090 CE LYS A 76 48.859 77.364 41.290 1.00 1.00 C
ATOM 1091 NZ LYS A 76 48.536 75.995 40.853 1.00 1.00 N1+
ATOM 1092 H LYS A 76 49.412 80.562 45.618 1.00 1.00 H
ATOM 1093 HA LYS A 76 48.747 77.732 44.915 1.00 1.00 H
ATOM 1094 HB2 LYS A 76 51.248 79.498 44.698 1.00 1.00 H
ATOM 1095 HB3 LYS A 76 51.340 77.740 44.548 1.00 1.00 H
ATOM 1096 HG2 LYS A 76 49.422 79.389 42.838 1.00 1.00 H
ATOM 1097 HG3 LYS A 76 51.100 79.111 42.357 1.00 1.00 H
ATOM 1098 HD2 LYS A 76 50.826 76.879 42.010 1.00 1.00 H
ATOM 1099 HD3 LYS A 76 49.542 76.677 43.193 1.00 1.00 H
ATOM 1100 HE2 LYS A 76 47.959 77.848 41.670 1.00 1.00 H

ATOM 1101 HE3 LYS A 76 49.245 77.936 40.445 1.00 1.00 H
ATOM 1102 HZ1 LYS A 76 49.396 75.570 40.489 1.00 1.00 H
ATOM 1103 HZ2 LYS A 76 48.256 75.433 41.644 1.00 1.00 H
ATOM 1104 HZ3 LYS A 76 47.839 75.994 40.126 1.00 1.00 H
ATOM 1105 N GLN A 77 49.526 76.828 47.120 1.00 1.00 N
ATOM 1106 CA GLN A 77 49.895 76.237 48.400 1.00 1.00 C
ATOM 1107 C GLN A 77 51.268 75.555 48.271 1.00 1.00 C
ATOM 1108 O GLN A 77 51.686 75.186 47.171 1.00 1.00 O
ATOM 1109 CB GLN A 77 48.864 75.145 48.756 1.00 1.00 C
ATOM 1110 CG GLN A 77 47.418 75.649 48.803 1.00 1.00 C
ATOM 1111 CD GLN A 77 46.571 74.751 49.682 1.00 1.00 C
ATOM 1112 NE2 GLN A 77 45.546 75.307 50.296 1.00 1.00 N
ATOM 1113 OE1 GLN A 77 46.853 73.572 49.862 1.00 1.00 O
ATOM 1114 H GLN A 77 49.024 76.254 46.462 1.00 1.00 H
ATOM 1115 HA GLN A 77 49.921 76.998 49.180 1.00 1.00 H
ATOM 1116 HB2 GLN A 77 48.928 74.326 48.037 1.00 1.00 H
ATOM 1117 HB3 GLN A 77 49.138 74.756 49.738 1.00 1.00 H
ATOM 1118 HG2 GLN A 77 47.412 76.652 49.224 1.00 1.00 H
ATOM 1119 HG3 GLN A 77 46.986 75.687 47.804 1.00 1.00 H
ATOM 1120 HE21 GLN A 77 45.235 76.245 50.048 1.00 1.00 H
ATOM 1121 HE22 GLN A 77 45.128 74.773 51.059 1.00 1.00 H
ATOM 1122 N ILE A 78 51.953 75.372 49.404 1.00 1.00 N
ATOM 1123 CA ILE A 78 53.234 74.672 49.527 1.00 1.00 C
ATOM 1124 C ILE A 78 53.012 73.453 50.447 1.00 1.00 C
ATOM 1125 O ILE A 78 52.208 73.533 51.380 1.00 1.00 O
ATOM 1126 CB ILE A 78 54.319 75.636 50.102 1.00 1.00 C
ATOM 1127 CG1 ILE A 78 54.358 77.056 49.483 1.00 1.00 C
ATOM 1128 CG2 ILE A 78 55.710 74.981 50.007 1.00 1.00 C
ATOM 1129 CD1 ILE A 78 54.669 77.129 47.984 1.00 1.00 C
ATOM 1130 H ILE A 78 51.516 75.706 50.261 1.00 1.00 H
ATOM 1131 HA ILE A 78 53.554 74.319 48.546 1.00 1.00 H
ATOM 1132 HB ILE A 78 54.098 75.776 51.162 1.00 1.00 H
ATOM 1133 HG12 ILE A 78 53.402 77.550 49.659 1.00 1.00 H
ATOM 1134 HG13 ILE A 78 55.113 77.640 50.012 1.00 1.00 H
ATOM 1135 HG21 ILE A 78 55.930 74.694 48.979 1.00 1.00 H
ATOM 1136 HG22 ILE A 78 56.477 75.674 50.354 1.00 1.00 H
ATOM 1137 HG23 ILE A 78 55.755 74.093 50.636 1.00 1.00 H
ATOM 1138 HD11 ILE A 78 55.656 76.714 47.779 1.00 1.00 H
ATOM 1139 HD12 ILE A 78 53.913 76.588 47.420 1.00 1.00 H
ATOM 1140 HD13 ILE A 78 54.658 78.172 47.667 1.00 1.00 H
ATOM 1141 N ASN A 79 53.717 72.343 50.201 1.00 1.00 N
ATOM 1142 CA ASN A 79 53.619 71.070 50.925 1.00 1.00 C
ATOM 1143 C ASN A 79 55.019 70.451 51.066 1.00 1.00 C
ATOM 1144 O ASN A 79 55.775 70.446 50.096 1.00 1.00 O
ATOM 1145 CB ASN A 79 52.638 70.149 50.167 1.00 1.00 C
ATOM 1146 CG ASN A 79 52.407 68.819 50.868 1.00 1.00 C
ATOM 1147 ND2 ASN A 79 52.555 67.694 50.186 1.00 1.00 N
ATOM 1148 OD1 ASN A 79 52.064 68.786 52.040 1.00 1.00 O
ATOM 1149 H ASN A 79 54.300 72.339 49.363 1.00 1.00 H
ATOM 1150 HA ASN A 79 53.225 71.253 51.925 1.00 1.00 H

ATOM 1151 HB2 ASN A 79 51.677 70.648 50.051 1.00 1.00 H
ATOM 1152 HB3 ASN A 79 53.042 69.963 49.178 1.00 1.00 H
ATOM 1153 HD21 ASN A 79 52.931 67.657 49.225 1.00 1.00 H
ATOM 1154 HD22 ASN A 79 52.291 66.851 50.661 1.00 1.00 H
ATOM 1155 N GLY A 80 55.352 69.917 52.245 1.00 1.00 N
ATOM 1156 CA GLY A 80 56.483 69.037 52.510 1.00 1.00 C
ATOM 1157 C GLY A 80 55.962 67.784 53.219 1.00 1.00 C
ATOM 1158 O GLY A 80 55.263 67.887 54.230 1.00 1.00 O
ATOM 1159 H GLY A 80 54.713 70.059 53.033 1.00 1.00 H
ATOM 1160 HA2 GLY A 80 56.972 68.758 51.578 1.00 1.00 H
ATOM 1161 HA3 GLY A 80 57.210 69.533 53.150 1.00 1.00 H
ATOM 1162 N ASN A 81 56.292 66.602 52.693 1.00 1.00 N
ATOM 1163 CA ASN A 81 55.796 65.297 53.131 1.00 1.00 C
ATOM 1164 C ASN A 81 57.011 64.382 53.344 1.00 1.00 C
ATOM 1165 O ASN A 81 57.674 64.023 52.369 1.00 1.00 O
ATOM 1166 CB ASN A 81 54.835 64.770 52.041 1.00 1.00 C
ATOM 1167 CG ASN A 81 54.286 63.377 52.315 1.00 1.00 C
ATOM 1168 ND2 ASN A 81 55.132 62.362 52.236 1.00 1.00 N
ATOM 1169 OD1 ASN A 81 53.095 63.199 52.555 1.00 1.00 O
ATOM 1170 H ASN A 81 56.923 66.610 51.887 1.00 1.00 H
ATOM 1171 HA ASN A 81 55.251 65.392 54.071 1.00 1.00 H
ATOM 1172 HB2 ASN A 81 53.995 65.458 51.950 1.00 1.00 H
ATOM 1173 HB3 ASN A 81 55.356 64.748 51.082 1.00 1.00 H
ATOM 1174 HD21 ASN A 81 56.104 62.537 52.014 1.00 1.00 H
ATOM 1175 HD22 ASN A 81 54.791 61.429 52.408 1.00 1.00 H
ATOM 1176 N PHE A 82 57.330 64.031 54.596 1.00 1.00 N
ATOM 1177 CA PHE A 82 58.554 63.341 54.998 1.00 1.00 C
ATOM 1178 C PHE A 82 58.174 62.075 55.789 1.00 1.00 C
ATOM 1179 O PHE A 82 57.828 62.144 56.968 1.00 1.00 O
ATOM 1180 CB PHE A 82 59.432 64.307 55.834 1.00 1.00 C
ATOM 1181 CG PHE A 82 59.548 65.731 55.300 1.00 1.00 C
ATOM 1182 CD1 PHE A 82 59.899 65.973 53.957 1.00 1.00 C
ATOM 1183 CD2 PHE A 82 59.282 66.825 56.147 1.00 1.00 C
ATOM 1184 CE1 PHE A 82 59.979 67.287 53.464 1.00 1.00 C
ATOM 1185 CE2 PHE A 82 59.381 68.140 55.662 1.00 1.00 C
ATOM 1186 CZ PHE A 82 59.728 68.373 54.319 1.00 1.00 C
ATOM 1187 H PHE A 82 56.746 64.357 55.370 1.00 1.00 H
ATOM 1188 HA PHE A 82 59.119 63.048 54.115 1.00 1.00 H
ATOM 1189 HB2 PHE A 82 59.033 64.367 56.841 1.00 1.00 H
ATOM 1190 HB3 PHE A 82 60.428 63.877 55.915 1.00 1.00 H
ATOM 1191 HD1 PHE A 82 60.093 65.147 53.295 1.00 1.00 H
ATOM 1192 HD2 PHE A 82 59.001 66.659 57.177 1.00 1.00 H
ATOM 1193 HE1 PHE A 82 60.230 67.464 52.428 1.00 1.00 H
ATOM 1194 HE2 PHE A 82 59.190 68.973 56.323 1.00 1.00 H
ATOM 1195 HZ PHE A 82 59.803 69.383 53.943 1.00 1.00 H
ATOM 1196 N TYR A 83 58.202 60.908 55.145 1.00 1.00 N
ATOM 1197 CA TYR A 83 57.874 59.613 55.745 1.00 1.00 C
ATOM 1198 C TYR A 83 59.186 58.866 56.025 1.00 1.00 C
ATOM 1199 O TYR A 83 60.082 58.877 55.179 1.00 1.00 O
ATOM 1200 CB TYR A 83 57.086 58.767 54.715 1.00 1.00 C

ATOM 1201 CG TYR A 83 55.706 59.286 54.327 1.00 1.00 C
ATOM 1202 CD1 TYR A 83 55.080 58.769 53.175 1.00 1.00 C
ATOM 1203 CD2 TYR A 83 55.038 60.271 55.089 1.00 1.00 C
ATOM 1204 CE1 TYR A 83 53.811 59.235 52.783 1.00 1.00 C
ATOM 1205 CE2 TYR A 83 53.783 60.749 54.682 1.00 1.00 C
ATOM 1206 CZ TYR A 83 53.168 60.231 53.534 1.00 1.00 C
ATOM 1207 OH TYR A 83 52.002 60.775 53.099 1.00 1.00 O
ATOM 1208 H TYR A 83 58.593 60.897 54.200 1.00 1.00 H
ATOM 1209 HA TYR A 83 57.305 59.721 56.669 1.00 1.00 H
ATOM 1210 HB2 TYR A 83 57.682 58.676 53.807 1.00 1.00 H
ATOM 1211 HB3 TYR A 83 56.955 57.765 55.125 1.00 1.00 H
ATOM 1212 HD1 TYR A 83 55.581 58.018 52.578 1.00 1.00 H
ATOM 1213 HD2 TYR A 83 55.479 60.689 55.982 1.00 1.00 H
ATOM 1214 HE1 TYR A 83 53.339 58.851 51.890 1.00 1.00 H
ATOM 1215 HE2 TYR A 83 53.298 61.531 55.249 1.00 1.00 H
ATOM 1216 HH TYR A 83 52.106 61.739 53.018 1.00 1.00 H
ATOM 1217 N VAL A 84 59.299 58.180 57.168 1.00 1.00 N
ATOM 1218 CA VAL A 84 60.416 57.305 57.509 1.00 1.00 C
ATOM 1219 C VAL A 84 59.839 56.004 58.079 1.00 1.00 C
ATOM 1220 O VAL A 84 59.084 56.024 59.050 1.00 1.00 O
ATOM 1221 CB VAL A 84 61.467 58.009 58.418 1.00 1.00 C
ATOM 1222 CG1 VAL A 84 62.834 57.316 58.327 1.00 1.00 C
ATOM 1223 CG2 VAL A 84 61.660 59.511 58.174 1.00 1.00 C
ATOM 1224 H VAL A 84 58.539 58.249 57.851 1.00 1.00 H
ATOM 1225 HA VAL A 84 60.921 57.050 56.581 1.00 1.00 H
ATOM 1226 HB VAL A 84 61.130 57.903 59.446 1.00 1.00 H
ATOM 1227 HG11 VAL A 84 62.715 56.236 58.415 1.00 1.00 H
ATOM 1228 HG12 VAL A 84 63.310 57.541 57.373 1.00 1.00 H
ATOM 1229 HG13 VAL A 84 63.462 57.653 59.151 1.00 1.00 H
ATOM 1230 HG21 VAL A 84 61.971 59.688 57.145 1.00 1.00 H
ATOM 1231 HG22 VAL A 84 60.727 60.043 58.360 1.00 1.00 H
ATOM 1232 HG23 VAL A 84 62.404 59.908 58.863 1.00 1.00 H
ATOM 1233 N THR A 85 60.190 54.869 57.474 1.00 1.00 N
ATOM 1234 CA THR A 85 59.890 53.533 57.985 1.00 1.00 C
ATOM 1235 C THR A 85 61.168 53.107 58.712 1.00 1.00 C
ATOM 1236 O THR A 85 62.246 53.184 58.123 1.00 1.00 O
ATOM 1237 CB THR A 85 59.643 52.608 56.773 1.00 1.00 C
ATOM 1238 CG2 THR A 85 59.242 51.204 57.228 1.00 1.00 C
ATOM 1239 OG1 THR A 85 58.609 53.106 55.955 1.00 1.00 O
ATOM 1240 H THR A 85 60.875 54.943 56.718 1.00 1.00 H
ATOM 1241 HA THR A 85 59.028 53.536 58.654 1.00 1.00 H
ATOM 1242 HB THR A 85 60.543 52.545 56.163 1.00 1.00 H
ATOM 1243 HG1 THR A 85 58.929 53.918 55.557 1.00 1.00 H
ATOM 1244 HG21 THR A 85 59.010 50.591 56.357 1.00 1.00 H
ATOM 1245 HG22 THR A 85 60.062 50.736 57.775 1.00 1.00 H
ATOM 1246 HG23 THR A 85 58.363 51.258 57.872 1.00 1.00 H
ATOM 1247 N SER A 86 61.058 52.748 59.991 1.00 1.00 N
ATOM 1248 CA SER A 86 62.166 52.508 60.901 1.00 1.00 C
ATOM 1249 C SER A 86 62.024 51.106 61.508 1.00 1.00 C
ATOM 1250 O SER A 86 60.999 50.811 62.123 1.00 1.00 O

```
ATOM   1251  CB  SER A  86      62.117  53.603  61.992  1.00  1.00           C
ATOM   1252  OG  SER A  86      62.189  54.856  61.349  1.00  1.00           O
ATOM   1253  H   SER A  86      60.128  52.712  60.412  1.00  1.00           H
ATOM   1254  HA  SER A  86      63.115  52.576  60.377  1.00  1.00           H
ATOM   1255  HB2 SER A  86      61.186  53.531  62.554  1.00  1.00           H
ATOM   1256  HB3 SER A  86      62.965  53.483  62.664  1.00  1.00           H
ATOM   1257  HG  SER A  86      61.546  54.823  60.628  1.00  1.00           H
ATOM   1258  N   ASP A  87      63.055  50.269  61.381  1.00  1.00           N
ATOM   1259  CA  ASP A  87      63.212  48.982  62.060  1.00  1.00           C
ATOM   1260  C   ASP A  87      63.707  49.307  63.476  1.00  1.00           C
ATOM   1261  O   ASP A  87      64.685  50.044  63.627  1.00  1.00           O
ATOM   1262  CB  ASP A  87      64.297  48.107  61.374  1.00  1.00           C
ATOM   1263  CG  ASP A  87      63.779  47.327  60.143  1.00  1.00           C
ATOM   1264  OD1 ASP A  87      62.945  47.882  59.389  1.00  1.00           O
ATOM   1265  OD2 ASP A  87      64.226  46.168  59.974  1.00  1.00           O1-
ATOM   1266  H   ASP A  87      63.885  50.619  60.902  1.00  1.00           H
ATOM   1267  HA  ASP A  87      62.266  48.449  62.118  1.00  1.00           H
ATOM   1268  HB2 ASP A  87      65.144  48.729  61.080  1.00  1.00           H
ATOM   1269  HB3 ASP A  87      64.659  47.380  62.104  1.00  1.00           H
ATOM   1270  N   LEU A  88      63.021  48.801  64.507  1.00  1.00           N
ATOM   1271  CA  LEU A  88      63.305  49.049  65.917  1.00  1.00           C
ATOM   1272  C   LEU A  88      63.873  47.762  66.507  1.00  1.00           C
ATOM   1273  O   LEU A  88      63.229  46.714  66.477  1.00  1.00           O
ATOM   1274  CB  LEU A  88      61.991  49.496  66.620  1.00  1.00           C
ATOM   1275  CG  LEU A  88      61.951  49.682  68.157  1.00  1.00           C
ATOM   1276  CD1 LEU A  88      61.803  48.369  68.937  1.00  1.00           C
ATOM   1277  CD2 LEU A  88      63.132  50.459  68.743  1.00  1.00           C
ATOM   1278  H   LEU A  88      62.253  48.156  64.317  1.00  1.00           H
ATOM   1279  HA  LEU A  88      64.053  49.832  66.016  1.00  1.00           H
ATOM   1280  HB2 LEU A  88      61.698  50.446  66.173  1.00  1.00           H
ATOM   1281  HB3 LEU A  88      61.203  48.792  66.361  1.00  1.00           H
ATOM   1282  HG  LEU A  88      61.052  50.257  68.376  1.00  1.00           H
ATOM   1283 HD11 LEU A  88      60.963  47.798  68.543  1.00  1.00           H
ATOM   1284 HD12 LEU A  88      62.710  47.772  68.883  1.00  1.00           H
ATOM   1285 HD13 LEU A  88      61.596  48.593  69.983  1.00  1.00           H
ATOM   1286 HD21 LEU A  88      62.998  50.568  69.818  1.00  1.00           H
ATOM   1287 HD22 LEU A  88      64.071  49.940  68.559  1.00  1.00           H
ATOM   1288 HD23 LEU A  88      63.168  51.453  68.303  1.00  1.00           H
ATOM   1289  N   ILE A  89      65.069  47.863  67.082  1.00  1.00           N
ATOM   1290  CA  ILE A  89      65.770  46.807  67.789  1.00  1.00           C
ATOM   1291  C   ILE A  89      66.111  47.325  69.190  1.00  1.00           C
ATOM   1292  O   ILE A  89      66.298  48.525  69.401  1.00  1.00           O
ATOM   1293  CB  ILE A  89      67.026  46.358  66.988  1.00  1.00           C
ATOM   1294  CG1 ILE A  89      68.053  47.504  66.808  1.00  1.00           C
ATOM   1295  CG2 ILE A  89      66.604  45.754  65.636  1.00  1.00           C
ATOM   1296  CD1 ILE A  89      69.364  47.072  66.142  1.00  1.00           C
ATOM   1297  H   ILE A  89      65.485  48.794  67.107  1.00  1.00           H
ATOM   1298  HA  ILE A  89      65.108  45.947  67.904  1.00  1.00           H
ATOM   1299  HB  ILE A  89      67.508  45.566  67.563  1.00  1.00           H
ATOM   1300 HG12 ILE A  89      67.611  48.307  66.217  1.00  1.00           H
```

ATOM	1301	HG13	ILE	A	89	68.313	47.907	67.786	1.00	1.00	H
ATOM	1302	HG21	ILE	A	89	67.454	45.278	65.148	1.00	1.00	H
ATOM	1303	HG22	ILE	A	89	65.844	44.992	65.798	1.00	1.00	H
ATOM	1304	HG23	ILE	A	89	66.202	46.528	64.980	1.00	1.00	H
ATOM	1305	HD11	ILE	A	89	69.799	46.232	66.684	1.00	1.00	H
ATOM	1306	HD12	ILE	A	89	69.189	46.788	65.104	1.00	1.00	H
ATOM	1307	HD13	ILE	A	89	70.067	47.906	66.156	1.00	1.00	H
ATOM	1308	N	THR	A	90	66.233	46.412	70.151	1.00	1.00	N
ATOM	1309	CA	THR	A	90	66.679	46.696	71.504	1.00	1.00	C
ATOM	1310	C	THR	A	90	67.781	45.695	71.890	1.00	1.00	C
ATOM	1311	O	THR	A	90	68.244	44.897	71.071	1.00	1.00	O
ATOM	1312	CB	THR	A	90	65.469	46.833	72.466	1.00	1.00	C
ATOM	1313	CG2	THR	A	90	64.420	47.869	72.048	1.00	1.00	C
ATOM	1314	OG1	THR	A	90	64.812	45.594	72.559	1.00	1.00	O
ATOM	1315	H	THR	A	90	66.028	45.448	69.918	1.00	1.00	H
ATOM	1316	HA	THR	A	90	67.168	47.671	71.508	1.00	1.00	H
ATOM	1317	HB	THR	A	90	65.852	47.098	73.451	1.00	1.00	H
ATOM	1318	HG1	THR	A	90	64.436	45.375	71.681	1.00	1.00	H
ATOM	1319	HG21	THR	A	90	63.728	48.035	72.873	1.00	1.00	H
ATOM	1320	HG22	THR	A	90	64.906	48.808	71.793	1.00	1.00	H
ATOM	1321	HG23	THR	A	90	63.862	47.519	71.179	1.00	1.00	H
ATOM	1322	N	LYS	A	91	68.262	45.777	73.129	1.00	1.00	N
ATOM	1323	CA	LYS	A	91	69.406	45.055	73.661	1.00	1.00	C
ATOM	1324	C	LYS	A	91	69.168	44.825	75.153	1.00	1.00	C
ATOM	1325	O	LYS	A	91	68.396	45.567	75.765	1.00	1.00	O
ATOM	1326	CB	LYS	A	91	70.707	45.846	73.364	1.00	1.00	C
ATOM	1327	CG	LYS	A	91	70.720	47.258	73.985	1.00	1.00	C
ATOM	1328	CD	LYS	A	91	72.141	47.755	74.279	1.00	1.00	C
ATOM	1329	CE	LYS	A	91	72.069	49.112	74.990	1.00	1.00	C
ATOM	1330	NZ	LYS	A	91	73.396	49.578	75.430	1.00	1.00	N1+
ATOM	1331	H	LYS	A	91	67.787	46.378	73.790	1.00	1.00	H
ATOM	1332	HA	LYS	A	91	69.477	44.081	73.175	1.00	1.00	H
ATOM	1333	HB2	LYS	A	91	71.540	45.269	73.768	1.00	1.00	H
ATOM	1334	HB3	LYS	A	91	70.852	45.926	72.286	1.00	1.00	H
ATOM	1335	HG2	LYS	A	91	70.219	47.951	73.306	1.00	1.00	H
ATOM	1336	HG3	LYS	A	91	70.173	47.254	74.926	1.00	1.00	H
ATOM	1337	HD2	LYS	A	91	72.641	47.036	74.931	1.00	1.00	H
ATOM	1338	HD3	LYS	A	91	72.700	47.847	73.347	1.00	1.00	H
ATOM	1339	HE2	LYS	A	91	71.631	49.847	74.313	1.00	1.00	H
ATOM	1340	HE3	LYS	A	91	71.423	49.018	75.863	1.00	1.00	H
ATOM	1341	HZ1	LYS	A	91	73.986	49.724	74.625	1.00	1.00	H
ATOM	1342	HZ2	LYS	A	91	73.294	50.450	75.934	1.00	1.00	H
ATOM	1343	HZ3	LYS	A	91	73.817	48.890	76.041	1.00	1.00	H
ATOM	1344	N	ASN	A	92	69.895	43.879	75.758	1.00	1.00	N
ATOM	1345	CA	ASN	A	92	69.934	43.671	77.209	1.00	1.00	C
ATOM	1346	C	ASN	A	92	70.560	44.923	77.846	1.00	1.00	C
ATOM	1347	O	ASN	A	92	71.696	45.282	77.530	1.00	1.00	O
ATOM	1348	CB	ASN	A	92	70.765	42.417	77.574	1.00	1.00	C
ATOM	1349	CG	ASN	A	92	70.446	41.228	76.679	1.00	1.00	C
ATOM	1350	ND2	ASN	A	92	69.591	40.322	77.116	1.00	1.00	N

ATOM 1351 OD1 ASN A 92 70.949 41.133 75.568 1.00 1.00 O
ATOM 1352 H ASN A 92 70.483 43.278 75.193 1.00 1.00 H
ATOM 1353 HA ASN A 92 68.914 43.536 77.572 1.00 1.00 H
ATOM 1354 HB2 ASN A 92 71.827 42.635 77.452 1.00 1.00 H
ATOM 1355 HB3 ASN A 92 70.591 42.155 78.617 1.00 1.00 H
ATOM 1356 HD21 ASN A 92 69.148 40.420 78.015 1.00 1.00 H
ATOM 1357 HD22 ASN A 92 69.383 39.551 76.503 1.00 1.00 H
ATOM 1358 N TYR A 93 69.768 45.650 78.637 1.00 1.00 N
ATOM 1359 CA TYR A 93 70.102 46.947 79.209 1.00 1.00 C
ATOM 1360 C TYR A 93 69.205 47.154 80.433 1.00 1.00 C
ATOM 1361 O TYR A 93 69.530 46.680 81.517 1.00 1.00 O
ATOM 1362 CB TYR A 93 70.036 48.048 78.120 1.00 1.00 C
ATOM 1363 CG TYR A 93 70.808 49.294 78.501 1.00 1.00 C
ATOM 1364 CD1 TYR A 93 72.217 49.249 78.536 1.00 1.00 C
ATOM 1365 CD2 TYR A 93 70.136 50.485 78.837 1.00 1.00 C
ATOM 1366 CE1 TYR A 93 72.952 50.396 78.889 1.00 1.00 C
ATOM 1367 CE2 TYR A 93 70.873 51.628 79.200 1.00 1.00 C
ATOM 1368 CZ TYR A 93 72.278 51.582 79.221 1.00 1.00 C
ATOM 1369 OH TYR A 93 72.998 52.683 79.569 1.00 1.00 O
ATOM 1370 H TYR A 93 68.829 45.309 78.785 1.00 1.00 H
ATOM 1371 HA TYR A 93 71.129 46.894 79.572 1.00 1.00 H
ATOM 1372 HB2 TYR A 93 70.489 47.661 77.209 1.00 1.00 H
ATOM 1373 HB3 TYR A 93 69.003 48.295 77.874 1.00 1.00 H
ATOM 1374 HD1 TYR A 93 72.730 48.323 78.316 1.00 1.00 H
ATOM 1375 HD2 TYR A 93 69.055 50.524 78.834 1.00 1.00 H
ATOM 1376 HE1 TYR A 93 74.032 50.365 78.935 1.00 1.00 H
ATOM 1377 HE2 TYR A 93 70.352 52.537 79.466 1.00 1.00 H
ATOM 1378 HH TYR A 93 72.449 53.407 79.879 1.00 1.00 H
ATOM 1379 N ASP A 94 68.038 47.773 80.236 1.00 1.00 N
ATOM 1380 CA ASP A 94 66.951 47.905 81.198 1.00 1.00 C
ATOM 1381 C ASP A 94 65.625 47.822 80.420 1.00 1.00 C
ATOM 1382 O ASP A 94 65.591 48.027 79.202 1.00 1.00 O
ATOM 1383 CB ASP A 94 67.162 49.106 82.162 1.00 1.00 C
ATOM 1384 CG ASP A 94 65.928 49.479 82.988 1.00 1.00 C
ATOM 1385 OD1 ASP A 94 65.199 48.576 83.466 1.00 1.00 O
ATOM 1386 OD2 ASP A 94 65.656 50.694 83.082 1.00 1.00 O1-
ATOM 1387 H ASP A 94 67.823 48.094 79.302 1.00 1.00 H
ATOM 1388 HA ASP A 94 66.971 47.016 81.831 1.00 1.00 H
ATOM 1389 HB2 ASP A 94 67.987 48.877 82.838 1.00 1.00 H
ATOM 1390 HB3 ASP A 94 67.453 49.972 81.564 1.00 1.00 H
ATOM 1391 N SER A 95 64.555 47.472 81.129 1.00 1.00 N
ATOM 1392 CA SER A 95 63.183 47.419 80.663 1.00 1.00 C
ATOM 1393 C SER A 95 62.449 48.755 80.882 1.00 1.00 C
ATOM 1394 O SER A 95 61.466 48.997 80.177 1.00 1.00 O
ATOM 1395 CB SER A 95 62.456 46.373 81.531 1.00 1.00 C
ATOM 1396 OG SER A 95 62.962 45.083 81.259 1.00 1.00 O
ATOM 1397 H SER A 95 64.709 47.447 82.140 1.00 1.00 H
ATOM 1398 HA SER A 95 63.139 47.134 79.611 1.00 1.00 H
ATOM 1399 HB2 SER A 95 62.585 46.607 82.589 1.00 1.00 H
ATOM 1400 HB3 SER A 95 61.392 46.385 81.293 1.00 1.00 H

ATOM	1401	HG	SER	A	95	63.891	45.056	81.507	1.00	1.00	H
ATOM	1402	N	LYS	A	96	62.857	49.579	81.861	1.00	1.00	N
ATOM	1403	CA	LYS	A	96	62.233	50.850	82.234	1.00	1.00	C
ATOM	1404	C	LYS	A	96	62.783	52.003	81.374	1.00	1.00	C
ATOM	1405	O	LYS	A	96	62.000	52.719	80.739	1.00	1.00	O
ATOM	1406	CB	LYS	A	96	62.491	51.118	83.741	1.00	1.00	C
ATOM	1407	CG	LYS	A	96	61.302	50.652	84.602	1.00	1.00	C
ATOM	1408	CD	LYS	A	96	60.251	51.763	84.738	1.00	1.00	C
ATOM	1409	CE	LYS	A	96	58.865	51.220	85.103	1.00	1.00	C
ATOM	1410	NZ	LYS	A	96	57.904	52.311	85.345	1.00	1.00	N1+
ATOM	1411	H	LYS	A	96	63.663	49.296	82.422	1.00	1.00	H
ATOM	1412	HA	LYS	A	96	61.158	50.793	82.062	1.00	1.00	H
ATOM	1413	HB2	LYS	A	96	63.376	50.580	84.072	1.00	1.00	H
ATOM	1414	HB3	LYS	A	96	62.666	52.181	83.916	1.00	1.00	H
ATOM	1415	HG2	LYS	A	96	60.859	49.758	84.158	1.00	1.00	H
ATOM	1416	HG3	LYS	A	96	61.663	50.391	85.598	1.00	1.00	H
ATOM	1417	HD2	LYS	A	96	60.586	52.470	85.499	1.00	1.00	H
ATOM	1418	HD3	LYS	A	96	60.175	52.292	83.792	1.00	1.00	H
ATOM	1419	HE2	LYS	A	96	58.504	50.611	84.272	1.00	1.00	H
ATOM	1420	HE3	LYS	A	96	58.933	50.592	85.993	1.00	1.00	H
ATOM	1421	HZ1	LYS	A	96	58.061	53.063	84.677	1.00	1.00	H
ATOM	1422	HZ2	LYS	A	96	56.952	51.972	85.178	1.00	1.00	H
ATOM	1423	HZ3	LYS	A	96	57.987	52.685	86.277	1.00	1.00	H
ATOM	1424	N	ILE	A	97	64.112	52.145	81.330	1.00	1.00	N
ATOM	1425	CA	ILE	A	97	64.885	52.977	80.418	1.00	1.00	C
ATOM	1426	C	ILE	A	97	65.235	52.045	79.242	1.00	1.00	C
ATOM	1427	O	ILE	A	97	66.312	51.447	79.173	1.00	1.00	O
ATOM	1428	CB	ILE	A	97	66.175	53.493	81.134	1.00	1.00	C
ATOM	1429	CG1	ILE	A	97	65.814	54.259	82.431	1.00	1.00	C
ATOM	1430	CG2	ILE	A	97	66.984	54.405	80.189	1.00	1.00	C
ATOM	1431	CD1	ILE	A	97	67.024	54.779	83.219	1.00	1.00	C
ATOM	1432	H	ILE	A	97	64.666	51.559	81.968	1.00	1.00	H
ATOM	1433	HA	ILE	A	97	64.289	53.819	80.066	1.00	1.00	H
ATOM	1434	HB	ILE	A	97	66.796	52.638	81.407	1.00	1.00	H
ATOM	1435	HG12	ILE	A	97	65.166	55.102	82.186	1.00	1.00	H
ATOM	1436	HG13	ILE	A	97	65.265	53.597	83.101	1.00	1.00	H
ATOM	1437	HG21	ILE	A	97	66.465	55.350	80.042	1.00	1.00	H
ATOM	1438	HG22	ILE	A	97	67.965	54.602	80.618	1.00	1.00	H
ATOM	1439	HG23	ILE	A	97	67.143	53.928	79.223	1.00	1.00	H
ATOM	1440	HD11	ILE	A	97	67.529	55.570	82.666	1.00	1.00	H
ATOM	1441	HD12	ILE	A	97	66.684	55.186	84.171	1.00	1.00	H
ATOM	1442	HD13	ILE	A	97	67.719	53.961	83.412	1.00	1.00	H
ATOM	1443	N	LYS	A	98	64.294	51.909	78.307	1.00	1.00	N
ATOM	1444	CA	LYS	A	98	64.426	51.079	77.118	1.00	1.00	C
ATOM	1445	C	LYS	A	98	65.218	51.893	76.067	1.00	1.00	C
ATOM	1446	O	LYS	A	98	64.782	52.997	75.720	1.00	1.00	O
ATOM	1447	CB	LYS	A	98	62.992	50.805	76.612	1.00	1.00	C
ATOM	1448	CG	LYS	A	98	62.817	50.010	75.308	1.00	1.00	C
ATOM	1449	CD	LYS	A	98	61.307	49.968	75.010	1.00	1.00	C
ATOM	1450	CE	LYS	A	98	60.911	49.085	73.826	1.00	1.00	C

ATOM 1451 NZ LYS A 98 59.442 49.071 73.662 1.00 1.00 N1+
ATOM 1452 H LYS A 98 63.467 52.501 78.370 1.00 1.00 H
ATOM 1453 HA LYS A 98 64.911 50.136 77.367 1.00 1.00 H
ATOM 1454 HB2 LYS A 98 62.469 50.266 77.405 1.00 1.00 H
ATOM 1455 HB3 LYS A 98 62.491 51.759 76.497 1.00 1.00 H
ATOM 1456 HG2 LYS A 98 63.343 50.501 74.487 1.00 1.00 H
ATOM 1457 HG3 LYS A 98 63.205 48.998 75.440 1.00 1.00 H
ATOM 1458 HD2 LYS A 98 60.787 49.601 75.896 1.00 1.00 H
ATOM 1459 HD3 LYS A 98 60.966 50.981 74.802 1.00 1.00 H
ATOM 1460 HE2 LYS A 98 61.379 49.462 72.916 1.00 1.00 H
ATOM 1461 HE3 LYS A 98 61.259 48.067 74.006 1.00 1.00 H
ATOM 1462 HZ1 LYS A 98 59.176 48.397 72.934 1.00 1.00 H
ATOM 1463 HZ2 LYS A 98 58.974 48.744 74.493 1.00 1.00 H
ATOM 1464 HZ3 LYS A 98 59.080 49.970 73.381 1.00 1.00 H
ATOM 1465 N PRO A 99 66.360 51.390 75.545 1.00 1.00 N
ATOM 1466 CA PRO A 99 67.111 52.070 74.481 1.00 1.00 C
ATOM 1467 C PRO A 99 66.360 51.885 73.151 1.00 1.00 C
ATOM 1468 O PRO A 99 66.119 50.749 72.743 1.00 1.00 O
ATOM 1469 CB PRO A 99 68.482 51.374 74.482 1.00 1.00 C
ATOM 1470 CG PRO A 99 68.195 49.960 74.993 1.00 1.00 C
ATOM 1471 CD PRO A 99 67.018 50.154 75.948 1.00 1.00 C
ATOM 1472 HA PRO A 99 67.231 53.133 74.699 1.00 1.00 H
ATOM 1473 HB2 PRO A 99 68.939 51.361 73.492 1.00 1.00 H
ATOM 1474 HB3 PRO A 99 69.138 51.883 75.189 1.00 1.00 H
ATOM 1475 HG2 PRO A 99 67.893 49.319 74.164 1.00 1.00 H
ATOM 1476 HG3 PRO A 99 69.058 49.536 75.505 1.00 1.00 H
ATOM 1477 HD2 PRO A 99 66.339 49.303 75.878 1.00 1.00 H
ATOM 1478 HD3 PRO A 99 67.387 50.258 76.969 1.00 1.00 H
ATOM 1479 N TYR A 100 65.958 52.980 72.503 1.00 1.00 N
ATOM 1480 CA TYR A 100 65.127 52.987 71.302 1.00 1.00 C
ATOM 1481 C TYR A 100 66.078 53.221 70.116 1.00 1.00 C
ATOM 1482 O TYR A 100 66.326 54.356 69.711 1.00 1.00 O
ATOM 1483 CB TYR A 100 64.081 54.119 71.474 1.00 1.00 C
ATOM 1484 CG TYR A 100 62.820 53.828 70.699 1.00 1.00 C
ATOM 1485 CD1 TYR A 100 61.868 52.933 71.230 1.00 1.00 C
ATOM 1486 CD2 TYR A 100 62.618 54.408 69.435 1.00 1.00 C
ATOM 1487 CE1 TYR A 100 60.714 52.610 70.492 1.00 1.00 C
ATOM 1488 CE2 TYR A 100 61.463 54.082 68.707 1.00 1.00 C
ATOM 1489 CZ TYR A 100 60.520 53.182 69.226 1.00 1.00 C
ATOM 1490 OH TYR A 100 59.418 52.872 68.494 1.00 1.00 O
ATOM 1491 H TYR A 100 66.312 53.881 72.824 1.00 1.00 H
ATOM 1492 HA TYR A 100 64.617 52.029 71.190 1.00 1.00 H
ATOM 1493 HB2 TYR A 100 63.802 54.189 72.527 1.00 1.00 H
ATOM 1494 HB3 TYR A 100 64.487 55.090 71.191 1.00 1.00 H
ATOM 1495 HD1 TYR A 100 62.028 52.485 72.201 1.00 1.00 H
ATOM 1496 HD2 TYR A 100 63.350 55.092 69.021 1.00 1.00 H
ATOM 1497 HE1 TYR A 100 59.977 51.927 70.888 1.00 1.00 H
ATOM 1498 HE2 TYR A 100 61.298 54.512 67.735 1.00 1.00 H
ATOM 1499 HH TYR A 100 59.204 53.593 67.840 1.00 1.00 H
ATOM 1500 N VAL A 101 66.700 52.138 69.640 1.00 1.00 N

```
ATOM   1501  CA  VAL A 101      67.670  52.154  68.553  1.00  1.00           C
ATOM   1502  C   VAL A 101      66.882  51.857  67.269  1.00  1.00           C
ATOM   1503  O   VAL A 101      66.183  50.842  67.195  1.00  1.00           O
ATOM   1504  CB  VAL A 101      68.727  51.041  68.809  1.00  1.00           C
ATOM   1505  CG1 VAL A 101      69.720  50.866  67.651  1.00  1.00           C
ATOM   1506  CG2 VAL A 101      69.551  51.357  70.068  1.00  1.00           C
ATOM   1507  H   VAL A 101      66.432  51.236  70.019  1.00  1.00           H
ATOM   1508  HA  VAL A 101      68.164  53.124  68.482  1.00  1.00           H
ATOM   1509  HB  VAL A 101      68.223  50.090  68.963  1.00  1.00           H
ATOM   1510 HG11 VAL A 101      70.437  50.080  67.892  1.00  1.00           H
ATOM   1511 HG12 VAL A 101      69.191  50.577  66.743  1.00  1.00           H
ATOM   1512 HG13 VAL A 101      70.250  51.801  67.469  1.00  1.00           H
ATOM   1513 HG21 VAL A 101      70.272  50.558  70.244  1.00  1.00           H
ATOM   1514 HG22 VAL A 101      70.077  52.304  69.942  1.00  1.00           H
ATOM   1515 HG23 VAL A 101      68.896  51.424  70.936  1.00  1.00           H
ATOM   1516  N   LEU A 102      66.996  52.747  66.279  1.00  1.00           N
ATOM   1517  CA  LEU A 102      66.353  52.642  64.977  1.00  1.00           C
ATOM   1518  C   LEU A 102      67.400  52.700  63.863  1.00  1.00           C
ATOM   1519  O   LEU A 102      68.357  53.473  63.946  1.00  1.00           O
ATOM   1520  CB  LEU A 102      65.378  53.818  64.761  1.00  1.00           C
ATOM   1521  CG  LEU A 102      64.228  53.953  65.772  1.00  1.00           C
ATOM   1522  CD1 LEU A 102      63.442  55.219  65.438  1.00  1.00           C
ATOM   1523  CD2 LEU A 102      63.280  52.759  65.731  1.00  1.00           C
ATOM   1524  H   LEU A 102      67.591  53.564  66.434  1.00  1.00           H
ATOM   1525  HA  LEU A 102      65.809  51.704  64.909  1.00  1.00           H
ATOM   1526  HB2 LEU A 102      65.954  54.739  64.781  1.00  1.00           H
ATOM   1527  HB3 LEU A 102      64.952  53.725  63.762  1.00  1.00           H
ATOM   1528  HG  LEU A 102      64.628  54.056  66.780  1.00  1.00           H
ATOM   1529 HD11 LEU A 102      64.087  56.093  65.543  1.00  1.00           H
ATOM   1530 HD12 LEU A 102      63.046  55.168  64.426  1.00  1.00           H
ATOM   1531 HD13 LEU A 102      62.612  55.315  66.129  1.00  1.00           H
ATOM   1532 HD21 LEU A 102      62.962  52.552  64.710  1.00  1.00           H
ATOM   1533 HD22 LEU A 102      63.810  51.894  66.114  1.00  1.00           H
ATOM   1534 HD23 LEU A 102      62.405  52.944  66.350  1.00  1.00           H
ATOM   1535  N   LEU A 103      67.132  51.989  62.769  1.00  1.00           N
ATOM   1536  CA  LEU A 103      67.670  52.239  61.437  1.00  1.00           C
ATOM   1537  C   LEU A 103      66.415  52.354  60.561  1.00  1.00           C
ATOM   1538  O   LEU A 103      65.510  51.533  60.696  1.00  1.00           O
ATOM   1539  CB  LEU A 103      68.598  51.067  61.029  1.00  1.00           C
ATOM   1540  CG  LEU A 103      69.274  51.065  59.640  1.00  1.00           C
ATOM   1541  CD1 LEU A 103      68.302  50.947  58.461  1.00  1.00           C
ATOM   1542  CD2 LEU A 103      70.161  52.296  59.443  1.00  1.00           C
ATOM   1543  H   LEU A 103      66.357  51.328  62.831  1.00  1.00           H
ATOM   1544  HA  LEU A 103      68.224  53.177  61.418  1.00  1.00           H
ATOM   1545  HB2 LEU A 103      69.394  51.012  61.773  1.00  1.00           H
ATOM   1546  HB3 LEU A 103      68.028  50.140  61.116  1.00  1.00           H
ATOM   1547  HG  LEU A 103      69.920  50.187  59.606  1.00  1.00           H
ATOM   1548 HD11 LEU A 103      68.854  50.628  57.578  1.00  1.00           H
ATOM   1549 HD12 LEU A 103      67.537  50.200  58.681  1.00  1.00           H
ATOM   1550 HD13 LEU A 103      67.838  51.905  58.240  1.00  1.00           H
```

ATOM 1551 HD21 LEU A 103 70.667 52.230 58.480 1.00 1.00 H
ATOM 1552 HD22 LEU A 103 69.558 53.201 59.466 1.00 1.00 H
ATOM 1553 HD23 LEU A 103 70.910 52.342 60.234 1.00 1.00 H
ATOM 1554 N GLY A 104 66.292 53.402 59.744 1.00 1.00 N
ATOM 1555 CA GLY A 104 65.133 53.621 58.891 1.00 1.00 C
ATOM 1556 C GLY A 104 65.477 54.065 57.476 1.00 1.00 C
ATOM 1557 O GLY A 104 66.484 54.734 57.243 1.00 1.00 O
ATOM 1558 H GLY A 104 67.052 54.081 59.698 1.00 1.00 H
ATOM 1559 HA2 GLY A 104 64.577 52.688 58.803 1.00 1.00 H
ATOM 1560 HA3 GLY A 104 64.479 54.366 59.343 1.00 1.00 H
ATOM 1561 N ALA A 105 64.583 53.734 56.542 1.00 1.00 N
ATOM 1562 CA ALA A 105 64.555 54.223 55.172 1.00 1.00 C
ATOM 1563 C ALA A 105 63.564 55.392 55.138 1.00 1.00 C
ATOM 1564 O ALA A 105 62.452 55.276 55.662 1.00 1.00 O
ATOM 1565 CB ALA A 105 64.003 53.107 54.270 1.00 1.00 C
ATOM 1566 H ALA A 105 63.743 53.259 56.879 1.00 1.00 H
ATOM 1567 HA ALA A 105 65.546 54.532 54.836 1.00 1.00 H
ATOM 1568 HB1 ALA A 105 63.930 53.467 53.243 1.00 1.00 H
ATOM 1569 HB2 ALA A 105 64.677 52.250 54.298 1.00 1.00 H
ATOM 1570 HB3 ALA A 105 63.015 52.794 54.611 1.00 1.00 H
ATOM 1571 N GLY A 106 63.970 56.513 54.539 1.00 1.00 N
ATOM 1572 CA GLY A 106 63.220 57.758 54.508 1.00 1.00 C
ATOM 1573 C GLY A 106 62.842 58.140 53.080 1.00 1.00 C
ATOM 1574 O GLY A 106 63.650 57.974 52.168 1.00 1.00 O
ATOM 1575 H GLY A 106 64.893 56.524 54.098 1.00 1.00 H
ATOM 1576 HA2 GLY A 106 62.316 57.677 55.106 1.00 1.00 H
ATOM 1577 HA3 GLY A 106 63.836 58.549 54.927 1.00 1.00 H
ATOM 1578 N HIE A 107 61.632 58.672 52.899 1.00 1.00 N
ATOM 1579 CA HIE A 107 61.061 59.196 51.662 1.00 1.00 C
ATOM 1580 C HIE A 107 60.652 60.643 51.966 1.00 1.00 C
ATOM 1581 O HIE A 107 59.786 60.868 52.812 1.00 1.00 O
ATOM 1582 CB HIE A 107 59.861 58.310 51.267 1.00 1.00 C
ATOM 1583 CG HIE A 107 59.084 58.809 50.078 1.00 1.00 C
ATOM 1584 CD2 HIE A 107 59.600 59.329 48.924 1.00 1.00 C
ATOM 1585 ND1 HIE A 107 57.693 58.800 49.991 1.00 1.00 N
ATOM 1586 CE1 HIE A 107 57.400 59.330 48.795 1.00 1.00 C
ATOM 1587 NE2 HIE A 107 58.521 59.652 48.135 1.00 1.00 N
ATOM 1588 H HIE A 107 61.030 58.756 53.722 1.00 1.00 H
ATOM 1589 HA HIE A 107 61.805 59.174 50.866 1.00 1.00 H
ATOM 1590 HB2 HIE A 107 60.225 57.308 51.040 1.00 1.00 H
ATOM 1591 HB3 HIE A 107 59.177 58.236 52.111 1.00 1.00 H
ATOM 1592 HD2 HIE A 107 60.644 59.466 48.684 1.00 1.00 H
ATOM 1593 HE1 HIE A 107 56.400 59.475 48.412 1.00 1.00 H
ATOM 1594 HE2 HIE A 107 58.535 60.076 47.201 1.00 1.00 H
ATOM 1595 N TYR A 108 61.321 61.605 51.330 1.00 1.00 N
ATOM 1596 CA TYR A 108 61.191 63.037 51.570 1.00 1.00 C
ATOM 1597 C TYR A 108 60.782 63.707 50.257 1.00 1.00 C
ATOM 1598 O TYR A 108 61.464 63.532 49.248 1.00 1.00 O
ATOM 1599 CB TYR A 108 62.544 63.603 52.059 1.00 1.00 C
ATOM 1600 CG TYR A 108 62.872 63.256 53.509 1.00 1.00 C

ATOM	1601	CD1	TYR	A	108	63.139	61.924	53.888	1.00	1.00	C
ATOM	1602	CD2	TYR	A	108	62.900	64.264	54.495	1.00	1.00	C
ATOM	1603	CE1	TYR	A	108	63.377	61.599	55.234	1.00	1.00	C
ATOM	1604	CE2	TYR	A	108	63.162	63.939	55.841	1.00	1.00	C
ATOM	1605	CZ	TYR	A	108	63.389	62.603	56.211	1.00	1.00	C
ATOM	1606	OH	TYR	A	108	63.637	62.270	57.509	1.00	1.00	O
ATOM	1607	H	TYR	A	108	61.945	61.325	50.570	1.00	1.00	H
ATOM	1608	HA	TYR	A	108	60.425	63.226	52.320	1.00	1.00	H
ATOM	1609	HB2	TYR	A	108	63.347	63.239	51.415	1.00	1.00	H
ATOM	1610	HB3	TYR	A	108	62.517	64.689	51.961	1.00	1.00	H
ATOM	1611	HD1	TYR	A	108	63.157	61.143	53.144	1.00	1.00	H
ATOM	1612	HD2	TYR	A	108	62.718	65.294	54.223	1.00	1.00	H
ATOM	1613	HE1	TYR	A	108	63.560	60.579	55.526	1.00	1.00	H
ATOM	1614	HE2	TYR	A	108	63.184	64.724	56.584	1.00	1.00	H
ATOM	1615	HH	TYR	A	108	63.481	63.003	58.109	1.00	1.00	H
ATOM	1616	N	LYS	A	109	59.692	64.477	50.262	1.00	1.00	N
ATOM	1617	CA	LYS	A	109	59.126	65.119	49.083	1.00	1.00	C
ATOM	1618	C	LYS	A	109	58.651	66.537	49.434	1.00	1.00	C
ATOM	1619	O	LYS	A	109	58.141	66.757	50.534	1.00	1.00	O
ATOM	1620	CB	LYS	A	109	58.060	64.179	48.490	1.00	1.00	C
ATOM	1621	CG	LYS	A	109	57.284	64.789	47.315	1.00	1.00	C
ATOM	1622	CD	LYS	A	109	56.882	63.700	46.316	1.00	1.00	C
ATOM	1623	CE	LYS	A	109	55.822	64.251	45.371	1.00	1.00	C
ATOM	1624	NZ	LYS	A	109	55.818	63.577	44.065	1.00	1.00	N1+
ATOM	1625	H	LYS	A	109	59.124	64.520	51.108	1.00	1.00	H
ATOM	1626	HA	LYS	A	109	59.910	65.225	48.331	1.00	1.00	H
ATOM	1627	HB2	LYS	A	109	58.572	63.275	48.155	1.00	1.00	H
ATOM	1628	HB3	LYS	A	109	57.347	63.894	49.266	1.00	1.00	H
ATOM	1629	HG2	LYS	A	109	56.397	65.297	47.700	1.00	1.00	H
ATOM	1630	HG3	LYS	A	109	57.901	65.515	46.785	1.00	1.00	H
ATOM	1631	HD2	LYS	A	109	57.771	63.412	45.753	1.00	1.00	H
ATOM	1632	HD3	LYS	A	109	56.482	62.827	46.834	1.00	1.00	H
ATOM	1633	HE2	LYS	A	109	54.844	64.165	45.845	1.00	1.00	H
ATOM	1634	HE3	LYS	A	109	56.034	65.307	45.202	1.00	1.00	H
ATOM	1635	HZ1	LYS	A	109	56.606	63.927	43.525	1.00	1.00	H
ATOM	1636	HZ2	LYS	A	109	55.926	62.561	44.152	1.00	1.00	H
ATOM	1637	HZ3	LYS	A	109	54.987	63.831	43.555	1.00	1.00	H
ATOM	1638	N	TYR	A	110	58.838	67.463	48.526	1.00	0.00	N
ATOM	1639	CA	TYR	A	110	58.333	68.833	48.502	1.00	0.00	C
ATOM	1640	C	TYR	A	110	57.446	69.061	47.302	1.00	0.00	C
ATOM	1641	O	TYR	A	110	57.897	68.664	46.206	1.00	0.00	O
ATOM	1642	CB	TYR	A	110	59.505	69.856	48.434	1.00	0.00	C
ATOM	1643	CG	TYR	A	110	60.437	69.917	49.650	1.00	0.00	C
ATOM	1644	CD1	TYR	A	110	61.586	69.122	49.708	1.00	0.00	C
ATOM	1645	CD2	TYR	A	110	60.142	70.781	50.709	1.00	0.00	C
ATOM	1646	CE1	TYR	A	110	62.432	69.198	50.811	1.00	0.00	C
ATOM	1647	CE2	TYR	A	110	60.989	70.855	51.810	1.00	0.00	C
ATOM	1648	CZ	TYR	A	110	62.134	70.063	51.861	1.00	0.00	C
ATOM	1649	OH	TYR	A	110	62.968	70.135	52.939	1.00	0.00	O
ATOM	1650	H	TYR	A	110	59.417	67.189	47.725	1.00	0.00	H

ATOM	1651	HA	TYR	A	110	57.727	69.008	49.411	1.00	0.00	H
ATOM	1652	2HB	TYR	A	110	59.100	70.875	48.273	1.00	0.00	H
ATOM	1653	3HB	TYR	A	110	60.103	69.669	47.518	1.00	0.00	H
ATOM	1654	1HD	TYR	A	110	61.829	68.452	48.895	1.00	0.00	H
ATOM	1655	2HD	TYR	A	110	59.261	71.407	50.671	1.00	0.00	H
ATOM	1656	1HE	TYR	A	110	63.324	68.590	50.854	1.00	0.00	H
ATOM	1657	2HE	TYR	A	110	60.753	71.531	52.618	1.00	0.00	H
ATOM	1658	HH	TYR	A	110	62.611	70.777	53.557	1.00	0.00	H
ATOM	1659	N	ASP	A	111	56.260	69.635	47.437	1.00	1.00	N
ATOM	1660	CA	ASP	A	111	55.327	69.949	46.348	1.00	1.00	C
ATOM	1661	C	ASP	A	111	54.980	71.437	46.368	1.00	1.00	C
ATOM	1662	O	ASP	A	111	54.617	71.982	47.415	1.00	1.00	O
ATOM	1663	CB	ASP	A	111	53.998	69.157	46.388	1.00	1.00	C
ATOM	1664	CG	ASP	A	111	54.119	67.653	46.651	1.00	1.00	C
ATOM	1665	OD1	ASP	A	111	54.572	66.925	45.731	1.00	1.00	O
ATOM	1666	OD2	ASP	A	111	53.724	67.253	47.773	1.00	1.00	O1-
ATOM	1667	H	ASP	A	111	55.989	69.922	48.379	1.00	1.00	H
ATOM	1668	HA	ASP	A	111	55.811	69.711	45.403	1.00	1.00	H
ATOM	1669	HB2	ASP	A	111	53.337	69.594	47.131	1.00	1.00	H
ATOM	1670	HB3	ASP	A	111	53.502	69.294	45.425	1.00	1.00	H
ATOM	1671	N	PHE	A	112	55.023	72.076	45.197	1.00	1.00	N
ATOM	1672	CA	PHE	A	112	54.481	73.401	44.892	1.00	1.00	C
ATOM	1673	C	PHE	A	112	54.527	73.623	43.373	1.00	1.00	C
ATOM	1674	O	PHE	A	112	55.255	72.919	42.673	1.00	1.00	O
ATOM	1675	CB	PHE	A	112	55.199	74.527	45.675	1.00	1.00	C
ATOM	1676	CG	PHE	A	112	56.656	74.732	45.311	1.00	1.00	C
ATOM	1677	CD1	PHE	A	112	57.663	74.052	46.021	1.00	1.00	C
ATOM	1678	CD2	PHE	A	112	57.005	75.606	44.262	1.00	1.00	C
ATOM	1679	CE1	PHE	A	112	59.013	74.243	45.677	1.00	1.00	C
ATOM	1680	CE2	PHE	A	112	58.354	75.783	43.912	1.00	1.00	C
ATOM	1681	CZ	PHE	A	112	59.359	75.102	44.620	1.00	1.00	C
ATOM	1682	H	PHE	A	112	55.393	71.556	44.403	1.00	1.00	H
ATOM	1683	HA	PHE	A	112	53.433	73.408	45.197	1.00	1.00	H
ATOM	1684	HB2	PHE	A	112	54.666	75.458	45.481	1.00	1.00	H
ATOM	1685	HB3	PHE	A	112	55.120	74.342	46.744	1.00	1.00	H
ATOM	1686	HD1	PHE	A	112	57.401	73.381	46.829	1.00	1.00	H
ATOM	1687	HD2	PHE	A	112	56.238	76.142	43.718	1.00	1.00	H
ATOM	1688	HE1	PHE	A	112	59.785	73.719	46.221	1.00	1.00	H
ATOM	1689	HE2	PHE	A	112	58.616	76.446	43.100	1.00	1.00	H
ATOM	1690	HZ	PHE	A	112	60.397	75.241	44.356	1.00	1.00	H
ATOM	1691	N	ASP	A	113	53.793	74.621	42.864	1.00	1.00	N
ATOM	1692	CA	ASP	A	113	53.813	75.064	41.466	1.00	1.00	C
ATOM	1693	C	ASP	A	113	53.637	76.592	41.400	1.00	1.00	C
ATOM	1694	O	ASP	A	113	53.393	77.234	42.425	1.00	1.00	O
ATOM	1695	CB	ASP	A	113	52.876	74.204	40.572	1.00	1.00	C
ATOM	1696	CG	ASP	A	113	51.402	74.626	40.704	1.00	1.00	C
ATOM	1697	OD1	ASP	A	113	50.733	74.320	41.719	1.00	1.00	O
ATOM	1698	OD2	ASP	A	113	50.903	75.306	39.778	1.00	1.00	O1-
ATOM	1699	H	ASP	A	113	53.190	75.137	43.492	1.00	1.00	H
ATOM	1700	HA	ASP	A	113	54.815	74.888	41.090	1.00	1.00	H

```
ATOM   1701  HB2 ASP A 113      53.187  74.326  39.533  1.00  1.00           H
ATOM   1702  HB3 ASP A 113      52.983  73.149  40.828  1.00  1.00           H
ATOM   1703  N   GLY A 114      53.759  77.169  40.198  1.00  1.00           N
ATOM   1704  CA  GLY A 114      53.624  78.593  39.926  1.00  1.00           C
ATOM   1705  C   GLY A 114      54.808  79.118  39.111  1.00  1.00           C
ATOM   1706  O   GLY A 114      55.134  78.569  38.059  1.00  1.00           O
ATOM   1707  H   GLY A 114      54.019  76.571  39.421  1.00  1.00           H
ATOM   1708  HA2 GLY A 114      52.709  78.767  39.362  1.00  1.00           H
ATOM   1709  HA3 GLY A 114      53.559  79.148  40.862  1.00  1.00           H
ATOM   1710  N   VAL A 115      55.419  80.221  39.556  1.00  1.00           N
ATOM   1711  CA  VAL A 115      56.531  80.898  38.893  1.00  1.00           C
ATOM   1712  C   VAL A 115      57.424  81.571  39.949  1.00  1.00           C
ATOM   1713  O   VAL A 115      57.585  82.791  39.988  1.00  1.00           O
ATOM   1714  CB  VAL A 115      56.048  81.743  37.678  1.00  1.00           C
ATOM   1715  CG1 VAL A 115      55.115  82.902  38.056  1.00  1.00           C
ATOM   1716  CG2 VAL A 115      57.228  82.268  36.849  1.00  1.00           C
ATOM   1717  H   VAL A 115      55.111  80.594  40.443  1.00  1.00           H
ATOM   1718  HA  VAL A 115      57.152  80.110  38.464  1.00  1.00           H
ATOM   1719  HB  VAL A 115      55.481  81.079  37.024  1.00  1.00           H
ATOM   1720 HG11 VAL A 115      54.263  82.529  38.624  1.00  1.00           H
ATOM   1721 HG12 VAL A 115      55.642  83.650  38.647  1.00  1.00           H
ATOM   1722 HG13 VAL A 115      54.742  83.376  37.148  1.00  1.00           H
ATOM   1723 HG21 VAL A 115      56.851  82.743  35.943  1.00  1.00           H
ATOM   1724 HG22 VAL A 115      57.798  83.002  37.417  1.00  1.00           H
ATOM   1725 HG23 VAL A 115      57.879  81.440  36.570  1.00  1.00           H
ATOM   1726  N   ASN A 116      58.006  80.751  40.827  1.00  1.00           N
ATOM   1727  CA  ASN A 116      58.908  81.062  41.942  1.00  1.00           C
ATOM   1728  C   ASN A 116      60.282  81.571  41.433  1.00  1.00           C
ATOM   1729  O   ASN A 116      61.330  81.001  41.733  1.00  1.00           O
ATOM   1730  CB  ASN A 116      59.098  79.794  42.829  1.00  1.00           C
ATOM   1731  CG  ASN A 116      57.810  79.185  43.372  1.00  1.00           C
ATOM   1732  ND2 ASN A 116      57.634  79.153  44.685  1.00  1.00           N
ATOM   1733  OD1 ASN A 116      56.986  78.690  42.616  1.00  1.00           O
ATOM   1734  H   ASN A 116      57.835  79.764  40.675  1.00  1.00           H
ATOM   1735  HA  ASN A 116      58.454  81.849  42.545  1.00  1.00           H
ATOM   1736  HB2 ASN A 116      59.591  79.022  42.241  1.00  1.00           H
ATOM   1737  HB3 ASN A 116      59.755  80.043  43.662  1.00  1.00           H
ATOM   1738 HD21 ASN A 116      58.323  79.555  45.300  1.00  1.00           H
ATOM   1739 HD22 ASN A 116      56.796  78.706  45.027  1.00  1.00           H
ATOM   1740  N   ARG A 117      60.269  82.625  40.604  1.00  1.00           N
ATOM   1741  CA  ARG A 117      61.401  83.413  40.104  1.00  1.00           C
ATOM   1742  C   ARG A 117      62.285  82.680  39.075  1.00  1.00           C
ATOM   1743  O   ARG A 117      63.448  83.032  38.895  1.00  1.00           O
ATOM   1744  CB  ARG A 117      62.185  84.122  41.235  1.00  1.00           C
ATOM   1745  CG  ARG A 117      61.395  85.316  41.797  1.00  1.00           C
ATOM   1746  CD  ARG A 117      62.108  86.065  42.934  1.00  1.00           C
ATOM   1747  NE  ARG A 117      63.409  86.649  42.537  1.00  1.00           N
ATOM   1748  CZ  ARG A 117      64.618  86.184  42.857  1.00  1.00           C
ATOM   1749  NH1 ARG A 117      64.778  85.024  43.446  1.00  1.00           N1+
ATOM   1750  NH2 ARG A 117      65.709  86.877  42.603  1.00  1.00           N1+
```

```
ATOM   1751  H   ARG A 117      59.339  82.969  40.362  1.00  1.00           H
ATOM   1752  HA  ARG A 117      60.948  84.212  39.516  1.00  1.00           H
ATOM   1753  HB2 ARG A 117      62.417  83.415  42.031  1.00  1.00           H
ATOM   1754  HB3 ARG A 117      63.124  84.507  40.840  1.00  1.00           H
ATOM   1755  HG2 ARG A 117      61.197  86.023  40.990  1.00  1.00           H
ATOM   1756  HG3 ARG A 117      60.436  84.960  42.176  1.00  1.00           H
ATOM   1757  HD2 ARG A 117      61.456  86.878  43.258  1.00  1.00           H
ATOM   1758  HD3 ARG A 117      62.225  85.399  43.786  1.00  1.00           H
ATOM   1759  HE  ARG A 117      63.372  87.528  42.051  1.00  1.00           H
ATOM   1760 HH11 ARG A 117      63.971  84.455  43.707  1.00  1.00           H
ATOM   1761 HH12 ARG A 117      65.689  84.657  43.720  1.00  1.00           H
ATOM   1762 HH21 ARG A 117      65.657  87.777  42.160  1.00  1.00           H
ATOM   1763 HH22 ARG A 117      66.597  86.504  42.893  1.00  1.00           H
ATOM   1764  N   GLY A 118      61.727  81.692  38.368  1.00  1.00           N
ATOM   1765  CA  GLY A 118      62.413  80.914  37.336  1.00  1.00           C
ATOM   1766  C   GLY A 118      62.236  79.417  37.568  1.00  1.00           C
ATOM   1767  O   GLY A 118      62.085  78.666  36.607  1.00  1.00           O
ATOM   1768  H   GLY A 118      60.767  81.457  38.579  1.00  1.00           H
ATOM   1769  HA2 GLY A 118      62.005  81.168  36.358  1.00  1.00           H
ATOM   1770  HA3 GLY A 118      63.481  81.132  37.339  1.00  1.00           H
ATOM   1771  N   THR A 119      62.126  78.997  38.836  1.00  1.00           N
ATOM   1772  CA  THR A 119      61.536  77.712  39.207  1.00  1.00           C
ATOM   1773  C   THR A 119      60.017  77.841  38.977  1.00  1.00           C
ATOM   1774  O   THR A 119      59.447  78.904  39.223  1.00  1.00           O
ATOM   1775  CB  THR A 119      61.865  77.437  40.696  1.00  1.00           C
ATOM   1776  CG2 THR A 119      61.328  76.092  41.188  1.00  1.00           C
ATOM   1777  OG1 THR A 119      63.251  77.461  40.919  1.00  1.00           O
ATOM   1778  H   THR A 119      62.319  79.650  39.584  1.00  1.00           H
ATOM   1779  HA  THR A 119      61.948  76.915  38.586  1.00  1.00           H
ATOM   1780  HB  THR A 119      61.428  78.222  41.306  1.00  1.00           H
ATOM   1781  HG1 THR A 119      63.388  77.416  41.868  1.00  1.00           H
ATOM   1782 HG21 THR A 119      61.687  75.898  42.198  1.00  1.00           H
ATOM   1783 HG22 THR A 119      60.240  76.113  41.212  1.00  1.00           H
ATOM   1784 HG23 THR A 119      61.664  75.290  40.529  1.00  1.00           H
ATOM   1785  N   ARG A 120      59.374  76.793  38.460  1.00  1.00           N
ATOM   1786  CA  ARG A 120      57.952  76.788  38.102  1.00  1.00           C
ATOM   1787  C   ARG A 120      57.185  75.717  38.892  1.00  1.00           C
ATOM   1788  O   ARG A 120      56.037  75.419  38.579  1.00  1.00           O
ATOM   1789  CB  ARG A 120      57.796  76.667  36.569  1.00  1.00           C
ATOM   1790  CG  ARG A 120      58.321  77.918  35.847  1.00  1.00           C
ATOM   1791  CD  ARG A 120      58.116  77.781  34.337  1.00  1.00           C
ATOM   1792  NE  ARG A 120      58.609  78.972  33.620  1.00  1.00           N
ATOM   1793  CZ  ARG A 120      59.374  78.994  32.533  1.00  1.00           C
ATOM   1794  NH1 ARG A 120      59.866  77.901  31.991  1.00  1.00           N1+
ATOM   1795  NH2 ARG A 120      59.657  80.138  31.949  1.00  1.00           N1+
ATOM   1796  H   ARG A 120      59.917  75.973  38.224  1.00  1.00           H
ATOM   1797  HA  ARG A 120      57.510  77.738  38.398  1.00  1.00           H
ATOM   1798  HB2 ARG A 120      58.329  75.782  36.217  1.00  1.00           H
ATOM   1799  HB3 ARG A 120      56.737  76.554  36.329  1.00  1.00           H
ATOM   1800  HG2 ARG A 120      57.781  78.795  36.208  1.00  1.00           H
```

ATOM 1801 HG3 ARG A 120 59.385 78.045 36.050 1.00 1.00 H
ATOM 1802 HD2 ARG A 120 58.631 76.881 34.001 1.00 1.00 H
ATOM 1803 HD3 ARG A 120 57.050 77.662 34.134 1.00 1.00 H
ATOM 1804 HE ARG A 120 58.329 79.860 33.999 1.00 1.00 H
ATOM 1805 HH11 ARG A 120 59.688 77.012 32.424 1.00 1.00 H
ATOM 1806 HH12 ARG A 120 60.411 77.948 31.149 1.00 1.00 H
ATOM 1807 HH21 ARG A 120 59.290 81.001 32.310 1.00 1.00 H
ATOM 1808 HH22 ARG A 120 60.230 80.151 31.124 1.00 1.00 H
ATOM 1809 N GLY A 121 57.818 75.174 39.934 1.00 1.00 N
ATOM 1810 CA GLY A 121 57.310 74.109 40.779 1.00 1.00 C
ATOM 1811 C GLY A 121 58.314 72.974 40.894 1.00 1.00 C
ATOM 1812 O GLY A 121 59.343 72.975 40.216 1.00 1.00 O
ATOM 1813 H GLY A 121 58.762 75.477 40.107 1.00 1.00 H
ATOM 1814 HA2 GLY A 121 57.097 74.502 41.771 1.00 1.00 H
ATOM 1815 HA3 GLY A 121 56.394 73.693 40.359 1.00 1.00 H
ATOM 1816 N THR A 122 57.989 72.013 41.754 1.00 1.00 N
ATOM 1817 CA THR A 122 58.601 70.703 41.876 1.00 1.00 C
ATOM 1818 C THR A 122 57.576 69.790 42.576 1.00 1.00 C
ATOM 1819 O THR A 122 56.629 70.276 43.198 1.00 1.00 O
ATOM 1820 CB THR A 122 60.015 70.778 42.523 1.00 1.00 C
ATOM 1821 CG2 THR A 122 59.969 71.057 44.026 1.00 1.00 C
ATOM 1822 OG1 THR A 122 60.673 69.550 42.315 1.00 1.00 O
ATOM 1823 H THR A 122 57.112 72.142 42.260 1.00 1.00 H
ATOM 1824 HA THR A 122 58.735 70.321 40.863 1.00 1.00 H
ATOM 1825 HB THR A 122 60.607 71.554 42.044 1.00 1.00 H
ATOM 1826 HG1 THR A 122 61.535 69.621 42.733 1.00 1.00 H
ATOM 1827 HG21 THR A 122 60.981 71.182 44.408 1.00 1.00 H
ATOM 1828 HG22 THR A 122 59.408 71.973 44.206 1.00 1.00 H
ATOM 1829 HG23 THR A 122 59.485 70.234 44.553 1.00 1.00 H
ATOM 1830 N SER A 123 57.767 68.481 42.443 1.00 1.00 N
ATOM 1831 CA SER A 123 57.050 67.405 43.116 1.00 1.00 C
ATOM 1832 C SER A 123 57.973 66.174 43.015 1.00 1.00 C
ATOM 1833 O SER A 123 57.541 65.094 42.610 1.00 1.00 O
ATOM 1834 CB SER A 123 55.608 67.261 42.552 1.00 1.00 C
ATOM 1835 OG SER A 123 54.848 66.334 43.316 1.00 1.00 O
ATOM 1836 H SER A 123 58.592 68.206 41.925 1.00 1.00 H
ATOM 1837 HA SER A 123 56.963 67.666 44.169 1.00 1.00 H
ATOM 1838 HB2 SER A 123 55.106 68.228 42.585 1.00 1.00 H
ATOM 1839 HB3 SER A 123 55.655 66.925 41.516 1.00 1.00 H
ATOM 1840 HG SER A 123 54.738 66.659 44.260 1.00 1.00 H
ATOM 1841 N GLU A 124 59.275 66.357 43.284 1.00 1.00 N
ATOM 1842 CA GLU A 124 60.319 65.356 43.063 1.00 1.00 C
ATOM 1843 C GLU A 124 60.389 64.382 44.254 1.00 1.00 C
ATOM 1844 O GLU A 124 60.117 64.775 45.393 1.00 1.00 O
ATOM 1845 CB GLU A 124 61.664 66.107 42.895 1.00 1.00 C
ATOM 1846 CG GLU A 124 62.918 65.234 42.716 1.00 1.00 C
ATOM 1847 CD GLU A 124 62.857 64.312 41.501 1.00 1.00 C
ATOM 1848 OE1 GLU A 124 62.071 63.340 41.572 1.00 1.00 O
ATOM 1849 OE2 GLU A 124 63.606 64.578 40.534 1.00 1.00 O1-
ATOM 1850 H GLU A 124 59.557 67.254 43.653 1.00 1.00 H

ATOM 1851 HA GLU A 124 60.104 64.795 42.153 1.00 1.00 H
ATOM 1852 HB2 GLU A 124 61.582 66.762 42.028 1.00 1.00 H
ATOM 1853 HB3 GLU A 124 61.821 66.741 43.769 1.00 1.00 H
ATOM 1854 HG2 GLU A 124 63.780 65.893 42.609 1.00 1.00 H
ATOM 1855 HG3 GLU A 124 63.077 64.632 43.610 1.00 1.00 H
ATOM 1856 N GLU A 125 60.743 63.123 43.980 1.00 1.00 N
ATOM 1857 CA GLU A 125 60.793 62.035 44.950 1.00 1.00 C
ATOM 1858 C GLU A 125 62.236 61.911 45.481 1.00 1.00 C
ATOM 1859 O GLU A 125 63.148 61.505 44.760 1.00 1.00 O
ATOM 1860 CB GLU A 125 60.409 60.712 44.238 1.00 1.00 C
ATOM 1861 CG GLU A 125 59.020 60.687 43.573 1.00 1.00 C
ATOM 1862 CD GLU A 125 57.843 60.853 44.528 1.00 1.00 C
ATOM 1863 OE1 GLU A 125 58.047 60.792 45.757 1.00 1.00 O
ATOM 1864 OE2 GLU A 125 56.718 61.059 44.018 1.00 1.00 O1-
ATOM 1865 H GLU A 125 61.090 62.950 43.029 1.00 1.00 H
ATOM 1866 HA GLU A 125 60.108 62.222 45.777 1.00 1.00 H
ATOM 1867 HB2 GLU A 125 61.138 60.504 43.454 1.00 1.00 H
ATOM 1868 HB3 GLU A 125 60.471 59.895 44.959 1.00 1.00 H
ATOM 1869 HG2 GLU A 125 58.968 61.468 42.815 1.00 1.00 H
ATOM 1870 HG3 GLU A 125 58.903 59.729 43.067 1.00 1.00 H
ATOM 1871 N GLY A 126 62.457 62.220 46.763 1.00 1.00 N
ATOM 1872 CA GLY A 126 63.761 62.149 47.414 1.00 1.00 C
ATOM 1873 C GLY A 126 63.767 61.028 48.453 1.00 1.00 C
ATOM 1874 O GLY A 126 62.752 60.777 49.105 1.00 1.00 O
ATOM 1875 H GLY A 126 61.689 62.571 47.327 1.00 1.00 H
ATOM 1876 HA2 GLY A 126 64.550 61.972 46.683 1.00 1.00 H
ATOM 1877 HA3 GLY A 126 63.967 63.092 47.920 1.00 1.00 H
ATOM 1878 N THR A 127 64.901 60.342 48.633 1.00 1.00 N
ATOM 1879 CA THR A 127 65.038 59.224 49.558 1.00 1.00 C
ATOM 1880 C THR A 127 66.447 59.183 50.170 1.00 1.00 C
ATOM 1881 O THR A 127 67.416 59.605 49.536 1.00 1.00 O
ATOM 1882 CB THR A 127 64.485 57.918 48.918 1.00 1.00 C
ATOM 1883 CG2 THR A 127 65.265 57.468 47.680 1.00 1.00 C
ATOM 1884 OG1 THR A 127 64.527 56.881 49.869 1.00 1.00 O
ATOM 1885 H THR A 127 65.721 60.589 48.093 1.00 1.00 H
ATOM 1886 HA THR A 127 64.376 59.451 50.391 1.00 1.00 H
ATOM 1887 HB THR A 127 63.443 58.069 48.642 1.00 1.00 H
ATOM 1888 HG1 THR A 127 64.080 57.205 50.676 1.00 1.00 H
ATOM 1889 HG21 THR A 127 64.856 56.524 47.320 1.00 1.00 H
ATOM 1890 HG22 THR A 127 65.172 58.214 46.891 1.00 1.00 H
ATOM 1891 HG23 THR A 127 66.317 57.331 47.930 1.00 1.00 H
ATOM 1892 N LEU A 128 66.543 58.716 51.421 1.00 1.00 N
ATOM 1893 CA LEU A 128 67.755 58.622 52.225 1.00 1.00 C
ATOM 1894 C LEU A 128 67.601 57.523 53.292 1.00 1.00 C
ATOM 1895 O LEU A 128 66.525 56.939 53.441 1.00 1.00 O
ATOM 1896 CB LEU A 128 68.180 60.018 52.754 1.00 1.00 C
ATOM 1897 CG LEU A 128 67.081 60.810 53.501 1.00 1.00 C
ATOM 1898 CD1 LEU A 128 66.657 60.146 54.814 1.00 1.00 C
ATOM 1899 CD2 LEU A 128 67.574 62.221 53.819 1.00 1.00 C
ATOM 1900 H LEU A 128 65.699 58.336 51.842 1.00 1.00 H

ATOM 1901 HA LEU A 128 68.557 58.286 51.566 1.00 1.00 H
ATOM 1902 HB2 LEU A 128 69.051 59.912 53.402 1.00 1.00 H
ATOM 1903 HB3 LEU A 128 68.492 60.611 51.894 1.00 1.00 H
ATOM 1904 HG LEU A 128 66.201 60.910 52.866 1.00 1.00 H
ATOM 1905 HD11 LEU A 128 66.054 60.836 55.401 1.00 1.00 H
ATOM 1906 HD12 LEU A 128 66.059 59.264 54.607 1.00 1.00 H
ATOM 1907 HD13 LEU A 128 67.540 59.874 55.394 1.00 1.00 H
ATOM 1908 HD21 LEU A 128 68.429 62.172 54.494 1.00 1.00 H
ATOM 1909 HD22 LEU A 128 67.863 62.726 52.898 1.00 1.00 H
ATOM 1910 HD23 LEU A 128 66.773 62.792 54.290 1.00 1.00 H
ATOM 1911 N GLY A 129 68.667 57.263 54.054 1.00 1.00 N
ATOM 1912 CA GLY A 129 68.687 56.331 55.176 1.00 1.00 C
ATOM 1913 C GLY A 129 68.986 57.117 56.450 1.00 1.00 C
ATOM 1914 O GLY A 129 69.956 57.876 56.480 1.00 1.00 O
ATOM 1915 H GLY A 129 69.510 57.795 53.881 1.00 1.00 H
ATOM 1916 HA2 GLY A 129 67.731 55.816 55.272 1.00 1.00 H
ATOM 1917 HA3 GLY A 129 69.469 55.587 55.029 1.00 1.00 H
ATOM 1918 N ASN A 130 68.151 56.947 57.477 1.00 1.00 N
ATOM 1919 CA ASN A 130 68.286 57.579 58.791 1.00 1.00 C
ATOM 1920 C ASN A 130 68.658 56.519 59.830 1.00 1.00 C
ATOM 1921 O ASN A 130 68.202 55.379 59.755 1.00 1.00 O
ATOM 1922 CB ASN A 130 66.947 58.217 59.253 1.00 1.00 C
ATOM 1923 CG ASN A 130 66.546 59.483 58.479 1.00 1.00 C
ATOM 1924 ND2 ASN A 130 65.255 59.780 58.360 1.00 1.00 N
ATOM 1925 OD1 ASN A 130 67.386 60.229 58.001 1.00 1.00 O
ATOM 1926 H ASN A 130 67.450 56.211 57.387 1.00 1.00 H
ATOM 1927 HA ASN A 130 69.061 58.346 58.765 1.00 1.00 H
ATOM 1928 HB2 ASN A 130 66.150 57.476 59.175 1.00 1.00 H
ATOM 1929 HB3 ASN A 130 67.034 58.505 60.299 1.00 1.00 H
ATOM 1930 HD21 ASN A 130 64.542 59.184 58.745 1.00 1.00 H
ATOM 1931 HD22 ASN A 130 65.005 60.644 57.889 1.00 1.00 H
ATOM 1932 N ALA A 131 69.423 56.925 60.842 1.00 1.00 N
ATOM 1933 CA ALA A 131 69.546 56.253 62.131 1.00 1.00 C
ATOM 1934 C ALA A 131 68.651 57.011 63.127 1.00 1.00 C
ATOM 1935 O ALA A 131 68.409 58.205 62.946 1.00 1.00 O
ATOM 1936 CB ALA A 131 70.997 56.400 62.612 1.00 1.00 C
ATOM 1937 H ALA A 131 69.788 57.879 60.788 1.00 1.00 H
ATOM 1938 HA ALA A 131 69.266 55.201 62.069 1.00 1.00 H
ATOM 1939 HB1 ALA A 131 71.270 57.454 62.680 1.00 1.00 H
ATOM 1940 HB2 ALA A 131 71.109 55.937 63.593 1.00 1.00 H
ATOM 1941 HB3 ALA A 131 71.666 55.900 61.910 1.00 1.00 H
ATOM 1942 N GLY A 132 68.168 56.339 64.172 1.00 1.00 N
ATOM 1943 CA GLY A 132 67.590 56.946 65.371 1.00 1.00 C
ATOM 1944 C GLY A 132 68.296 56.351 66.583 1.00 1.00 C
ATOM 1945 O GLY A 132 68.484 55.133 66.652 1.00 1.00 O
ATOM 1946 H GLY A 132 68.353 55.330 64.216 1.00 1.00 H
ATOM 1947 HA2 GLY A 132 67.738 58.027 65.367 1.00 1.00 H
ATOM 1948 HA3 GLY A 132 66.522 56.746 65.444 1.00 1.00 H
ATOM 1949 N VAL A 133 68.725 57.211 67.503 1.00 1.00 N
ATOM 1950 CA VAL A 133 69.503 56.891 68.689 1.00 1.00 C

ATOM	1951	C	VAL	A	133	68.764	57.524	69.872	1.00	1.00	C
ATOM	1952	O	VAL	A	133	69.196	58.529	70.435	1.00	1.00	O
ATOM	1953	CB	VAL	A	133	70.987	57.334	68.517	1.00	1.00	C
ATOM	1954	CG1	VAL	A	133	71.831	56.874	69.715	1.00	1.00	C
ATOM	1955	CG2	VAL	A	133	71.615	56.718	67.254	1.00	1.00	C
ATOM	1956	H	VAL	A	133	68.520	58.196	67.327	1.00	1.00	H
ATOM	1957	HA	VAL	A	133	69.494	55.810	68.838	1.00	1.00	H
ATOM	1958	HB	VAL	A	133	71.039	58.421	68.439	1.00	1.00	H
ATOM	1959	HG11	VAL	A	133	72.863	57.199	69.587	1.00	1.00	H
ATOM	1960	HG12	VAL	A	133	71.454	57.315	70.638	1.00	1.00	H
ATOM	1961	HG13	VAL	A	133	71.800	55.788	69.801	1.00	1.00	H
ATOM	1962	HG21	VAL	A	133	72.673	56.967	67.200	1.00	1.00	H
ATOM	1963	HG22	VAL	A	133	71.504	55.633	67.275	1.00	1.00	H
ATOM	1964	HG23	VAL	A	133	71.126	57.112	66.364	1.00	1.00	H
ATOM	1965	N	GLY	A	134	67.625	56.934	70.238	1.00	1.00	N
ATOM	1966	CA	GLY	A	134	66.724	57.454	71.249	1.00	1.00	C
ATOM	1967	C	GLY	A	134	66.658	56.550	72.480	1.00	1.00	C
ATOM	1968	O	GLY	A	134	67.259	55.473	72.543	1.00	1.00	O
ATOM	1969	H	GLY	A	134	67.335	56.087	69.752	1.00	1.00	H
ATOM	1970	HA2	GLY	A	134	67.035	58.449	71.568	1.00	1.00	H
ATOM	1971	HA3	GLY	A	134	65.722	57.532	70.827	1.00	1.00	H
ATOM	1972	N	ALA	A	135	65.883	56.977	73.477	1.00	1.00	N
ATOM	1973	CA	ALA	A	135	65.591	56.242	74.698	1.00	1.00	C
ATOM	1974	C	ALA	A	135	64.130	56.478	75.065	1.00	1.00	C
ATOM	1975	O	ALA	A	135	63.653	57.607	74.977	1.00	1.00	O
ATOM	1976	CB	ALA	A	135	66.581	56.610	75.810	1.00	1.00	C
ATOM	1977	H	ALA	A	135	65.397	57.864	73.337	1.00	1.00	H
ATOM	1978	HA	ALA	A	135	65.713	55.178	74.495	1.00	1.00	H
ATOM	1979	HB1	ALA	A	135	66.361	56.023	76.702	1.00	1.00	H
ATOM	1980	HB2	ALA	A	135	67.598	56.391	75.481	1.00	1.00	H
ATOM	1981	HB3	ALA	A	135	66.499	57.674	76.034	1.00	1.00	H
ATOM	1982	N	PHE	A	136	63.433	55.409	75.447	1.00	1.00	N
ATOM	1983	CA	PHE	A	136	62.025	55.351	75.801	1.00	1.00	C
ATOM	1984	C	PHE	A	136	61.998	55.097	77.312	1.00	1.00	C
ATOM	1985	O	PHE	A	136	62.401	54.031	77.782	1.00	1.00	O
ATOM	1986	CB	PHE	A	136	61.438	54.193	74.968	1.00	1.00	C
ATOM	1987	CG	PHE	A	136	60.011	53.761	75.221	1.00	1.00	C
ATOM	1988	CD1	PHE	A	136	59.013	54.133	74.303	1.00	1.00	C
ATOM	1989	CD2	PHE	A	136	59.696	52.886	76.281	1.00	1.00	C
ATOM	1990	CE1	PHE	A	136	57.722	53.605	74.430	1.00	1.00	C
ATOM	1991	CE2	PHE	A	136	58.400	52.355	76.390	1.00	1.00	C
ATOM	1992	CZ	PHE	A	136	57.416	52.709	75.461	1.00	1.00	C
ATOM	1993	H	PHE	A	136	63.937	54.522	75.506	1.00	1.00	H
ATOM	1994	HA	PHE	A	136	61.514	56.282	75.554	1.00	1.00	H
ATOM	1995	HB2	PHE	A	136	61.521	54.480	73.918	1.00	1.00	H
ATOM	1996	HB3	PHE	A	136	62.074	53.319	75.078	1.00	1.00	H
ATOM	1997	HD1	PHE	A	136	59.243	54.796	73.481	1.00	1.00	H
ATOM	1998	HD2	PHE	A	136	60.450	52.589	76.994	1.00	1.00	H
ATOM	1999	HE1	PHE	A	136	56.962	53.871	73.724	1.00	1.00	H
ATOM	2000	HE2	PHE	A	136	58.153	51.660	77.173	1.00	1.00	H

ATOM	2001	HZ	PHE	A	136	56.428	52.286	75.533	1.00	1.00	H
ATOM	2002	N	TRP	A	137	61.614	56.120	78.075	1.00	1.00	N
ATOM	2003	CA	TRP	A	137	61.645	56.170	79.527	1.00	1.00	C
ATOM	2004	C	TRP	A	137	60.211	55.994	80.030	1.00	1.00	C
ATOM	2005	O	TRP	A	137	59.388	56.912	79.951	1.00	1.00	O
ATOM	2006	CB	TRP	A	137	62.150	57.562	79.965	1.00	1.00	C
ATOM	2007	CG	TRP	A	137	63.484	58.027	79.452	1.00	1.00	C
ATOM	2008	CD1	TRP	A	137	64.530	57.252	79.083	1.00	1.00	C
ATOM	2009	CD2	TRP	A	137	63.932	59.404	79.253	1.00	1.00	C
ATOM	2010	CE2	TRP	A	137	65.275	59.386	78.771	1.00	1.00	C
ATOM	2011	CE3	TRP	A	137	63.336	60.671	79.440	1.00	1.00	C
ATOM	2012	NE1	TRP	A	137	65.590	58.047	78.699	1.00	1.00	N
ATOM	2013	CZ2	TRP	A	137	65.992	60.561	78.496	1.00	1.00	C
ATOM	2014	CZ3	TRP	A	137	64.040	61.856	79.157	1.00	1.00	C
ATOM	2015	CH2	TRP	A	137	65.367	61.804	78.696	1.00	1.00	C
ATOM	2016	H	TRP	A	137	61.261	56.955	77.607	1.00	1.00	H
ATOM	2017	HA	TRP	A	137	62.295	55.394	79.935	1.00	1.00	H
ATOM	2018	HB2	TRP	A	137	61.424	58.313	79.657	1.00	1.00	H
ATOM	2019	HB3	TRP	A	137	62.190	57.580	81.054	1.00	1.00	H
ATOM	2020	HD1	TRP	A	137	64.529	56.171	79.095	1.00	1.00	H
ATOM	2021	HE1	TRP	A	137	66.486	57.675	78.419	1.00	1.00	H
ATOM	2022	HE3	TRP	A	137	62.325	60.728	79.814	1.00	1.00	H
ATOM	2023	HZ2	TRP	A	137	67.008	60.512	78.136	1.00	1.00	H
ATOM	2024	HZ3	TRP	A	137	63.561	62.813	79.304	1.00	1.00	H
ATOM	2025	HH2	TRP	A	137	65.904	62.720	78.490	1.00	1.00	H
ATOM	2026	N	ARG	A	138	59.914	54.814	80.575	1.00	1.00	N
ATOM	2027	CA	ARG	A	138	58.626	54.467	81.167	1.00	1.00	C
ATOM	2028	C	ARG	A	138	58.570	55.026	82.597	1.00	1.00	C
ATOM	2029	O	ARG	A	138	58.604	54.267	83.568	1.00	1.00	O
ATOM	2030	CB	ARG	A	138	58.515	52.931	81.219	1.00	1.00	C
ATOM	2031	CG	ARG	A	138	58.424	52.275	79.835	1.00	1.00	C
ATOM	2032	CD	ARG	A	138	58.943	50.836	79.867	1.00	1.00	C
ATOM	2033	NE	ARG	A	138	58.154	49.981	80.764	1.00	1.00	N
ATOM	2034	CZ	ARG	A	138	58.130	48.657	80.796	1.00	1.00	C
ATOM	2035	NH1	ARG	A	138	58.966	47.892	80.130	1.00	1.00	N1+
ATOM	2036	NH2	ARG	A	138	57.220	48.103	81.552	1.00	1.00	N1+
ATOM	2037	H	ARG	A	138	60.648	54.104	80.593	1.00	1.00	H
ATOM	2038	HA	ARG	A	138	57.806	54.887	80.588	1.00	1.00	H
ATOM	2039	HB2	ARG	A	138	59.395	52.542	81.729	1.00	1.00	H
ATOM	2040	HB3	ARG	A	138	57.633	52.646	81.793	1.00	1.00	H
ATOM	2041	HG2	ARG	A	138	57.389	52.286	79.492	1.00	1.00	H
ATOM	2042	HG3	ARG	A	138	59.033	52.830	79.124	1.00	1.00	H
ATOM	2043	HD2	ARG	A	138	58.913	50.425	78.857	1.00	1.00	H
ATOM	2044	HD3	ARG	A	138	59.980	50.860	80.194	1.00	1.00	H
ATOM	2045	HE	ARG	A	138	57.465	50.431	81.373	1.00	1.00	H
ATOM	2046	HH11	ARG	A	138	59.823	48.336	79.792	1.00	1.00	H
ATOM	2047	HH12	ARG	A	138	58.874	46.892	80.139	1.00	1.00	H
ATOM	2048	HH21	ARG	A	138	56.650	48.759	82.107	1.00	1.00	H
ATOM	2049	HH22	ARG	A	138	57.137	47.110	81.645	1.00	1.00	H
ATOM	2050	N	LEU	A	139	58.501	56.354	82.746	1.00	1.00	N

```
ATOM   2051  CA  LEU A 139      58.343  57.026  84.042  1.00  1.00           C
ATOM   2052  C   LEU A 139      56.963  56.678  84.626  1.00  1.00           C
ATOM   2053  O   LEU A 139      56.861  56.134  85.725  1.00  1.00           O
ATOM   2054  CB  LEU A 139      58.502  58.555  83.882  1.00  1.00           C
ATOM   2055  CG  LEU A 139      59.772  59.028  83.153  1.00  1.00           C
ATOM   2056  CD1 LEU A 139      59.828  60.553  83.175  1.00  1.00           C
ATOM   2057  CD2 LEU A 139      61.061  58.490  83.776  1.00  1.00           C
ATOM   2058  H   LEU A 139      58.552  56.915  81.904  1.00  1.00           H
ATOM   2059  HA  LEU A 139      59.108  56.660  84.729  1.00  1.00           H
ATOM   2060  HB2 LEU A 139      57.643  58.942  83.331  1.00  1.00           H
ATOM   2061  HB3 LEU A 139      58.483  58.999  84.879  1.00  1.00           H
ATOM   2062  HG  LEU A 139      59.731  58.706  82.113  1.00  1.00           H
ATOM   2063 HD11 LEU A 139      60.701  60.895  82.618  1.00  1.00           H
ATOM   2064 HD12 LEU A 139      58.928  60.958  82.712  1.00  1.00           H
ATOM   2065 HD13 LEU A 139      59.896  60.913  84.202  1.00  1.00           H
ATOM   2066 HD21 LEU A 139      61.925  58.912  83.262  1.00  1.00           H
ATOM   2067 HD22 LEU A 139      61.106  58.758  84.832  1.00  1.00           H
ATOM   2068 HD23 LEU A 139      61.101  57.406  83.676  1.00  1.00           H
ATOM   2069  N   ASN A 140      55.927  56.863  83.806  1.00  1.00           N
ATOM   2070  CA  ASN A 140      54.684  56.109  83.840  1.00  1.00           C
ATOM   2071  C   ASN A 140      54.790  55.169  82.623  1.00  1.00           C
ATOM   2072  O   ASN A 140      55.367  55.539  81.603  1.00  1.00           O
ATOM   2073  CB  ASN A 140      53.519  57.110  83.679  1.00  1.00           C
ATOM   2074  CG  ASN A 140      52.196  56.372  83.694  1.00  1.00           C
ATOM   2075  ND2 ASN A 140      51.560  56.230  84.843  1.00  1.00           N
ATOM   2076  OD1 ASN A 140      51.761  55.881  82.664  1.00  1.00           O
ATOM   2077  H   ASN A 140      56.109  57.345  82.935  1.00  1.00           H
ATOM   2078  HA  ASN A 140      54.584  55.540  84.766  1.00  1.00           H
ATOM   2079  HB2 ASN A 140      53.542  57.831  84.497  1.00  1.00           H
ATOM   2080  HB3 ASN A 140      53.614  57.651  82.741  1.00  1.00           H
ATOM   2081 HD21 ASN A 140      51.939  56.617  85.691  1.00  1.00           H
ATOM   2082 HD22 ASN A 140      50.680  55.740  84.832  1.00  1.00           H
ATOM   2083  N   ASP A 141      54.294  53.936  82.735  1.00  1.00           N
ATOM   2084  CA  ASP A 141      54.408  52.915  81.689  1.00  1.00           C
ATOM   2085  C   ASP A 141      53.365  53.080  80.582  1.00  1.00           C
ATOM   2086  O   ASP A 141      53.603  52.597  79.480  1.00  1.00           O
ATOM   2087  CB  ASP A 141      54.236  51.506  82.298  1.00  1.00           C
ATOM   2088  CG  ASP A 141      55.428  51.131  83.179  1.00  1.00           C
ATOM   2089  OD1 ASP A 141      56.264  50.331  82.703  1.00  1.00           O
ATOM   2090  OD2 ASP A 141      55.532  51.654  84.313  1.00  1.00           O1-
ATOM   2091  H   ASP A 141      53.921  53.647  83.627  1.00  1.00           H
ATOM   2092  HA  ASP A 141      55.392  52.978  81.223  1.00  1.00           H
ATOM   2093  HB2 ASP A 141      53.316  51.471  82.885  1.00  1.00           H
ATOM   2094  HB3 ASP A 141      54.151  50.774  81.492  1.00  1.00           H
ATOM   2095  N   ALA A 142      52.245  53.772  80.837  1.00  1.00           N
ATOM   2096  CA  ALA A 142      51.260  54.108  79.809  1.00  1.00           C
ATOM   2097  C   ALA A 142      51.685  55.407  79.108  1.00  1.00           C
ATOM   2098  O   ALA A 142      51.616  55.494  77.887  1.00  1.00           O
ATOM   2099  CB  ALA A 142      49.885  54.337  80.464  1.00  1.00           C
ATOM   2100  H   ALA A 142      52.123  54.185  81.754  1.00  1.00           H
```

```
ATOM 2101 HA ALA A 142 51.176 53.300 79.083 1.00 1.00 H
ATOM 2102 HB1 ALA A 142 49.146 54.525 79.684 1.00 1.00 H
ATOM 2103 HB2 ALA A 142 49.589 53.442 81.012 1.00 1.00 H
ATOM 2104 HB3 ALA A 142 49.901 55.189 81.142 1.00 1.00 H
ATOM 2105 N LEU A 143 52.139 56.403 79.881 1.00 1.00 N
ATOM 2106 CA LEU A 143 52.634 57.692 79.405 1.00 1.00 C
ATOM 2107 C LEU A 143 54.157 57.719 79.596 1.00 1.00 C
ATOM 2108 O LEU A 143 54.676 58.276 80.567 1.00 1.00 O
ATOM 2109 CB LEU A 143 51.947 58.821 80.210 1.00 1.00 C
ATOM 2110 CG LEU A 143 50.446 58.986 79.926 1.00 1.00 C
ATOM 2111 CD1 LEU A 143 49.891 60.105 80.814 1.00 1.00 C
ATOM 2112 CD2 LEU A 143 50.166 59.336 78.461 1.00 1.00 C
ATOM 2113 H LEU A 143 52.106 56.253 80.887 1.00 1.00 H
ATOM 2114 HA LEU A 143 52.427 57.817 78.343 1.00 1.00 H
ATOM 2115 HB2 LEU A 143 52.079 58.628 81.275 1.00 1.00 H
ATOM 2116 HB3 LEU A 143 52.447 59.762 79.981 1.00 1.00 H
ATOM 2117 HG LEU A 143 49.921 58.063 80.174 1.00 1.00 H
ATOM 2118 HD11 LEU A 143 50.065 59.859 81.862 1.00 1.00 H
ATOM 2119 HD12 LEU A 143 50.380 61.050 80.575 1.00 1.00 H
ATOM 2120 HD13 LEU A 143 48.818 60.202 80.652 1.00 1.00 H
ATOM 2121 HD21 LEU A 143 49.103 59.540 78.333 1.00 1.00 H
ATOM 2122 HD22 LEU A 143 50.740 60.213 78.164 1.00 1.00 H
ATOM 2123 HD23 LEU A 143 50.424 58.493 77.820 1.00 1.00 H
ATOM 2124 N SER A 144 54.873 57.084 78.667 1.00 1.00 N
ATOM 2125 CA SER A 144 56.330 57.026 78.641 1.00 1.00 C
ATOM 2126 C SER A 144 56.859 58.285 77.928 1.00 1.00 C
ATOM 2127 O SER A 144 56.211 58.799 77.019 1.00 1.00 O
ATOM 2128 CB SER A 144 56.733 55.786 77.815 1.00 1.00 C
ATOM 2129 OG SER A 144 56.453 54.603 78.532 1.00 1.00 O
ATOM 2130 H SER A 144 54.376 56.771 77.834 1.00 1.00 H
ATOM 2131 HA SER A 144 56.739 56.958 79.650 1.00 1.00 H
ATOM 2132 HB2 SER A 144 56.190 55.773 76.870 1.00 1.00 H
ATOM 2133 HB3 SER A 144 57.803 55.821 77.607 1.00 1.00 H
ATOM 2134 HG SER A 144 55.542 54.653 78.840 1.00 1.00 H
ATOM 2135 N LEU A 145 58.036 58.795 78.303 1.00 1.00 N
ATOM 2136 CA LEU A 145 58.709 59.868 77.566 1.00 1.00 C
ATOM 2137 C LEU A 145 59.737 59.244 76.614 1.00 1.00 C
ATOM 2138 O LEU A 145 60.310 58.195 76.913 1.00 1.00 O
ATOM 2139 CB LEU A 145 59.469 60.797 78.539 1.00 1.00 C
ATOM 2140 CG LEU A 145 58.574 61.549 79.543 1.00 1.00 C
ATOM 2141 CD1 LEU A 145 59.409 62.640 80.220 1.00 1.00 C
ATOM 2142 CD2 LEU A 145 57.351 62.213 78.903 1.00 1.00 C
ATOM 2143 H LEU A 145 58.564 58.293 79.019 1.00 1.00 H
ATOM 2144 HA LEU A 145 57.998 60.452 76.981 1.00 1.00 H
ATOM 2145 HB2 LEU A 145 60.196 60.208 79.097 1.00 1.00 H
ATOM 2146 HB3 LEU A 145 60.013 61.531 77.942 1.00 1.00 H
ATOM 2147 HG LEU A 145 58.229 60.851 80.305 1.00 1.00 H
ATOM 2148 HD11 LEU A 145 58.824 63.107 81.013 1.00 1.00 H
ATOM 2149 HD12 LEU A 145 60.303 62.200 80.660 1.00 1.00 H
ATOM 2150 HD13 LEU A 145 59.697 63.400 79.493 1.00 1.00 H
```

```
ATOM   2151 HD21 LEU A 145      56.815  62.797  79.650  1.00  1.00           H
ATOM   2152 HD22 LEU A 145      57.660  62.868  78.087  1.00  1.00           H
ATOM   2153 HD23 LEU A 145      56.680  61.445  78.523  1.00  1.00           H
ATOM   2154  N   ARG A 146      59.987  59.902  75.482  1.00  1.00           N
ATOM   2155  CA  ARG A 146      61.004  59.559  74.504  1.00  1.00           C
ATOM   2156  C   ARG A 146      61.883  60.786  74.258  1.00  1.00           C
ATOM   2157  O   ARG A 146      61.372  61.837  73.875  1.00  1.00           O
ATOM   2158  CB  ARG A 146      60.333  59.031  73.217  1.00  1.00           C
ATOM   2159  CG  ARG A 146      61.281  58.713  72.051  1.00  1.00           C
ATOM   2160  CD  ARG A 146      60.421  58.191  70.894  1.00  1.00           C
ATOM   2161  NE  ARG A 146      61.193  57.945  69.666  1.00  1.00           N
ATOM   2162  CZ  ARG A 146      60.721  57.364  68.568  1.00  1.00           C
ATOM   2163  NH1 ARG A 146      59.499  56.894  68.492  1.00  1.00           N1+
ATOM   2164  NH2 ARG A 146      61.463  57.226  67.506  1.00  1.00           N1+
ATOM   2165  H   ARG A 146      59.423  60.730  75.281  1.00  1.00           H
ATOM   2166  HA  ARG A 146      61.621  58.763  74.907  1.00  1.00           H
ATOM   2167  HB2 ARG A 146      59.778  58.127  73.475  1.00  1.00           H
ATOM   2168  HB3 ARG A 146      59.614  59.779  72.878  1.00  1.00           H
ATOM   2169  HG2 ARG A 146      61.801  59.618  71.735  1.00  1.00           H
ATOM   2170  HG3 ARG A 146      62.006  57.955  72.351  1.00  1.00           H
ATOM   2171  HD2 ARG A 146      59.942  57.263  71.211  1.00  1.00           H
ATOM   2172  HD3 ARG A 146      59.649  58.929  70.673  1.00  1.00           H
ATOM   2173  HE  ARG A 146      62.162  58.228  69.651  1.00  1.00           H
ATOM   2174 HH11 ARG A 146      58.893  56.901  69.284  1.00  1.00           H
ATOM   2175 HH12 ARG A 146      59.270  56.296  67.684  1.00  1.00           H
ATOM   2176 HH21 ARG A 146      62.457  57.488  67.507  1.00  1.00           H
ATOM   2177 HH22 ARG A 146      61.028  56.846  66.656  1.00  1.00           H
ATOM   2178  N   THR A 147      63.198  60.627  74.404  1.00  1.00           N
ATOM   2179  CA  THR A 147      64.176  61.475  73.727  1.00  1.00           C
ATOM   2180  C   THR A 147      64.566  60.684  72.468  1.00  1.00           C
ATOM   2181  O   THR A 147      64.732  59.461  72.547  1.00  1.00           O
ATOM   2182  CB  THR A 147      65.415  61.643  74.638  1.00  1.00           C
ATOM   2183  CG2 THR A 147      66.619  62.258  73.917  1.00  1.00           C
ATOM   2184  OG1 THR A 147      65.107  62.500  75.708  1.00  1.00           O
ATOM   2185  H   THR A 147      63.529  59.726  74.748  1.00  1.00           H
ATOM   2186  HA  THR A 147      63.763  62.449  73.460  1.00  1.00           H
ATOM   2187  HB  THR A 147      65.705  60.674  75.044  1.00  1.00           H
ATOM   2188  HG1 THR A 147      64.599  61.993  76.348  1.00  1.00           H
ATOM   2189 HG21 THR A 147      67.409  62.465  74.639  1.00  1.00           H
ATOM   2190 HG22 THR A 147      67.011  61.564  73.174  1.00  1.00           H
ATOM   2191 HG23 THR A 147      66.328  63.189  73.429  1.00  1.00           H
ATOM   2192  N   GLU A 148      64.685  61.368  71.332  1.00  1.00           N
ATOM   2193  CA  GLU A 148      65.087  60.834  70.046  1.00  1.00           C
ATOM   2194  C   GLU A 148      66.164  61.746  69.450  1.00  1.00           C
ATOM   2195  O   GLU A 148      66.184  62.957  69.700  1.00  1.00           O
ATOM   2196  CB  GLU A 148      63.835  60.625  69.167  1.00  1.00           C
ATOM   2197  CG  GLU A 148      64.081  60.318  67.690  1.00  1.00           C
ATOM   2198  CD  GLU A 148      64.769  58.980  67.516  1.00  1.00           C
ATOM   2199  OE1 GLU A 148      65.998  58.997  67.284  1.00  1.00           O
ATOM   2200  OE2 GLU A 148      64.054  57.958  67.624  1.00  1.00           O1-
```

ATOM 2201 H GLU A 148 64.417 62.356 71.319 1.00 1.00 H
ATOM 2202 HA GLU A 148 65.546 59.857 70.191 1.00 1.00 H
ATOM 2203 HB2 GLU A 148 63.247 59.812 69.596 1.00 1.00 H
ATOM 2204 HB3 GLU A 148 63.219 61.518 69.217 1.00 1.00 H
ATOM 2205 HG2 GLU A 148 63.121 60.286 67.174 1.00 1.00 H
ATOM 2206 HG3 GLU A 148 64.681 61.106 67.237 1.00 1.00 H
ATOM 2207 N ALA A 149 67.060 61.152 68.668 1.00 1.00 N
ATOM 2208 CA ALA A 149 68.197 61.792 68.042 1.00 1.00 C
ATOM 2209 C ALA A 149 68.392 61.102 66.698 1.00 1.00 C
ATOM 2210 O ALA A 149 68.969 60.018 66.624 1.00 1.00 O
ATOM 2211 CB ALA A 149 69.416 61.758 68.977 1.00 1.00 C
ATOM 2212 H ALA A 149 66.877 60.187 68.390 1.00 1.00 H
ATOM 2213 HA ALA A 149 67.953 62.839 67.858 1.00 1.00 H
ATOM 2214 HB1 ALA A 149 70.230 62.331 68.538 1.00 1.00 H
ATOM 2215 HB2 ALA A 149 69.156 62.203 69.938 1.00 1.00 H
ATOM 2216 HB3 ALA A 149 69.742 60.730 69.132 1.00 1.00 H
ATOM 2217 N ARG A 150 67.867 61.717 65.637 1.00 1.00 N
ATOM 2218 CA ARG A 150 67.994 61.240 64.271 1.00 1.00 C
ATOM 2219 C ARG A 150 69.334 61.722 63.699 1.00 1.00 C
ATOM 2220 O ARG A 150 69.787 62.830 63.998 1.00 1.00 O
ATOM 2221 CB ARG A 150 66.835 61.810 63.416 1.00 1.00 C
ATOM 2222 CG ARG A 150 65.505 61.049 63.592 1.00 1.00 C
ATOM 2223 CD ARG A 150 65.592 59.590 63.123 1.00 1.00 C
ATOM 2224 NE ARG A 150 64.269 58.975 62.921 1.00 1.00 N
ATOM 2225 CZ ARG A 150 64.043 57.697 62.648 1.00 1.00 C
ATOM 2226 NH1 ARG A 150 65.013 56.816 62.572 1.00 1.00 N1+
ATOM 2227 NH2 ARG A 150 62.821 57.303 62.433 1.00 1.00 N1+
ATOM 2228 H ARG A 150 67.467 62.649 65.769 1.00 1.00 H
ATOM 2229 HA ARG A 150 67.973 60.155 64.261 1.00 1.00 H
ATOM 2230 HB2 ARG A 150 66.665 62.852 63.680 1.00 1.00 H
ATOM 2231 HB3 ARG A 150 67.112 61.770 62.361 1.00 1.00 H
ATOM 2232 HG2 ARG A 150 65.211 61.063 64.641 1.00 1.00 H
ATOM 2233 HG3 ARG A 150 64.741 61.564 63.008 1.00 1.00 H
ATOM 2234 HD2 ARG A 150 66.139 59.558 62.181 1.00 1.00 H
ATOM 2235 HD3 ARG A 150 66.139 59.017 63.872 1.00 1.00 H
ATOM 2236 HE ARG A 150 63.456 59.583 62.874 1.00 1.00 H
ATOM 2237 HH11 ARG A 150 65.945 57.102 62.814 1.00 1.00 H
ATOM 2238 HH12 ARG A 150 64.807 55.888 62.256 1.00 1.00 H
ATOM 2239 HH21 ARG A 150 62.106 58.042 62.393 1.00 1.00 H
ATOM 2240 HH22 ARG A 150 62.593 56.339 62.210 1.00 1.00 H
ATOM 2241 N ALA A 151 69.955 60.890 62.864 1.00 1.00 N
ATOM 2242 CA ALA A 151 71.190 61.170 62.142 1.00 1.00 C
ATOM 2243 C ALA A 151 71.158 60.455 60.788 1.00 1.00 C
ATOM 2244 O ALA A 151 70.336 59.561 60.577 1.00 1.00 O
ATOM 2245 CB ALA A 151 72.420 60.833 63.003 1.00 1.00 C
ATOM 2246 H ALA A 151 69.515 59.990 62.680 1.00 1.00 H
ATOM 2247 HA ALA A 151 71.228 62.241 61.935 1.00 1.00 H
ATOM 2248 HB1 ALA A 151 73.332 61.080 62.460 1.00 1.00 H
ATOM 2249 HB2 ALA A 151 72.397 61.418 63.922 1.00 1.00 H
ATOM 2250 HB3 ALA A 151 72.427 59.771 63.250 1.00 1.00 H

```
ATOM   2251  N   THR A 152      72.056  60.839  59.882  1.00  1.00           N
ATOM   2252  CA  THR A 152      72.220  60.271  58.551  1.00  1.00           C
ATOM   2253  C   THR A 152      73.721  60.274  58.199  1.00  1.00           C
ATOM   2254  O   THR A 152      74.568  60.316  59.092  1.00  1.00           O
ATOM   2255  CB  THR A 152      71.211  60.925  57.556  1.00  1.00           C
ATOM   2256  CG2 THR A 152      71.504  62.399  57.262  1.00  1.00           C
ATOM   2257  OG1 THR A 152      71.231  60.212  56.336  1.00  1.00           O
ATOM   2258  H   THR A 152      72.751  61.520  60.161  1.00  1.00           H
ATOM   2259  HA  THR A 152      71.938  59.219  58.609  1.00  1.00           H
ATOM   2260  HB  THR A 152      70.207  60.847  57.967  1.00  1.00           H
ATOM   2261  HG1 THR A 152      70.709  59.390  56.463  1.00  1.00           H
ATOM   2262 HG21 THR A 152      70.759  62.778  56.562  1.00  1.00           H
ATOM   2263 HG22 THR A 152      71.446  62.979  58.182  1.00  1.00           H
ATOM   2264 HG23 THR A 152      72.494  62.513  56.821  1.00  1.00           H
ATOM   2265  N   TYR A 153      74.047  60.177  56.911  1.00  1.00           N
ATOM   2266  CA  TYR A 153      75.400  60.082  56.379  1.00  1.00           C
ATOM   2267  C   TYR A 153      75.747  61.440  55.756  1.00  1.00           C
ATOM   2268  O   TYR A 153      75.221  61.778  54.698  1.00  1.00           O
ATOM   2269  CB  TYR A 153      75.405  58.992  55.282  1.00  1.00           C
ATOM   2270  CG  TYR A 153      75.071  57.599  55.789  1.00  1.00           C
ATOM   2271  CD1 TYR A 153      73.731  57.163  55.846  1.00  1.00           C
ATOM   2272  CD2 TYR A 153      76.101  56.736  56.210  1.00  1.00           C
ATOM   2273  CE1 TYR A 153      73.428  55.876  56.324  1.00  1.00           C
ATOM   2274  CE2 TYR A 153      75.796  55.445  56.678  1.00  1.00           C
ATOM   2275  CZ  TYR A 153      74.459  55.016  56.732  1.00  1.00           C
ATOM   2276  OH  TYR A 153      74.156  53.764  57.179  1.00  1.00           O
ATOM   2277  H   TYR A 153      73.280  60.181  56.245  1.00  1.00           H
ATOM   2278  HA  TYR A 153      76.116  59.823  57.160  1.00  1.00           H
ATOM   2279  HB2 TYR A 153      74.689  59.253  54.501  1.00  1.00           H
ATOM   2280  HB3 TYR A 153      76.394  58.969  54.822  1.00  1.00           H
ATOM   2281  HD1 TYR A 153      72.932  57.819  55.529  1.00  1.00           H
ATOM   2282  HD2 TYR A 153      77.130  57.065  56.175  1.00  1.00           H
ATOM   2283  HE1 TYR A 153      72.401  55.545  56.381  1.00  1.00           H
ATOM   2284  HE2 TYR A 153      76.592  54.788  56.993  1.00  1.00           H
ATOM   2285  HH  TYR A 153      74.936  53.243  57.383  1.00  1.00           H
ATOM   2286  N   ASN A 154      76.611  62.218  56.417  1.00  1.00           N
ATOM   2287  CA  ASN A 154      77.007  63.569  55.990  1.00  1.00           C
ATOM   2288  C   ASN A 154      77.991  63.515  54.806  1.00  1.00           C
ATOM   2289  O   ASN A 154      77.973  64.397  53.951  1.00  1.00           O
ATOM   2290  CB  ASN A 154      77.755  64.300  57.132  1.00  1.00           C
ATOM   2291  CG  ASN A 154      76.978  64.327  58.446  1.00  1.00           C
ATOM   2292  ND2 ASN A 154      76.689  65.505  58.963  1.00  1.00           N
ATOM   2293  OD1 ASN A 154      76.633  63.295  59.011  1.00  1.00           O
ATOM   2294  H   ASN A 154      76.886  61.953  57.358  1.00  1.00           H
ATOM   2295  HA  ASN A 154      76.122  64.141  55.705  1.00  1.00           H
ATOM   2296  HB2 ASN A 154      78.722  63.830  57.321  1.00  1.00           H
ATOM   2297  HB3 ASN A 154      77.927  65.319  56.799  1.00  1.00           H
ATOM   2298 HD21 ASN A 154      76.978  66.367  58.481  1.00  1.00           H
ATOM   2299 HD22 ASN A 154      76.162  65.529  59.829  1.00  1.00           H
ATOM   2300  N   ALA A 155      78.824  62.463  54.788  1.00  1.00           N
```

ATOM 2301 CA ALA A 155 80.015 62.222 53.973 1.00 1.00 C
ATOM 2302 C ALA A 155 81.288 62.645 54.715 1.00 1.00 C
ATOM 2303 O ALA A 155 81.237 63.411 55.678 1.00 1.00 O
ATOM 2304 CB ALA A 155 79.926 62.726 52.522 1.00 1.00 C
ATOM 2305 H ALA A 155 78.674 61.802 55.531 1.00 1.00 H
ATOM 2306 HA ALA A 155 80.076 61.136 53.887 1.00 1.00 H
ATOM 2307 HB1 ALA A 155 78.979 62.416 52.080 1.00 1.00 H
ATOM 2308 HB2 ALA A 155 80.004 63.813 52.497 1.00 1.00 H
ATOM 2309 HB3 ALA A 155 80.744 62.305 51.937 1.00 1.00 H
ATOM 2310 N ASP A 156 82.421 62.087 54.292 1.00 1.00 N
ATOM 2311 CA ASP A 156 83.765 62.291 54.822 1.00 1.00 C
ATOM 2312 C ASP A 156 84.209 63.748 54.600 1.00 1.00 C
ATOM 2313 O ASP A 156 84.300 64.189 53.454 1.00 1.00 O
ATOM 2314 CB ASP A 156 84.763 61.317 54.132 1.00 1.00 C
ATOM 2315 CG ASP A 156 84.257 59.870 53.951 1.00 1.00 C
ATOM 2316 OD1 ASP A 156 84.875 58.941 54.521 1.00 1.00 O
ATOM 2317 OD2 ASP A 156 83.250 59.709 53.217 1.00 1.00 O1-
ATOM 2318 H ASP A 156 82.360 61.369 53.571 1.00 1.00 H
ATOM 2319 HA ASP A 156 83.750 62.071 55.890 1.00 1.00 H
ATOM 2320 HB2 ASP A 156 85.003 61.699 53.138 1.00 1.00 H
ATOM 2321 HB3 ASP A 156 85.690 61.312 54.708 1.00 1.00 H
ATOM 2322 N GLU A 157 84.473 64.460 55.706 1.00 1.00 N
ATOM 2323 CA GLU A 157 84.876 65.867 55.832 1.00 1.00 C
ATOM 2324 C GLU A 157 83.650 66.780 56.042 1.00 1.00 C
ATOM 2325 O GLU A 157 83.368 67.664 55.233 1.00 1.00 O
ATOM 2326 CB GLU A 157 85.944 66.316 54.794 1.00 1.00 C
ATOM 2327 CG GLU A 157 86.717 67.569 55.212 1.00 1.00 C
ATOM 2328 CD GLU A 157 87.676 67.258 56.349 1.00 1.00 C
ATOM 2329 OE1 GLU A 157 88.843 66.938 56.037 1.00 1.00 O
ATOM 2330 OE2 GLU A 157 87.226 67.348 57.512 1.00 1.00 O1-
ATOM 2331 H GLU A 157 84.361 63.954 56.568 1.00 1.00 H
ATOM 2332 HA GLU A 157 85.394 65.905 56.789 1.00 1.00 H
ATOM 2333 HB2 GLU A 157 86.658 65.506 54.639 1.00 1.00 H
ATOM 2334 HB3 GLU A 157 85.456 66.522 53.842 1.00 1.00 H
ATOM 2335 HG2 GLU A 157 87.292 67.927 54.358 1.00 1.00 H
ATOM 2336 HG3 GLU A 157 86.026 68.355 55.515 1.00 1.00 H
ATOM 2337 N GLU A 158 82.891 66.531 57.119 1.00 1.00 N
ATOM 2338 CA GLU A 158 81.680 67.248 57.512 1.00 1.00 C
ATOM 2339 C GLU A 158 81.579 67.210 59.052 1.00 1.00 C
ATOM 2340 O GLU A 158 82.271 66.434 59.719 1.00 1.00 O
ATOM 2341 CB GLU A 158 80.456 66.666 56.762 1.00 1.00 C
ATOM 2342 CG GLU A 158 79.465 67.717 56.241 1.00 1.00 C
ATOM 2343 CD GLU A 158 78.562 68.273 57.330 1.00 1.00 C
ATOM 2344 OE1 GLU A 158 77.482 67.681 57.541 1.00 1.00 O
ATOM 2345 OE2 GLU A 158 78.967 69.278 57.954 1.00 1.00 O1-
ATOM 2346 H GLU A 158 83.201 65.811 57.755 1.00 1.00 H
ATOM 2347 HA GLU A 158 81.795 68.292 57.217 1.00 1.00 H
ATOM 2348 HB2 GLU A 158 80.804 66.118 55.886 1.00 1.00 H
ATOM 2349 HB3 GLU A 158 79.951 65.961 57.419 1.00 1.00 H
ATOM 2350 HG2 GLU A 158 80.010 68.531 55.762 1.00 1.00 H

ATOM 2351 HG3 GLU A 158 78.835 67.249 55.484 1.00 1.00 H
ATOM 2352 N PHE A 159 80.730 68.063 59.625 1.00 1.00 N
ATOM 2353 CA PHE A 159 80.536 68.269 61.051 1.00 1.00 C
ATOM 2354 C PHE A 159 79.444 67.312 61.566 1.00 1.00 C
ATOM 2355 O PHE A 159 78.259 67.649 61.602 1.00 1.00 O
ATOM 2356 CB PHE A 159 80.117 69.744 61.248 1.00 1.00 C
ATOM 2357 CG PHE A 159 79.930 70.151 62.701 1.00 1.00 C
ATOM 2358 CD1 PHE A 159 78.639 70.221 63.263 1.00 1.00 C
ATOM 2359 CD2 PHE A 159 81.051 70.456 63.497 1.00 1.00 C
ATOM 2360 CE1 PHE A 159 78.472 70.581 64.612 1.00 1.00 C
ATOM 2361 CE2 PHE A 159 80.884 70.824 64.845 1.00 1.00 C
ATOM 2362 CZ PHE A 159 79.595 70.883 65.403 1.00 1.00 C
ATOM 2363 H PHE A 159 80.157 68.641 59.003 1.00 1.00 H
ATOM 2364 HA PHE A 159 81.470 68.088 61.585 1.00 1.00 H
ATOM 2365 HB2 PHE A 159 80.888 70.383 60.814 1.00 1.00 H
ATOM 2366 HB3 PHE A 159 79.194 69.938 60.698 1.00 1.00 H
ATOM 2367 HD1 PHE A 159 77.773 69.988 62.660 1.00 1.00 H
ATOM 2368 HD2 PHE A 159 82.044 70.409 63.074 1.00 1.00 H
ATOM 2369 HE1 PHE A 159 77.481 70.623 65.041 1.00 1.00 H
ATOM 2370 HE2 PHE A 159 81.747 71.059 65.453 1.00 1.00 H
ATOM 2371 HZ PHE A 159 79.464 71.158 66.439 1.00 1.00 H
ATOM 2372 N TRP A 160 79.874 66.113 61.984 1.00 1.00 N
ATOM 2373 CA TRP A 160 79.079 65.024 62.568 1.00 1.00 C
ATOM 2374 C TRP A 160 78.087 65.502 63.650 1.00 1.00 C
ATOM 2375 O TRP A 160 78.489 66.124 64.637 1.00 1.00 O
ATOM 2376 CB TRP A 160 80.017 63.927 63.118 1.00 1.00 C
ATOM 2377 CG TRP A 160 80.860 63.338 62.024 1.00 1.00 C
ATOM 2378 CD1 TRP A 160 82.167 63.604 61.795 1.00 1.00 C
ATOM 2379 CD2 TRP A 160 80.421 62.522 60.891 1.00 1.00 C
ATOM 2380 CE2 TRP A 160 81.518 62.366 59.991 1.00 1.00 C
ATOM 2381 CE3 TRP A 160 79.193 61.933 60.512 1.00 1.00 C
ATOM 2382 NE1 TRP A 160 82.563 63.018 60.611 1.00 1.00 N
ATOM 2383 CZ2 TRP A 160 81.399 61.689 58.769 1.00 1.00 C
ATOM 2384 CZ3 TRP A 160 79.069 61.231 59.297 1.00 1.00 C
ATOM 2385 CH2 TRP A 160 80.164 61.115 58.422 1.00 1.00 C
ATOM 2386 H TRP A 160 80.851 65.914 61.823 1.00 1.00 H
ATOM 2387 HA TRP A 160 78.496 64.584 61.758 1.00 1.00 H
ATOM 2388 HB2 TRP A 160 80.653 64.347 63.900 1.00 1.00 H
ATOM 2389 HB3 TRP A 160 79.415 63.131 63.558 1.00 1.00 H
ATOM 2390 HD1 TRP A 160 82.793 64.228 62.418 1.00 1.00 H
ATOM 2391 HE1 TRP A 160 83.489 63.132 60.227 1.00 1.00 H
ATOM 2392 HE3 TRP A 160 78.330 62.035 61.154 1.00 1.00 H
ATOM 2393 HZ2 TRP A 160 82.244 61.618 58.100 1.00 1.00 H
ATOM 2394 HZ3 TRP A 160 78.117 60.793 59.031 1.00 1.00 H
ATOM 2395 HH2 TRP A 160 80.057 60.589 57.485 1.00 1.00 H
ATOM 2396 N ASN A 161 76.794 65.231 63.453 1.00 1.00 N
ATOM 2397 CA ASN A 161 75.674 65.936 64.076 1.00 1.00 C
ATOM 2398 C ASN A 161 74.519 64.997 64.454 1.00 1.00 C
ATOM 2399 O ASN A 161 74.523 63.814 64.121 1.00 1.00 O
ATOM 2400 CB ASN A 161 75.234 67.138 63.192 1.00 1.00 C

```
ATOM   2401  CG  ASN A 161      74.998  66.815  61.713  1.00  1.00           C
ATOM   2402  ND2 ASN A 161      74.852  67.843  60.896  1.00  1.00           N
ATOM   2403  OD1 ASN A 161      74.960  65.670  61.279  1.00  1.00           O
ATOM   2404  H   ASN A 161      76.546  64.665  62.650  1.00  1.00           H
ATOM   2405  HA  ASN A 161      76.026  66.359  65.018  1.00  1.00           H
ATOM   2406  HB2 ASN A 161      74.339  67.608  63.597  1.00  1.00           H
ATOM   2407  HB3 ASN A 161      76.035  67.878  63.231  1.00  1.00           H
ATOM   2408 HD21 ASN A 161      74.961  68.786  61.230  1.00  1.00           H
ATOM   2409 HD22 ASN A 161      74.773  67.641  59.910  1.00  1.00           H
ATOM   2410  N   TYR A 162      73.510  65.554  65.131  1.00  1.00           N
ATOM   2411  CA  TYR A 162      72.230  64.932  65.451  1.00  1.00           C
ATOM   2412  C   TYR A 162      71.117  65.947  65.167  1.00  1.00           C
ATOM   2413  O   TYR A 162      71.336  67.155  65.267  1.00  1.00           O
ATOM   2414  CB  TYR A 162      72.180  64.475  66.925  1.00  1.00           C
ATOM   2415  CG  TYR A 162      72.839  63.139  67.231  1.00  1.00           C
ATOM   2416  CD1 TYR A 162      72.260  61.951  66.741  1.00  1.00           C
ATOM   2417  CD2 TYR A 162      73.979  63.067  68.058  1.00  1.00           C
ATOM   2418  CE1 TYR A 162      72.800  60.700  67.087  1.00  1.00           C
ATOM   2419  CE2 TYR A 162      74.523  61.812  68.401  1.00  1.00           C
ATOM   2420  CZ  TYR A 162      73.930  60.631  67.921  1.00  1.00           C
ATOM   2421  OH  TYR A 162      74.441  59.417  68.277  1.00  1.00           O
ATOM   2422  H   TYR A 162      73.580  66.538  65.344  1.00  1.00           H
ATOM   2423  HA  TYR A 162      72.071  64.067  64.806  1.00  1.00           H
ATOM   2424  HB2 TYR A 162      72.613  65.253  67.555  1.00  1.00           H
ATOM   2425  HB3 TYR A 162      71.134  64.381  67.219  1.00  1.00           H
ATOM   2426  HD1 TYR A 162      71.380  61.997  66.115  1.00  1.00           H
ATOM   2427  HD2 TYR A 162      74.434  63.970  68.441  1.00  1.00           H
ATOM   2428  HE1 TYR A 162      72.330  59.797  66.724  1.00  1.00           H
ATOM   2429  HE2 TYR A 162      75.392  61.752  69.041  1.00  1.00           H
ATOM   2430  HH  TYR A 162      73.993  58.693  67.834  1.00  1.00           H
ATOM   2431  N   THR A 163      69.914  65.453  64.881  1.00  1.00           N
ATOM   2432  CA  THR A 163      68.678  66.204  64.708  1.00  1.00           C
ATOM   2433  C   THR A 163      67.694  65.647  65.755  1.00  1.00           C
ATOM   2434  O   THR A 163      67.397  64.453  65.765  1.00  1.00           O
ATOM   2435  CB  THR A 163      68.257  66.121  63.214  1.00  1.00           C
ATOM   2436  CG2 THR A 163      69.292  66.757  62.280  1.00  1.00           C
ATOM   2437  OG1 THR A 163      68.063  64.792  62.785  1.00  1.00           O
ATOM   2438  H   THR A 163      69.850  64.439  64.807  1.00  1.00           H
ATOM   2439  HA  THR A 163      68.856  67.255  64.940  1.00  1.00           H
ATOM   2440  HB  THR A 163      67.322  66.660  63.086  1.00  1.00           H
ATOM   2441  HG1 THR A 163      68.803  64.248  63.089  1.00  1.00           H
ATOM   2442 HG21 THR A 163      70.218  66.181  62.277  1.00  1.00           H
ATOM   2443 HG22 THR A 163      68.889  66.787  61.267  1.00  1.00           H
ATOM   2444 HG23 THR A 163      69.504  67.775  62.606  1.00  1.00           H
ATOM   2445  N   ALA A 164      67.304  66.480  66.729  1.00  1.00           N
ATOM   2446  CA  ALA A 164      66.606  66.065  67.945  1.00  1.00           C
ATOM   2447  C   ALA A 164      65.085  66.046  67.740  1.00  1.00           C
ATOM   2448  O   ALA A 164      64.542  66.855  66.985  1.00  1.00           O
ATOM   2449  CB  ALA A 164      66.900  67.105  69.040  1.00  1.00           C
ATOM   2450  H   ALA A 164      67.565  67.453  66.656  1.00  1.00           H
```

ATOM 2451 HA ALA A 164 66.958 65.084 68.268 1.00 1.00 H
ATOM 2452 HB1 ALA A 164 66.547 68.091 68.733 1.00 1.00 H
ATOM 2453 HB2 ALA A 164 66.397 66.818 69.965 1.00 1.00 H
ATOM 2454 HB3 ALA A 164 67.974 67.148 69.226 1.00 1.00 H
ATOM 2455 N LEU A 165 64.408 65.132 68.439 1.00 1.00 N
ATOM 2456 CA LEU A 165 62.961 65.047 68.575 1.00 1.00 C
ATOM 2457 C LEU A 165 62.678 64.633 70.026 1.00 1.00 C
ATOM 2458 O LEU A 165 63.339 63.734 70.550 1.00 1.00 O
ATOM 2459 CB LEU A 165 62.308 64.087 67.543 1.00 1.00 C
ATOM 2460 CG LEU A 165 62.595 64.310 66.039 1.00 1.00 C
ATOM 2461 CD1 LEU A 165 63.858 63.594 65.546 1.00 1.00 C
ATOM 2462 CD2 LEU A 165 61.452 63.752 65.191 1.00 1.00 C
ATOM 2463 H LEU A 165 64.946 64.476 69.007 1.00 1.00 H
ATOM 2464 HA LEU A 165 62.540 66.041 68.417 1.00 1.00 H
ATOM 2465 HB2 LEU A 165 62.580 63.064 67.788 1.00 1.00 H
ATOM 2466 HB3 LEU A 165 61.232 64.170 67.699 1.00 1.00 H
ATOM 2467 HG LEU A 165 62.678 65.376 65.840 1.00 1.00 H
ATOM 2468 HD11 LEU A 165 63.728 62.518 65.640 1.00 1.00 H
ATOM 2469 HD12 LEU A 165 64.033 63.846 64.501 1.00 1.00 H
ATOM 2470 HD13 LEU A 165 64.725 63.899 66.123 1.00 1.00 H
ATOM 2471 HD21 LEU A 165 61.642 63.942 64.134 1.00 1.00 H
ATOM 2472 HD22 LEU A 165 61.367 62.676 65.343 1.00 1.00 H
ATOM 2473 HD23 LEU A 165 60.520 64.236 65.460 1.00 1.00 H
ATOM 2474 N ALA A 166 61.719 65.280 70.695 1.00 1.00 N
ATOM 2475 CA ALA A 166 61.230 64.879 72.016 1.00 1.00 C
ATOM 2476 C ALA A 166 59.789 64.386 71.848 1.00 1.00 C
ATOM 2477 O ALA A 166 59.006 65.039 71.163 1.00 1.00 O
ATOM 2478 CB ALA A 166 61.259 66.098 72.952 1.00 1.00 C
ATOM 2479 H ALA A 166 61.234 66.038 70.215 1.00 1.00 H
ATOM 2480 HA ALA A 166 61.845 64.086 72.443 1.00 1.00 H
ATOM 2481 HB1 ALA A 166 60.847 65.823 73.923 1.00 1.00 H
ATOM 2482 HB2 ALA A 166 62.290 66.427 73.087 1.00 1.00 H
ATOM 2483 HB3 ALA A 166 60.678 66.917 72.533 1.00 1.00 H
ATOM 2484 N GLY A 167 59.430 63.247 72.439 1.00 1.00 N
ATOM 2485 CA GLY A 167 58.094 62.668 72.355 1.00 1.00 C
ATOM 2486 C GLY A 167 57.537 62.342 73.740 1.00 1.00 C
ATOM 2487 O GLY A 167 58.264 61.853 74.607 1.00 1.00 O
ATOM 2488 H GLY A 167 60.121 62.763 73.016 1.00 1.00 H
ATOM 2489 HA2 GLY A 167 57.408 63.352 71.854 1.00 1.00 H
ATOM 2490 HA3 GLY A 167 58.133 61.748 71.772 1.00 1.00 H
ATOM 2491 N LEU A 168 56.229 62.546 73.933 1.00 1.00 N
ATOM 2492 CA LEU A 168 55.426 61.848 74.935 1.00 1.00 C
ATOM 2493 C LEU A 168 54.858 60.645 74.174 1.00 1.00 C
ATOM 2494 O LEU A 168 54.328 60.819 73.077 1.00 1.00 O
ATOM 2495 CB LEU A 168 54.265 62.772 75.381 1.00 1.00 C
ATOM 2496 CG LEU A 168 53.243 62.114 76.329 1.00 1.00 C
ATOM 2497 CD1 LEU A 168 53.888 61.614 77.621 1.00 1.00 C
ATOM 2498 CD2 LEU A 168 52.159 63.134 76.692 1.00 1.00 C
ATOM 2499 H LEU A 168 55.711 63.015 73.200 1.00 1.00 H
ATOM 2500 HA LEU A 168 56.032 61.532 75.786 1.00 1.00 H

ATOM 2501 HB2 LEU A 168 54.696 63.642 75.878 1.00 1.00 H
ATOM 2502 HB3 LEU A 168 53.734 63.124 74.495 1.00 1.00 H
ATOM 2503 HG LEU A 168 52.768 61.272 75.826 1.00 1.00 H
ATOM 2504 HD11 LEU A 168 54.578 60.799 77.413 1.00 1.00 H
ATOM 2505 HD12 LEU A 168 54.414 62.431 78.115 1.00 1.00 H
ATOM 2506 HD13 LEU A 168 53.113 61.239 78.286 1.00 1.00 H
ATOM 2507 HD21 LEU A 168 51.667 63.481 75.783 1.00 1.00 H
ATOM 2508 HD22 LEU A 168 51.411 62.667 77.334 1.00 1.00 H
ATOM 2509 HD23 LEU A 168 52.596 63.987 77.213 1.00 1.00 H
ATOM 2510 N ASN A 169 55.026 59.433 74.693 1.00 1.00 N
ATOM 2511 CA ASN A 169 54.654 58.190 74.037 1.00 1.00 C
ATOM 2512 C ASN A 169 53.533 57.560 74.864 1.00 1.00 C
ATOM 2513 O ASN A 169 53.723 57.255 76.044 1.00 1.00 O
ATOM 2514 CB ASN A 169 55.909 57.294 73.955 1.00 1.00 C
ATOM 2515 CG ASN A 169 55.636 56.053 73.100 1.00 1.00 C
ATOM 2516 ND2 ASN A 169 56.349 55.880 71.989 1.00 1.00 N
ATOM 2517 OD1 ASN A 169 54.808 55.223 73.451 1.00 1.00 O
ATOM 2518 H ASN A 169 55.457 59.340 75.610 1.00 1.00 H
ATOM 2519 HA ASN A 169 54.296 58.388 73.030 1.00 1.00 H
ATOM 2520 HB2 ASN A 169 56.725 57.864 73.510 1.00 1.00 H
ATOM 2521 HB3 ASN A 169 56.214 56.974 74.950 1.00 1.00 H
ATOM 2522 HD21 ASN A 169 56.987 56.593 71.686 1.00 1.00 H
ATOM 2523 HD22 ASN A 169 56.248 55.008 71.489 1.00 1.00 H
ATOM 2524 N VAL A 170 52.370 57.394 74.232 1.00 1.00 N
ATOM 2525 CA VAL A 170 51.206 56.736 74.792 1.00 1.00 C
ATOM 2526 C VAL A 170 51.335 55.269 74.348 1.00 1.00 C
ATOM 2527 O VAL A 170 51.381 54.968 73.151 1.00 1.00 O
ATOM 2528 CB VAL A 170 49.906 57.376 74.228 1.00 1.00 C
ATOM 2529 CG1 VAL A 170 48.706 56.819 75.004 1.00 1.00 C
ATOM 2530 CG2 VAL A 170 49.872 58.909 74.341 1.00 1.00 C
ATOM 2531 H VAL A 170 52.363 57.593 73.231 1.00 1.00 H
ATOM 2532 HA VAL A 170 51.212 56.824 75.877 1.00 1.00 H
ATOM 2533 HB VAL A 170 49.794 57.115 73.178 1.00 1.00 H
ATOM 2534 HG11 VAL A 170 47.779 57.212 74.588 1.00 1.00 H
ATOM 2535 HG12 VAL A 170 48.684 55.733 74.936 1.00 1.00 H
ATOM 2536 HG13 VAL A 170 48.776 57.100 76.055 1.00 1.00 H
ATOM 2537 HG21 VAL A 170 48.909 59.283 73.994 1.00 1.00 H
ATOM 2538 HG22 VAL A 170 50.027 59.209 75.377 1.00 1.00 H
ATOM 2539 HG23 VAL A 170 50.655 59.349 73.724 1.00 1.00 H
ATOM 2540 N VAL A 171 51.443 54.377 75.329 1.00 1.00 N
ATOM 2541 CA VAL A 171 51.580 52.943 75.153 1.00 1.00 C
ATOM 2542 C VAL A 171 50.165 52.364 75.303 1.00 1.00 C
ATOM 2543 O VAL A 171 49.547 52.483 76.364 1.00 1.00 O
ATOM 2544 CB VAL A 171 52.539 52.397 76.245 1.00 1.00 C
ATOM 2545 CG1 VAL A 171 52.756 50.896 76.003 1.00 1.00 C
ATOM 2546 CG2 VAL A 171 53.901 53.100 76.181 1.00 1.00 C
ATOM 2547 H VAL A 171 51.456 54.741 76.285 1.00 1.00 H
ATOM 2548 HA VAL A 171 51.985 52.718 74.166 1.00 1.00 H
ATOM 2549 HB VAL A 171 52.108 52.543 77.233 1.00 1.00 H
ATOM 2550 HG11 VAL A 171 51.818 50.357 76.146 1.00 1.00 H

```
ATOM   2551 HG12 VAL A 171      53.115  50.723  74.988  1.00  1.00           H
ATOM   2552 HG13 VAL A 171      53.483  50.510  76.717  1.00  1.00           H
ATOM   2553 HG21 VAL A 171      54.589  52.624  76.878  1.00  1.00           H
ATOM   2554 HG22 VAL A 171      54.297  53.059  75.167  1.00  1.00           H
ATOM   2555 HG23 VAL A 171      53.799  54.145  76.476  1.00  1.00           H
ATOM   2556  N   LEU A 172      49.663  51.778  74.217  1.00  1.00           N
ATOM   2557  CA  LEU A 172      48.372  51.118  74.081  1.00  1.00           C
ATOM   2558  C   LEU A 172      48.643  49.697  73.570  1.00  1.00           C
ATOM   2559  O   LEU A 172      49.820  49.313  73.359  1.00  1.00           O
ATOM   2560  CB  LEU A 172      47.486  51.904  73.083  1.00  1.00           C
ATOM   2561  CG  LEU A 172      47.251  53.385  73.437  1.00  1.00           C
ATOM   2562  CD1 LEU A 172      46.366  54.035  72.369  1.00  1.00           C
ATOM   2563  CD2 LEU A 172      46.568  53.574  74.796  1.00  1.00           C
ATOM   2564  H   LEU A 172      50.271  51.707  73.401  1.00  1.00           H
ATOM   2565  HA  LEU A 172      47.872  51.041  75.046  1.00  1.00           H
ATOM   2566  HB2 LEU A 172      47.956  51.859  72.102  1.00  1.00           H
ATOM   2567  HB3 LEU A 172      46.522  51.399  73.012  1.00  1.00           H
ATOM   2568  HG  LEU A 172      48.204  53.913  73.448  1.00  1.00           H
ATOM   2569 HD11 LEU A 172      46.242  55.096  72.589  1.00  1.00           H
ATOM   2570 HD12 LEU A 172      46.839  53.932  71.393  1.00  1.00           H
ATOM   2571 HD13 LEU A 172      45.388  53.552  72.348  1.00  1.00           H
ATOM   2572 HD21 LEU A 172      47.208  53.208  75.596  1.00  1.00           H
ATOM   2573 HD22 LEU A 172      46.371  54.632  74.970  1.00  1.00           H
ATOM   2574 HD23 LEU A 172      45.628  53.025  74.818  1.00  1.00           H
END
```
