# Supplementary material for: AOA-2 Derivatives as Outer Membrane Protein A Inhibitors for Treatment of Gram-Negative Bacilli Infections
Source: Front Microbiol. 2021 Feb 12;12:634323. doi: 10.3389/fmicb.2021.634323 (PMC7907166; doi:10.3389/fmicb.2021.634323)
Supplement: Supplementary file 2 [file Data_Sheet_2.PDF]

AOA-2

```
80 86  0  0  0  0  0  0  0  0999 V2000
  66.8025   68.1510   58.8007 N   0  0  0  0  0  0  0  0  0  0  0  0
  65.9473   68.5988   58.4860 H   0  0  0  0  0  0  0  0  0  0  0  0
  66.9649   66.7287   58.5477 C   0  0  0  0  0  0  0  0  0  0  0  0
  66.1042   66.2932   57.3463 C   0  0  0  0  0  0  0  0  0  0  0  0
  65.7260   65.1293   57.2076 O   0  0  0  0  0  0  0  0  0  0  0  0
  65.9184   67.2744   56.3855 N   0  0  0  0  0  0  0  0  0  0  0  0
  66.3312   68.2092   56.5041 H   0  0  0  0  0  0  0  0  0  0  0  0
  65.1376   67.0293   55.1718 C   0  0  0  0  0  0  0  0  0  0  0  0
  65.9132   67.6219   53.9837 C   0  0  0  0  0  0  0  0  0  0  0  0
  66.2818   68.8042   54.0027 O   0  0  0  0  0  0  0  0  0  0  0  0
  66.1860   66.8067   52.8996 N   0  0  0  0  0  0  0  0  0  0  0  0
  65.7568   65.4241   52.7244 C   0  0  0  0  0  0  0  0  0  0  0  0
  65.7374   65.2340   51.2183 C   0  0  0  0  0  0  0  0  0  0  0  0
  66.9106   66.0805   50.7536 C   0  0  0  0  0  0  0  0  0  0  0  0
  66.9021   67.2881   51.7050 C   0  0  0  0  0  0  0  0  0  0  0  0
  68.3389   67.7880   51.9710 C   0  0  0  0  0  0  0  0  0  0  0  0
  69.3518   67.1264   51.7628 O   0  0  0  0  0  0  0  0  0  0  0  0
  68.3805   69.0778   52.4668 N   0  0  0  0  0  0  0  0  0  0  0  0
  67.4832   69.5025   52.7152 H   0  0  0  0  0  0  0  0  0  0  0  0
  69.6243   69.8100   52.6834 C   0  0  0  0  0  0  0  0  0  0  0  0
  69.4426   70.9360   53.7137 C   0  0  0  0  0  0  0  0  0  0  0  0
  70.1700   71.9339   53.7366 O   0  0  0  0  0  0  0  0  0  0  0  0
  68.4802   70.6785   54.6685 N   0  0  0  0  0  0  0  0  0  0  0  0
  67.9416   69.8075   54.6297 H   0  0  0  0  0  0  0  0  0  0  0  0
  68.1599   71.6179   55.7414 C   0  0  0  0  0  0  0  0  0  0  0  0
  67.5606   70.8020   56.9031 C   0  0  0  0  0  0  0  0  0  0  0  0
  66.6610   69.9764   56.6955 O   0  0  0  0  0  0  0  0  0  0  0  0
  68.0550   71.0244   58.1754 N   0  0  0  0  0  0  0  0  0  0  0  0
  69.1878   71.8674   58.5382 C   0  0  0  0  0  0  0  0  0  0  0  0
  69.4256   71.6016   60.0163 C   0  0  0  0  0  0  0  0  0  0  0  0
  68.0474   71.2016   60.5299 C   0  0  0  0  0  0  0  0  0  0  0  0
  67.4365   70.4300   59.3618 C   0  0  0  0  0  0  0  0  0  0  0  0
  67.7649   68.9270   59.4139 C   0  0  0  0  0  0  0  0  0  0  0  0
  68.8114   68.4672   59.8606 O   0  0  0  0  0  0  0  0  0  0  0  0
  66.6755   65.8661   59.7847 C   0  0  0  0  0  0  0  0  0  0  0  0
  65.2617   66.0655   60.3415 C   0  0  0  0  0  0  0  0  0  0  0  0
  64.9442   65.1515   61.5161 C   0  0  0  0  0  0  0  0  0  0  0  0
  63.8221   64.2439   61.2367 N   0  0  0  0  0  0  0  0  0  0  0  0
  62.8856   64.6262   61.3003 H   0  0  0  0  0  0  0  0  0  0  0  0
  63.9266   62.9847   60.7403 C   0  0  0  0  0  0  0  0  0  0  0  0
  62.8364   62.1869   60.7401 N   0  0  0  0  0  0  0  0  0  0  0  0
  62.8770   61.2450   60.3717 H   0  0  0  0  0  0  0  0  0  0  0  0
  61.9602   62.4885   61.1469 H   0  0  0  0  0  0  0  0  0  0  0  0
  65.0812   62.4910   60.2410 N   0  0  0  0  0  0  0  0  0  0  0  0
  65.9461   63.0156   60.2910 H   0  0  0  0  0  0  0  0  0  0  0  0
  65.1278   61.5630   59.8392 H   0  0  0  0  0  0  0  0  0  0  0  0
```

63.7577 67.7104 55.2206 C 0 0 0 0 0 0 0 0 0 0 0 0
63.3635 68.1554 56.5956 C 0 0 0 0 0 0 0 0 0 0 0 0
62.7159 67.3668 57.6019 C 0 0 0 0 0 0 0 0 0 0 0 0
62.5044 68.1916 58.7071 C 0 0 0 0 0 0 0 0 0 0 0 0
63.0198 69.4278 58.3943 N 0 0 0 0 0 0 0 0 0 0 0 0
63.0045 70.2269 59.0135 H 0 0 0 0 0 0 0 0 0 0 0 0
63.5514 69.4139 57.1324 C 0 0 0 0 0 0 0 0 0 0 0 0
62.2822 66.0257 57.6725 C 0 0 0 0 0 0 0 0 0 0 0 0
61.6496 65.5634 58.8322 C 0 0 0 0 0 0 0 0 0 0 0 0
61.4537 66.4128 59.9189 C 0 0 0 0 0 0 0 0 0 0 0 0
61.8769 67.7447 59.8763 C 0 0 0 0 0 0 0 0 0 0 0 0
70.2477 70.3137 51.3661 C 0 0 0 0 0 0 0 0 0 0 0 0
69.3123 70.3286 50.1465 C 0 0 0 0 0 0 0 0 0 0 0 0
68.2864 71.4585 50.1129 C 0 0 0 0 0 0 0 0 0 0 0 0
66.9063 70.9601 50.2308 N 0 0 0 0 0 0 0 0 0 0 0 0
66.6735 70.1514 49.6690 H 0 0 0 0 0 0 0 0 0 0 0 0
65.9583 71.3812 51.1038 C 0 0 0 0 0 0 0 0 0 0 0 0
65.9727 71.0849 52.4162 N 0 0 0 0 0 0 0 0 0 0 0 0
66.7909 70.6372 52.8696 H 0 0 0 0 0 0 0 0 0 0 0 0
65.2321 71.3815 53.0365 H 0 0 0 0 0 0 0 0 0 0 0 0
64.9388 72.1417 50.6359 N 0 0 0 0 0 0 0 0 0 0 0 0
64.2198 72.4983 51.2530 H 0 0 0 0 0 0 0 0 0 0 0 0
64.8593 72.3847 49.6568 H 0 0 0 0 0 0 0 0 0 0 0 0
67.1416 72.6521 55.2347 C 0 0 0 0 0 0 0 0 0 0 0 0
67.7570 73.9796 54.9376 C 0 0 0 0 0 0 0 0 0 0 0 0
67.8946 74.5795 53.6443 C 0 0 0 0 0 0 0 0 0 0 0 0
68.4677 75.8367 53.8287 C 0 0 0 0 0 0 0 0 0 0 0 0
68.6963 75.9835 55.1764 N 0 0 0 0 0 0 0 0 0 0 0 0
69.1127 76.7991 55.6010 H 0 0 0 0 0 0 0 0 0 0 0 0
68.2786 74.8700 55.8544 C 0 0 0 0 0 0 0 0 0 0 0 0
67.5712 74.1706 52.3328 C 0 0 0 0 0 0 0 0 0 0 0 0
67.8270 75.0302 51.2600 C 0 0 0 0 0 0 0 0 0 0 0 0
68.3937 76.2833 51.4770 C 0 0 0 0 0 0 0 0 0 0 0 0
68.7239 76.7113 52.7668 C 0 0 0 0 0 0 0 0 0 0 0 0
1 2 1 0 0 0
1 3 1 0 0 0
1 33 1 0 0 0
3 35 1 0 0 0
3 4 1 0 0 0
35 36 1 0 0 0
36 37 1 0 0 0
37 38 1 0 0 0
38 40 1 0 0 0
38 39 1 0 0 0
40 41 1 0 0 0
40 44 1 0 0 0
41 42 1 0 0 0
41 43 1 0 0 0
44 45 1 0 0 0
44 46 1 0 0 0

4	5	2	0	0	0
4	6	1	0	0	0
6	7	1	0	0	0
6	8	1	0	0	0
8	47	1	0	0	0
8	9	1	0	0	0
47	48	1	0	0	0
48	49	1	0	0	0
48	53	2	0	0	0
9	10	2	0	0	0
9	11	1	0	0	0
49	50	2	0	0	0
49	54	1	0	0	0
50	51	1	0	0	0
50	57	1	0	0	0
51	52	1	0	0	0
51	53	1	0	0	0
54	55	2	0	0	0
55	56	1	0	0	0
56	57	2	0	0	0
11	12	1	0	0	0
11	15	1	0	0	0
12	13	1	0	0	0
13	14	1	0	0	0
14	15	1	0	0	0
15	16	1	0	0	0
16	17	2	0	0	0
16	18	1	0	0	0
18	19	1	0	0	0
18	20	1	0	0	0
20	58	1	0	0	0
20	21	1	0	0	0
58	59	1	0	0	0
59	60	1	0	0	0
60	61	1	0	0	0
61	63	1	0	0	0
61	62	1	0	0	0
63	64	1	0	0	0
63	67	1	0	0	0
64	65	1	0	0	0
64	66	1	0	0	0
67	68	1	0	0	0
67	69	1	0	0	0
21	22	2	0	0	0
21	23	1	0	0	0
23	24	1	0	0	0
23	25	1	0	0	0
25	70	1	0	0	0
25	26	1	0	0	0
70	71	1	0	0	0

```
 71 72  1  0  0  0
 71 76  2  0  0  0
 26 27  2  0  0  0
 26 28  1  0  0  0
 72 73  2  0  0  0
 72 77  1  0  0  0
 73 74  1  0  0  0
 73 80  1  0  0  0
 74 75  1  0  0  0
 74 76  1  0  0  0
 77 78  2  0  0  0
 78 79  1  0  0  0
 79 80  2  0  0  0
 28 29  1  0  0  0
 28 32  1  0  0  0
 29 30  1  0  0  0
 30 31  1  0  0  0
 31 32  1  0  0  0
 32 33  1  0  0  0
 33 34  2  0  0  0
M  END
> <Score>
-9.43

$$$$
```
