# Supplementary material for: AOA-2 Derivatives as Outer Membrane Protein A Inhibitors for Treatment of Gram-Negative Bacilli Infections
Source: Front Microbiol. 2021 Feb 12;12:634323. doi: 10.3389/fmicb.2021.634323 (PMC7907166; doi:10.3389/fmicb.2021.634323)
Supplement: Supplementary file 3 [file Data_Sheet_3.PDF]

RW01

```
 84 90  0  0  0  0  0  0  0  0999 V2000
   66.7092   68.2407   58.7048 N   0  0  0  0  0  0  0  0  0  0  0  0
   65.8307   68.6438   58.3962 H   0  0  0  0  0  0  0  0  0  0  0  0
   66.9367   66.8273   58.4520 C   0  0  0  0  0  0  0  0  0  0  0  0
   66.1320   66.3639   57.2209 C   0  0  0  0  0  0  0  0  0  0  0  0
   65.8123   65.1854   57.0582 O   0  0  0  0  0  0  0  0  0  0  0  0
   65.9220   67.3431   56.2621 N   0  0  0  0  0  0  0  0  0  0  0  0
   66.3010   68.2920   56.3936 H   0  0  0  0  0  0  0  0  0  0  0  0
   65.1610   67.0743   55.0421 C   0  0  0  0  0  0  0  0  0  0  0  0
   65.9272   67.6749   53.8527 C   0  0  0  0  0  0  0  0  0  0  0  0
   66.2331   68.8740   53.8390 O   0  0  0  0  0  0  0  0  0  0  0  0
   66.2681   66.8532   52.7897 N   0  0  0  0  0  0  0  0  0  0  0  0
   65.9481   65.4381   52.6228 C   0  0  0  0  0  0  0  0  0  0  0  0
   65.9451   65.2446   51.1064 C   0  0  0  0  0  0  0  0  0  0  0  0
   64.6314   65.6910   50.5404 N   0  0  0  0  0  0  0  0  0  0  0  0
   64.3749   66.6347   50.8681 H   0  0  0  0  0  0  0  0  0  0  0  0
   63.8678   65.0503   50.8066 H   0  0  0  0  0  0  0  0  0  0  0  0
   64.6473   65.7043   49.5091 H   0  0  0  0  0  0  0  0  0  0  0  0
   67.0394   66.1900   50.6304 C   0  0  0  0  0  0  0  0  0  0  0  0
   67.0011   67.3705   51.6206 C   0  0  0  0  0  0  0  0  0  0  0  0
   68.4263   67.8596   51.9537 C   0  0  0  0  0  0  0  0  0  0  0  0
   69.4284   67.1593   51.8509 O   0  0  0  0  0  0  0  0  0  0  0  0
   68.4596   69.1659   52.4003 N   0  0  0  0  0  0  0  0  0  0  0  0
   67.5625   69.6208   52.5837 H   0  0  0  0  0  0  0  0  0  0  0  0
   69.7021   69.8947   52.6517 C   0  0  0  0  0  0  0  0  0  0  0  0
   69.4793   71.0357   53.6582 C   0  0  0  0  0  0  0  0  0  0  0  0
   70.1906   72.0437   53.6905 O   0  0  0  0  0  0  0  0  0  0  0  0
   68.4856   70.7720   54.5792 N   0  0  0  0  0  0  0  0  0  0  0  0
   67.9715   69.8881   54.5354 H   0  0  0  0  0  0  0  0  0  0  0  0
   68.0994   71.7163   55.6245 C   0  0  0  0  0  0  0  0  0  0  0  0
   67.4783   70.8908   56.7677 C   0  0  0  0  0  0  0  0  0  0  0  0
   66.6220   70.0281   56.5282 O   0  0  0  0  0  0  0  0  0  0  0  0
   67.9051   71.1470   58.0568 N   0  0  0  0  0  0  0  0  0  0  0  0
   68.9913   72.0349   58.4556 C   0  0  0  0  0  0  0  0  0  0  0  0
   69.1739   71.7996   59.9468 C   0  0  0  0  0  0  0  0  0  0  0  0
   67.7901   71.3577   60.4074 C   0  0  0  0  0  0  0  0  0  0  0  0
   67.2569   70.5495   59.2264 C   0  0  0  0  0  0  0  0  0  0  0  0
   67.6373   69.0611   59.3148 C   0  0  0  0  0  0  0  0  0  0  0  0
   68.6972   68.6472   59.7741 O   0  0  0  0  0  0  0  0  0  0  0  0
   66.6392   65.9513   59.6773 C   0  0  0  0  0  0  0  0  0  0  0  0
   65.2392   66.1827   60.2564 C   0  0  0  0  0  0  0  0  0  0  0  0
   64.9026   65.2472   61.4083 C   0  0  0  0  0  0  0  0  0  0  0  0
   63.8327   64.2982   61.0684 N   0  0  0  0  0  0  0  0  0  0  0  0
   62.9121   64.6795   60.8830 H   0  0  0  0  0  0  0  0  0  0  0  0
   63.9986   62.9746   60.8169 C   0  0  0  0  0  0  0  0  0  0  0  0
   63.0103   62.1147   61.1468 N   0  0  0  0  0  0  0  0  0  0  0  0
   63.0981   61.1225   60.9669 H   0  0  0  0  0  0  0  0  0  0  0  0
```

```
62.1744 62.4275 61.6239 H 0 0 0 0 0 0 0 0 0 0 0 0
65.1171 62.4763 60.2456 N 0 0 0 0 0 0 0 0 0 0 0 0
65.9170 63.0645 60.0479 H 0 0 0 0 0 0 0 0 0 0 0 0
65.2064 61.4907 60.0327 H 0 0 0 0 0 0 0 0 0 0 0 0
63.7647 67.7231 55.0745 C 0 0 0 0 0 0 0 0 0 0 0 0
63.3778 68.2324 56.4281 C 0 0 0 0 0 0 0 0 0 0 0 0
62.7376 67.4875 57.4715 C 0 0 0 0 0 0 0 0 0 0 0 0
62.5308 68.3595 58.5402 C 0 0 0 0 0 0 0 0 0 0 0 0
63.0421 69.5816 58.1719 N 0 0 0 0 0 0 0 0 0 0 0 0
63.0284 70.4048 58.7595 H 0 0 0 0 0 0 0 0 0 0 0 0
63.5649 69.5142 56.9075 C 0 0 0 0 0 0 0 0 0 0 0 0
62.3074 66.1501 57.6036 C 0 0 0 0 0 0 0 0 0 0 0 0
61.6805 65.7392 58.7851 C 0 0 0 0 0 0 0 0 0 0 0 0
61.4888 66.6352 59.8342 C 0 0 0 0 0 0 0 0 0 0 0 0
61.9107 67.9640 59.7311 C 0 0 0 0 0 0 0 0 0 0 0 0
70.3652 70.3768 51.3473 C 0 0 0 0 0 0 0 0 0 0 0 0
71.7526 69.7866 51.0478 C 0 0 0 0 0 0 0 0 0 0 0 0
72.9337 70.7205 51.2959 C 0 0 0 0 0 0 0 0 0 0 0 0
74.2124 69.9952 51.3149 N 0 0 0 0 0 0 0 0 0 0 0 0
74.9224 70.3239 50.6729 H 0 0 0 0 0 0 0 0 0 0 0 0
74.5943 69.0299 52.1875 C 0 0 0 0 0 0 0 0 0 0 0 0
74.6992 67.7290 51.8607 N 0 0 0 0 0 0 0 0 0 0 0 0
74.6398 67.4019 50.8793 H 0 0 0 0 0 0 0 0 0 0 0 0
74.9498 67.0281 52.5443 H 0 0 0 0 0 0 0 0 0 0 0 0
74.8879 69.3889 53.4612 N 0 0 0 0 0 0 0 0 0 0 0 0
75.1992 68.7089 54.1436 H 0 0 0 0 0 0 0 0 0 0 0 0
74.8130 70.3474 53.7769 H 0 0 0 0 0 0 0 0 0 0 0 0
67.0708 72.7128 55.0670 C 0 0 0 0 0 0 0 0 0 0 0 0
67.6804 74.0168 54.6728 C 0 0 0 0 0 0 0 0 0 0 0 0
67.6526 74.6088 53.3688 C 0 0 0 0 0 0 0 0 0 0 0 0
68.2844 75.8479 53.4622 C 0 0 0 0 0 0 0 0 0 0 0 0
68.7059 75.9903 54.7629 N 0 0 0 0 0 0 0 0 0 0 0 0
69.2029 76.7932 55.1194 H 0 0 0 0 0 0 0 0 0 0 0 0
68.3541 74.8924 55.5005 C 0 0 0 0 0 0 0 0 0 0 0 0
67.1346 74.2081 52.1187 C 0 0 0 0 0 0 0 0 0 0 0 0
67.2612 75.0579 51.0153 C 0 0 0 0 0 0 0 0 0 0 0 0
67.8909 76.2931 51.1423 C 0 0 0 0 0 0 0 0 0 0 0 0
68.4141 76.7124 52.3695 C 0 0 0 0 0 0 0 0 0 0 0 0
 1  2  1  0  0  0
 1  3  1  0  0  0
 1 37  1  0  0  0
 3 39  1  0  0  0
 3  4  1  0  0  0
39 40  1  0  0  0
40 41  1  0  0  0
41 42  1  0  0  0
42 44  1  0  0  0
42 43  1  0  0  0
44 45  1  0  0  0
44 48  1  0  0  0
```

45 46 1 0 0 0
45 47 1 0 0 0
48 49 1 0 0 0
48 50 1 0 0 0
4 5 2 0 0 0
4 6 1 0 0 0
6 7 1 0 0 0
6 8 1 0 0 0
8 51 1 0 0 0
8 9 1 0 0 0
51 52 1 0 0 0
52 53 1 0 0 0
52 57 2 0 0 0
9 10 2 0 0 0
9 11 1 0 0 0
53 54 2 0 0 0
53 58 1 0 0 0
54 55 1 0 0 0
54 61 1 0 0 0
55 56 1 0 0 0
55 57 1 0 0 0
58 59 2 0 0 0
59 60 1 0 0 0
60 61 2 0 0 0
11 12 1 0 0 0
11 19 1 0 0 0
12 13 1 0 0 0
13 14 1 0 0 0
13 18 1 0 0 0
14 15 1 0 0 0
14 16 1 0 0 0
14 17 1 0 0 0
18 19 1 0 0 0
19 20 1 0 0 0
20 21 2 0 0 0
20 22 1 0 0 0
22 23 1 0 0 0
22 24 1 0 0 0
24 62 1 0 0 0
24 25 1 0 0 0
62 63 1 0 0 0
63 64 1 0 0 0
64 65 1 0 0 0
65 67 1 0 0 0
65 66 1 0 0 0
67 68 1 0 0 0
67 71 1 0 0 0
68 69 1 0 0 0
68 70 1 0 0 0
71 72 1 0 0 0

```
 71 73  1  0  0  0
 25 26  2  0  0  0
 25 27  1  0  0  0
 27 28  1  0  0  0
 27 29  1  0  0  0
 29 74  1  0  0  0
 29 30  1  0  0  0
 74 75  1  0  0  0
 75 76  1  0  0  0
 75 80  2  0  0  0
 30 31  2  0  0  0
 30 32  1  0  0  0
 76 77  2  0  0  0
 76 81  1  0  0  0
 77 78  1  0  0  0
 77 84  1  0  0  0
 78 79  1  0  0  0
 78 80  1  0  0  0
 81 82  2  0  0  0
 82 83  1  0  0  0
 83 84  2  0  0  0
 32 33  1  0  0  0
 32 36  1  0  0  0
 33 34  1  0  0  0
 34 35  1  0  0  0
 35 36  1  0  0  0
 36 37  1  0  0  0
 37 38  2  0  0  0
M  CHG 1  14   1
M  END
> <Score>
-9.51

$$$$
```
