# Supplementary material for: AOA-2 Derivatives as Outer Membrane Protein A Inhibitors for Treatment of Gram-Negative Bacilli Infections
Source: Front Microbiol. 2021 Feb 12;12:634323. doi: 10.3389/fmicb.2021.634323 (PMC7907166; doi:10.3389/fmicb.2021.634323)
Supplement: Supplementary file 4 [file Data_Sheet_4.PDF]

RW06

```
68 74  0  0  1  0  0  0  0  0999 V2000
   68.9500   68.2240   58.3590 N   0  0  0  0  0  0  0  0  0  0  0  0
   67.9730   68.5000   58.4100 H   0  0  0  0  0  0  0  0  0  0  0  0
   69.2700   66.8060   58.2920 C   0  0  2  0  0  0  0  0  0  0  0  0
   68.0540   66.0510   57.7240 C   0  0  0  0  0  0  0  0  0  0  0  0
   67.7580   64.9250   58.1380 O   0  0  0  0  0  0  0  0  0  0  0  0
   67.3970   66.7010   56.6990 N   0  0  0  0  0  0  0  0  0  0  0  0
   67.6930   67.6490   56.4170 H   0  0  0  0  0  0  0  0  0  0  0  0
   66.2570   66.1220   55.9810 C   0  0  2  0  0  0  0  0  0  0  0  0
   66.2880   66.6350   54.5310 C   0  0  0  0  0  0  0  0  0  0  0  0
   66.5650   67.8140   54.2850 O   0  0  0  0  0  0  0  0  0  0  0  0
   65.9330   65.7630   53.5220 N   0  0  0  0  0  0  0  0  0  0  0  0
   65.7310   64.3250   53.6310 C   0  0  0  0  0  0  0  0  0  0  0  0
   65.6660   63.8340   52.1940 C   0  0  0  0  0  0  0  0  0  0  0  0
   65.0890   65.0270   51.4380 C   0  0  0  0  0  0  0  0  0  0  0  0
   65.6830   66.2380   52.1570 C   0  0  2  0  0  0  0  0  0  0  0  0
   66.9960   66.7200   51.5130 C   0  0  0  0  0  0  0  0  0  0  0  0
   67.8510   65.9660   51.0570 O   0  0  0  0  0  0  0  0  0  0  0  0
   67.1760   68.0900   51.6220 N   0  0  0  0  0  0  0  0  0  0  0  0
   66.4080   68.6430   52.0010 H   0  0  0  0  0  0  0  0  0  0  0  0
   68.2870   68.5760   51.0490 C   0  0  2  0  0  0  0  0  0  0  0  0
   68.5140   70.0370   52.1020 C   0  0  0  0  0  0  0  0  0  0  0  0
   68.9730   71.0960   51.6190 O   0  0  0  0  0  0  0  0  0  0  0  0
   68.1260   69.9380   53.4300 N   0  0  0  0  0  0  0  0  0  0  0  0
   67.6850   69.0620   53.7550 H   0  0  0  0  0  0  0  0  0  0  0  0
   68.2470   70.7740   54.3760 C   0  0  2  0  0  0  0  0  0  0  0  0
   68.4070   70.3930   55.8320 C   0  0  0  0  0  0  0  0  0  0  0  0
   67.7640   69.3940   56.1730 O   0  0  0  0  0  0  0  0  0  0  0  0
   69.2540   71.0180   56.7270 N   0  0  0  0  0  0  0  0  0  0  0  0
   70.1780   72.1110   56.4560 C   0  0  0  0  0  0  0  0  0  0  0  0
   71.0550   72.1980   57.6950 C   0  0  0  0  0  0  0  0  0  0  0  0
   70.1400   71.7070   58.8110 C   0  0  0  0  0  0  0  0  0  0  0  0
   69.2930   70.6270   58.1400 C   0  0  2  0  0  0  0  0  0  0  0  0
   69.9060   69.2210   58.2840 C   0  0  0  0  0  0  0  0  0  0  0  0
   71.1100   68.9890   58.2280 O   0  0  0  0  0  0  0  0  0  0  0  0
   69.7660   66.2430   59.6380 C   0  0  0  0  0  0  0  0  0  0  0  0
   71.2700   66.3630   59.8760 C   0  0  0  0  0  0  0  0  0  0  0  0
   71.6140   67.7040   60.4130 N   0  3  0  0  0  0  0  0  0  0  0  0
   71.3630   67.8080   61.4030 H   0  0  0  0  0  0  0  0  0  0  0  0
   71.0980   68.4660   59.8980 H   0  0  0  0  0  0  0  0  0  0  0  0
   72.6150   67.9080   60.3050 H   0  0  0  0  0  0  0  0  0  0  0  0
   64.9090   66.5690   56.5750 C   0  0  0  0  0  0  0  0  0  0  0  0
   64.7900   68.0460   56.7880 C   0  0  0  0  0  0  0  0  0  0  0  0
   65.1300   68.7730   57.9740 C   0  0  0  0  0  0  0  0  0  0  0  0
   64.8630   70.1210   57.7290 C   0  0  0  0  0  0  0  0  0  0  0  0
   64.4170   70.2160   56.4310 N   0  0  0  0  0  0  0  0  0  0  0  0
   64.1850   71.0820   55.9620 H   0  0  0  0  0  0  0  0  0  0  0  0
```

```
   64.3420   68.9710   55.8670 C   0  0  0  0  0  0  0  0  0  0  0  0
   65.6470   68.4070   59.2350 C   0  0  0  0  0  0  0  0  0  0  0  0
   65.8620   69.3930   60.2040 C   0  0  0  0  0  0  0  0  0  0  0  0
   65.5760   70.7290   59.9320 C   0  0  0  0  0  0  0  0  0  0  0  0
   65.0730   71.1200   58.6870 C   0  0  0  0  0  0  0  0  0  0  0  0
   68.6920   68.9390   49.8380 C   0  0  0  0  0  0  0  0  0  0  0  0
   67.6810   69.7100   48.9530 C   0  0  0  0  0  0  0  0  0  0  0  0
   67.7000   71.1390   49.3040 N   0  3  0  0  0  0  0  0  0  0  0  0
   68.5690   71.6130   49.0270 H   0  0  0  0  0  0  0  0  0  0  0  0
   67.6090   71.2840   50.3430 H   0  0  0  0  0  0  0  0  0  0  0  0
   66.9200   71.6330   48.8530 H   0  0  0  0  0  0  0  0  0  0  0  0
   67.0950   72.0100   54.3910 C   0  0  0  0  0  0  0  0  0  0  0  0
   67.3280   73.2000   53.5040 C   0  0  0  0  0  0  0  0  0  0  0  0
   68.0860   74.3740   53.8230 C   0  0  0  0  0  0  0  0  0  0  0  0
   68.1090   75.1790   52.6830 C   0  0  0  0  0  0  0  0  0  0  0  0
   67.3460   74.5500   51.7280 N   0  0  0  0  0  0  0  0  0  0  0  0
   67.1720   74.9140   50.8020 H   0  0  0  0  0  0  0  0  0  0  0  0
   66.8600   73.3670   52.2140 C   0  0  0  0  0  0  0  0  0  0  0  0
   68.7640   74.8230   54.9770 C   0  0  0  0  0  0  0  0  0  0  0  0
   69.4600   76.0360   54.9400 C   0  0  0  0  0  0  0  0  0  0  0  0
   69.4820   76.8040   53.7790 C   0  0  0  0  0  0  0  0  0  0  0  0
   68.8030   76.3940   52.6280 C   0  0  0  0  0  0  0  0  0  0  0  0
  1  2  1  0  0  0  0
  1  3  1  0  0  0  0
  1 33  1  0  0  0  0
  3  4  1  0  0  0  0
  3 35  1  1  0  0  0
  4  5  2  0  0  0  0
  4  6  1  0  0  0  0
  6  7  1  0  0  0  0
  6  8  1  0  0  0  0
  8  9  1  0  0  0  0
  8 41  1  1  0  0  0
  9 10  2  0  0  0  0
  9 11  1  0  0  0  0
 11 12  1  0  0  0  0
 11 15  1  0  0  0  0
 12 13  1  0  0  0  0
 13 14  1  0  0  0  0
 15 14  1  6  0  0  0
 15 16  1  0  0  0  0
 16 17  2  0  0  0  0
 16 18  1  0  0  0  0
 18 19  1  0  0  0  0
 18 20  1  0  0  0  0
 20 21  1  0  0  0  0
 20 52  1  6  0  0  0
 21 22  2  0  0  0  0
 21 23  1  0  0  0  0
 23 24  1  0  0  0  0
```

```
 23 25  1  0  0  0  0
 25 26  1  0  0  0  0
 25 58  1  1  0  0  0
 26 27  2  0  0  0  0
 26 28  1  0  0  0  0
 28 29  1  0  0  0  0
 28 32  1  0  0  0  0
 29 30  1  0  0  0  0
 30 31  1  0  0  0  0
 32 31  1  1  0  0  0
 32 33  1  0  0  0  0
 33 34  2  0  0  0  0
 35 36  1  0  0  0  0
 36 37  1  0  0  0  0
 37 38  1  0  0  0  0
 37 39  1  0  0  0  0
 37 40  1  0  0  0  0
 41 42  1  0  0  0  0
 42 43  1  0  0  0  0
 42 47  2  0  0  0  0
 43 44  2  0  0  0  0
 43 48  1  0  0  0  0
 44 45  1  0  0  0  0
 44 51  1  0  0  0  0
 45 46  1  0  0  0  0
 45 47  1  0  0  0  0
 48 49  2  0  0  0  0
 49 50  1  0  0  0  0
 50 51  2  0  0  0  0
 52 53  1  0  0  0  0
 53 54  1  0  0  0  0
 54 55  1  0  0  0  0
 54 56  1  0  0  0  0
 54 57  1  0  0  0  0
 58 59  1  0  0  0  0
 59 60  1  0  0  0  0
 59 64  2  0  0  0  0
 60 61  2  0  0  0  0
 60 65  1  0  0  0  0
 61 62  1  0  0  0  0
 61 68  1  0  0  0  0
 62 63  1  0  0  0  0
 62 64  1  0  0  0  0
 65 66  2  0  0  0  0
 66 67  1  0  0  0  0
 67 68  2  0  0  0  0
M  CHG  2  37   1  54   1
M  END
> <Score>
-9.39
```

$$$$
