# Supplementary figures and images for: AOA-2 Derivatives as Outer Membrane Protein A Inhibitors for Treatment of Gram-Negative Bacilli Infections
Source: Front Microbiol. 2021 Feb 12;12:634323. doi: 10.3389/fmicb.2021.634323 (PMC7907166; doi:10.3389/fmicb.2021.634323)

## Slide 1
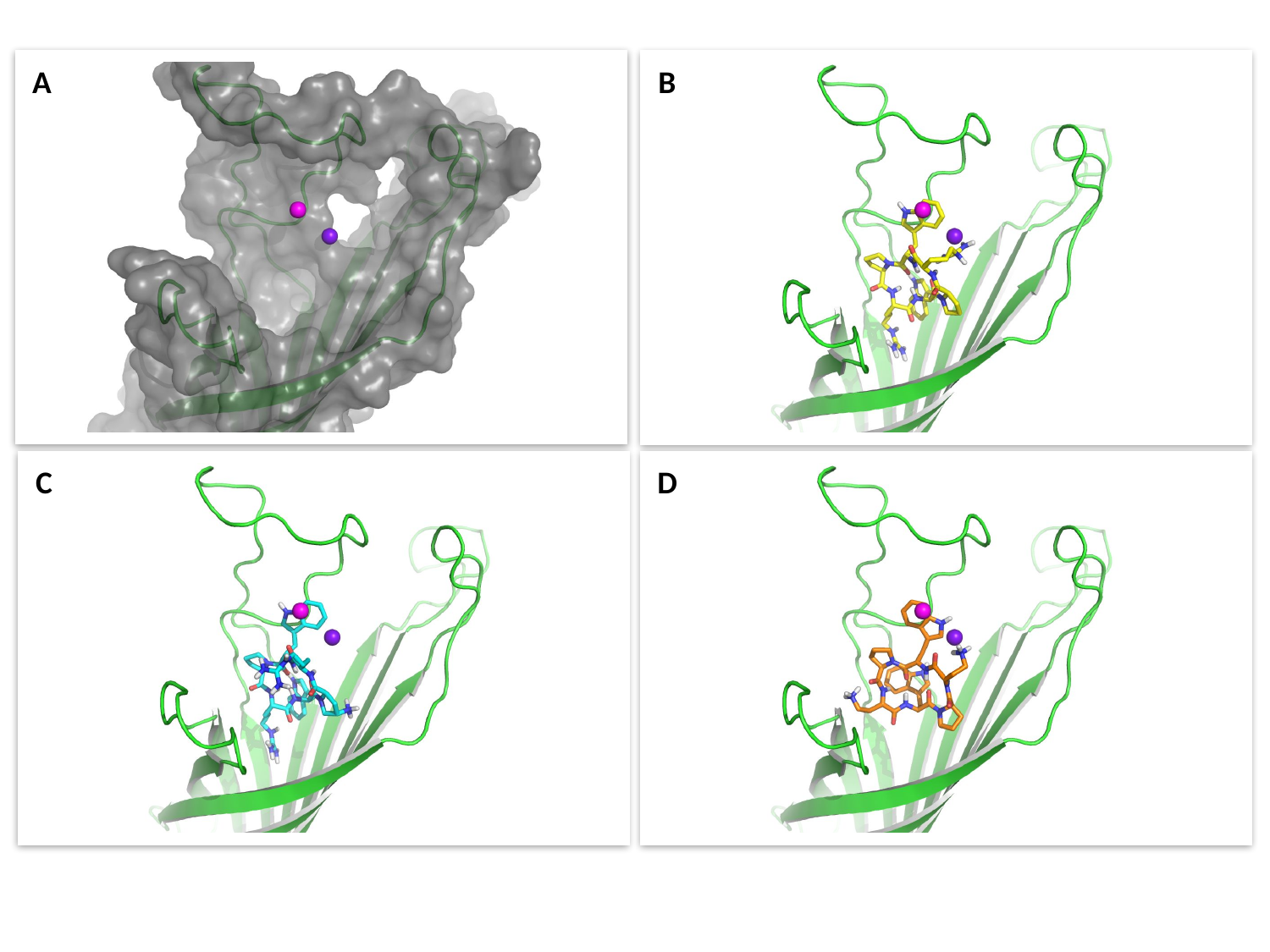

A
B
C
D

Supplement: Supplementary file 5 [file Presentation_1.PPTX]
